# Association of *TLR2* T597C, TLR4 A896G, and TLR9 G1174A gene polymorphisms with risk of pulmonary tuberculosis in the Ethiopian population: A case-control study

**DOI:** 10.1371/journal.pone.0361059

**Published:** 2026-10-30

**Authors:** Addisu Melake, Kefiyalew Mihrete, Birhanemaskal Malkamu

**Affiliations:** 1 Biomedical Science Education and Service Directorate, College of Health Science, Debre Tabor University, Debre Tabor, Ethiopia; 2 Department of Medical Laboratory Sciences, College of Health Science, Debre Tabor University, Debre Tabor, Ethiopia; Rutgers Biomedical and Health Sciences, UNITED STATES OF AMERICA

## Abstract

**Background:**

Toll-like receptor genes have been extensively researched for their role in the susceptibility to pulmonary tuberculosis across different populations. The relationship between variations in toll-like receptor genes and additional risk factors for pulmonary tuberculosis is not well understood, because of the complex interplay between genetic predispositions and environmental influences. This study aimed to elucidate the association of *TLR2* T597C, *TLR4* A896G, and *TLR9* G1174A gene polymorphisms with the risk of developing pulmonary tuberculosis.

**Methods:**

A total of 306 samples – 153 patients with pulmonary tuberculosis and 153 age- and sex-matched healthy controls were selected using a consecutive sampling technique. Clinical and biochemical variables were measured to assess the associated risk factors. Deoxyribonucleic acid was isolated from blood samples, and the *TLR2*, *TLR4,* and *TLR9* genotypes were identified using polymerase chain reaction–restriction fragment length polymorphism and analyzed by agarose gel electrophoresis.

**Results:**

Our analysis showed that the *TLR2*-CC genotype (OR  = 3.82; 95% CI = 1.79–8.16; P < 0.001) and C allele (OR=2.12; 95%CI: 1.42–3.10; P < 0.001), the *TLR4*-GG genotype (OR=3.18; 95%CI: 1.71–5.92; P < 0.001) and G allele (OR=2.25; 95%CI: 1.61–3.14; P < 0.001), and the *TLR9*-AA genotype (OR=2.00; 95%CI: 1.09–3.68; P = 0.025) and A allele (OR=1.60; 95%CI: 1.15–2.21; P < 0.001) is significantly associated in patients than in controls, suggesting their potential role as genetic risk factors for the development of pulmonary tuberculosis. Combined genotype analysis revealed that individuals carrying all three risk genotypes (CC-GG-AA) significantly associated with increased PTB risk. Multivariable analysis showed that HIV co-infection and malnutrition were independent predictors of pulmonary tuberculosis.

**Conclusion:**

The *TLR2* T597C gene of the CC genotype and the C allele, the *TLR4* A896G genes of the GG genotype and the G allele, and the *TLR9* G1174A genes of the AA genotype and the A allele are associated with increased risk of pulmonary tuberculosis among the Ethiopian patients under study.

## 1. Introduction

Tuberculosis (TB) is an infectious disease primarily resulting from the bacterium Mycobacterium tuberculosis (MTB), with significant implications for public health, especially in developing countries [1]. Pulmonary tuberculosis (PTB), which is the most common form, can lead to various pulmonary dysfunction, ranging from minor abnormalities to severe breathlessness, and can increase the risk of death from respiratory causes [2]. TB is primarily transmitted by respiratory droplets produced by coughing and sneezing, marking it as a highly contagious illness [3]. The global burden of PTB remains significant, with approximately 8.6 million estimated new cases each year, leading to 1.3 million deaths [4]. In Ethiopia, TB incidence was 126 per 100,000 population in 2022, and a meta-analysis conducted in 2024 demonstrates that the pooled prevalence of PTB among populations residing in hotspot settings was 11.7% [5]. TB may be considered a polygenic and multifactorial disease that results from a complex interaction between the host’s genome, pathogen characteristics, and environmental conditions, including social and economic factors [6]. These factors include exposure to TB patients, malnutrition, substance use such as smoking and drug abuse, lifestyle choices like alcohol consumption, and comorbidity such as diabetes and HIV infection [7,8].

Toll-like receptors (TLRs) are crucial elements of the innate immune system, playing a vital role in activating immune cells through the initiation of intracellular signaling pathways [9]. These receptors are present on the membranes of both cytoplasmic and intracellular vesicles, allowing the detection of various molecular patterns from extracellular and intracellular sources [10]. Among the TLRs, *TLR2*, *TLR4,* and *TLR9* are particularly notable for their potential role in immunosurveillance against intracellular bacterial infections, contributing to the body’s defense mechanisms [11]. The TLR2 T597C (rs3804099) polymorphism is an important single nucleotide polymorphism (SNP) located in exon 3 of the TLR2 gene that has been associated with altered susceptibility to various infectious diseases [12,13]. This polymorphism, found specifically in exon 3 of the TLR2 gene located on chromosome 4, features a T to C substitution at nucleotide position 597 in the cDNA sequence [11,14]. This genetic alteration has potential implications for gene expression and mRNA stability, suggesting its relevance in the wider context of human health and disease [15,16].

The *TLR4* is a transmembrane receptor, positioned on the membranes of cytoplasm and vesicles. The *TLR4* A896G (rs4986790), also known as the *TLR4* Asp299Gly gene, is highly conserved, consisting of four exons and three introns, and is located at the chromosomal position 9q33.1 [16].. The *TLR9* G1174A gene polymorphism (officially known as rs352139) is a common genetic variation where the nucleotide guanine (G) is replaced by adenine (A) at position 1174 of the toll-like receptor 9 (TLR-9) gene [17]. This gene, situated on chromosome 3p21.2, contains SNPs located in the non-coding region (intron 1) and has been widely studied for its involvement in immune-related diseases, including PTB [18]. Several subsequent reports have been published on the impact of *TLR2*, *TLR4,* and *TLR9* gene polymorphism on the susceptibility of PTB in different populations [11]. However, the findings from these reports have mostly been inconclusive and inconsistent, leaving uncertainty regarding the relationship of the *TLR2*, *TLR4,* and *TLR9* genotype with PTB, as well as its potential role in increasing or decreasing the risk of the disease [19]. In Ethiopia, extensive research has been conducted examining the prevalence, risk factors, and outcomes associated with PTB [20]. However, there has been a notable lack of studies specifically focusing on the influence of TLR2 gene polymorphism, with only one relevant study conducted to date [21]. Furthermore, there has been no research addressing the role of TLR4 and TLR9 gene polymorphisms in relation to the disease’s development. This gap in the literature highlights an area that requires further investigation to understand the genetic factors contributing to PTB in the Ethiopian context. Thus, this study aims to investigate the relationship of *TLR2* T597C, *TLR4* A896G, and *TLR9* G1174A gene polymorphism with the occurrence of PTB in the Ethiopian population.

## 2. Materials and methods

### 2.1. Study participants

An age- and sex-matched hospital-based case-control study was conducted at Debre Tabor Comprehensive Specialised Hospital (DTCSH) from 27/01/2026 to 06/04/2026. The study included a total of 306 participants of both sexes, comprising 153 PTB patients and 153 healthy controls. Patients with PTB diseases are treated and monitored at the hospital tuberculosis clinic (TBC). All patients who visited TBC were the source population, while the study participants were patients aged 18 and older, newly diagnosed with bacteriologically confirmed PTB, and who had not previously undergone anti-tuberculosis treatment or had received it for less than seven days prior to enrollment. Healthy volunteers who were available during the study period and matched for age and sex served as the controls.

### 2.2. Inclusion and exclusion criteria

Participants in this study were individuals newly diagnosed with PTB, who were all above the age of 18. Their diagnoses were confirmed using GeneXpert MTB/RIF diagnostic tests and smear microscopy methods. In parallel, healthy control subjects were selected, matched for age and sex, and also aged 18 years and above. To rule out active or subclinical infection, spontaneous sputum samples were collected from these individuals and tested using both smear microscopy and the GeneXpert MTB/RIF diagnostic assay. These control participants had negative TB screening results, lacked any previous history of TB, and showed no clinical symptoms indicative of the disease. Recruitment for both groups occurred within the same geographic area to ensure consistency. Patients with prior cases of extrapulmonary TB, latent TB infection, autoimmune diseases, chronic liver or kidney diseases, individuals on long-term corticosteroid therapy, organ transplant recipients, those undergoing cancer treatment, or those with persistent bacterial or viral infections (chronic active infections such as hepatitis B or C, or current secondary bacterial pneumonia as determined by documented clinical history and routine hospital laboratory screenings) were excluded from this study. Furthermore, any individuals who were unable to provide a response or who declined to give informed consent were also excluded from the study. Control participants were determined to be generally healthy based on an unremarkable comprehensive physical examination, the absence of acute or chronic disease symptoms, and normal vital signs during recruitment.

### 2.3. Sample size determination and sampling technique

The sample size was determined using double population proportion formula by taking confidence level of 95%, a power of 80%. To our knowledge, no previous Ethiopian study had evaluated the association between TLR2 T597C, TLR4 A896G, and TLR9 G1174A polymorphisms and PTB, the expected proportions (35% among cases and 20% among controls) were estimated based on effect sizes reported in comparable international case-control studies investigating TLR polymorphisms and PTB. These assumptions were considered reasonable for calculating the minimum required sample size.


$$\begin{array}{c} \text{Sample size }=\;\frac{r+\text{1}}{\text{r}}\;\frac{\;({\text{p}}^{*})(\text{1}-{\text{p}}^{*}){\left({\text{Z}}_{\beta }\;+\;Z_{\alpha /\text{2}}\right)}^{\text{2}}}{{\left({\text{p}}_{\text{1}}-{\text{p}}_{\text{2}}\right)}^{\text{2}}} \end{array}$$

Where: r = ratio of controls to cases (1:1); p_1_ = expected proportion among PTB cases; p_2_ = expected proportion among controls; Zα/2 = standard normal value corresponding to the 95% confidence level (1.96); Zβ = standard normal value corresponding to 80% statistical power (0.84); α = type I error (0.05); and β = type II error (0.20). The final sample size, after adding the 10% non-response rate, was 306 (153 cases and 153 controls) of both sexes. Participants were selected by consecutive sampling methods until the required sample size was reached.

### 2.4. Data collection methods

The PTB risk factors and family history of both patients and healthy control subjects were taken through a semi-structured questionnaire. Body weight was assessed using a portable digital scale, and height was taken using a portable stadiometer. Body mass index (BMI) was then determined by dividing weight in kilograms (kg) by height in metres squared (m²), and participants were categorised as underweight (BMI < 18.5 kg/m²), healthy (18.5–25 kg/m²), overweight (25.0–29.9 kg/m²) or obese (≥ 30 kg/m²).

### 2.5. Sample collection and laboratory methods

For pulmonary tuberculosis diagnostic testing, prospective participants (both suspected cases and healthy controls) were instructed on how to produce a deep cough to obtain a high-quality respiratory specimen. Laboratory professionals collected two consecutive sputum samples (one spot sample and one early morning sample) in sterile, wide-mouthed, leak-proof plastic containers. The collected sputum samples were processed immediately at the DTCSH diagnostic laboratory using automated GeneXpert MTB/RIF assay and Ziehl-Neelsen (ZN) direct smear microscopy to confirm active pulmonary tuberculosis. GeneXpert testing was conducted using the GeneXpert Dx System (GeneXpert System System XVI; Cepheid, Sunnyvale, CA, USA) with Xpert MTB/RIF assay cartridges (GXMTB/RIF-US-10; Cepheid, Sunnyvale, CA, USA) according to the manufacturer’s instructions. Sputum samples were mixed with sample reagent at a 2:1 ratio, incubated at room temperature for 15 minutes, and 2mL was transferred to the cartridge for automated real-time PCR amplification. Direct smear microscopy was performed via Ziehl-Neelsen staining, where heat-fixed sputum smears were stained with carbol fuchsin, decolorized with 3% acid-alcohol, and counterstained with 0.3 methylene blue, following standard WHO guidelines. All participants, including patients and healthy controls had a blood sample of 5 ml taken from the median cubital vein by laboratory professionals following quality control and safety procedures. From the 5 ml sample, 3 ml was retained in the test tube without anticoagulants to allow the blood to clot and subsequently centrifuged to extract the serum. Each serum sample underwent enzymatic analysis for glucose, using the Siemens Dimension EXL 200 fully automated analyser at the DTCSH diagnostic laboratory. Diabetes mellitus was diagnosed if the fasting or random plasma glucose concentration is ≥ 126 mg/dl (7 mmol/L) or ≥200 mg/dl (11.1 mmol/L), respectively, or if the patient was treated with insulin or oral hypoglycemic agents.

### 2.6. *TLR2* T597C, *TLR4* A896G, and *TLR9* G1174A gene analysis

Genomic DNA was extracted from the remaining 2 ml blood samples following the manufacturer’s protocol of the genomic DNA extraction and purification kit (Axygen Biosciences, USA). Purity and concentration were assessed using spectrophotometric measurements at 260 and 280 nm, with quality confirmed via 1% agarose gel electrophoresis. Genotypes were detected using a polymerase chain reaction‑restriction fragment length polymorphism (PCR‑RFLP). The reference sequence, genomic location (GRCh38), annotated region, primer sequences, restriction enzymes and the amplification product fragments sizes of the TLR2, TLR4, and TLR9 polymorphisms are shown in Table 1. The PCR protocol involved a mixture prepared with 25 μl of Maxima Hot Start PCR Master Mix (2X) from Thermo Scientific, alongside 10 μl of the DNA sample, 0.5 μM of each primer, and 14 μl nuclease-free water to achieve a total volume of 50 μl. Nuclease-free water also served as a negative control in each PCR experiment. The PCR cycles were conducted using a G-Storm Thermal Cycler with specific temperature settings that included an initial denaturation at 95°C for 3 minutes, followed by 35 cycles of denaturation at 94°C for 30 seconds, annealing at 58°C for 30 seconds, and extension at 72°C for 30 seconds, with a final extension at 72°C for 10 minutes. After PCR amplification, a 10 μl aliquot of the amplified DNA underwent digestion using the respective restriction enzymes (New England Biolabs, England), according to the manufacturers’ protocol. Gel electrophoresis was conducted on a 2% agarose gel with ethidium bromide, running at 120 V for 1 hour, and visualized using a gel documentation system UV transilluminator (S1 Fig, S2 Fig, and S3 Fig).

**Table 1:** Primer sequences, restriction enzymes and PCR product fragment sizes of the TLR gene polymorphisms.

Gene Polymorphism	Reference Sequence	Genomic location	Annotated region	Primer sequence	Restriction enzyme	PCR product size (bp)
TLR2 T597C (rs3804099)	NM_003264.5	**chr4:153,703,504**	**Exon 3; coding/synonymous variant**	F: 5′- CTT CTC TGG TGC CTT TGT TGG −3′ R: 5′- GAA ATG TGA TGG GGA GAG GG −3′	TaiI	TT (286) TC (286, 184, 102) CC (184, 102)
TLR4 A896G (rs4986790)	NM_138554.5	**chr9:117,713,024**	**Exon 3; missense variant (Asp299Gly)**	F: 5′GATTAGCATACT TA GAC TACTACCTCC ATG-3′ R: 5′-GATCAACTTC TGA AAA AGC T TCCCAC-3′	NcoI	AA (249) AG (249, 223, 26) GG (223, 26)
TLR9 G1174A (rs352139)	NG_033933.1	**chr3:52,258,372**	**Intron 1**	F: 5′-CTTCAGCTGGCTGT TGT CTC-3′ R: 5′-GGAGAGTGCTG TTG TTG GAG-3′	BstUI	GG (135) GA (135, 108, 27) AA (108, 27)

### 2.7. Statistical analysis

Data were analyzed using STATA Version 17. Continuous variables were analyzed as mean ± standard deviation (SD) and compared through one-way ANOVA and post-hoc Tukey’s test for multiple comparisons, while categorical variables were expressed as frequencies and percentages, analyzed using the chi-square (χ²) test. Initially, univariable logistic regression analyses were performed to examine the association between each explanatory variable and PTB. Variables with a p-value <0.20 in the univariable analysis, along with clinically relevant factors like age and sex, were entered into the multivariable logistic regression model to identify factors independently associated with PTB. Adjusted odds ratios (AORs) with 95% confidence intervals were estimated from the final multivariable model. Statistical significance was defined as a p-value of less than 0.05.

### 2.8. Ethics approval and consent to participate

The Institutional Research Ethics Review Committee (IRERC) at Debre Tabor University gave the study protocol approval (DTU/IRERC/OO8/18) on November 05, 2025. Study participants were enrolled only after informed written consent was obtained from each participant. All of the information was collected in an anonymous manner and handled with care. The guiding principles of the Helsinki Declaration were followed throughout the entire data-gathering process.

## 3. Results

### 3.1. Participants characteristics

The demographic, behavioural, and clinical profiles of participants indicated no significant differences in sex (p = 0.6485) and age (p = 0.4954) between groups. However, HIV co-infection rates were significantly higher in the PTB group compared to controls (p < 0.001). Additionally, the PTB group exhibited a markedly lower BMI than the control group (p < 0.001). Other factors such as FBG, family history, smoking habits, and alcohol use showed no significant differences (p > 0.05) across the research groups (Table 2).

**Table 2:** Demographic, behavioural, and clinical characteristics of the study participants.

Variable		PTB (n = 153)	Control (n = 153)	P-value
Sex	Male	72 (47.0%)	76 (49.6%)	0.6485
	Female	81 (52.9%)	77 (50.3%)	
Age (years)		49.5 ± 12.4	48.6 ± 8.2	0.4954
BMI (Kg/m2)		19.6 ± 2.7	23.3 ± 3.6	< 0.001*
FBG (mg/dl)		97.7 ± 22.2	93.5 ± 24.5	0.1125
HIV co-infection	Yes	33 (21.6%)	12 (7.8%)	< 0.001*
	No	120 (78.4%)	141 (92.1%)	
Family history of PTB	Yes	29 (18.9%)	21 (13.7%)	0.2174
	No	124 (81.0%)	132 (86.2%)	
Smoking habit	Yes	11 (7.1%)	7 (4.5%)	0.3327
	No	142 (92.8%)	146 (95.4%)	
Alcohol intake	Yes	64 (41.8%)	52 (33.9%)	0.1584
	No	89 (58.1%)	101 (66.0%)	

Note: ^*^P-value <0.05 is considered statistically significant.

### 3.2. Distribution of *TLR2* T597C, *TLR4* A896G, and *TLR9* G1174A Genotypes and Allele Frequencies

The genotype distribution of *TLR2* T597C, *TLR4* A896G, and *TLR9* G1174A gene polymorphisms shows notable differences between PTB patients and healthy controls (Table 3, Fig 1). Among patients with pulmonary tuberculosis, the genotype frequencies of the TLR2 T597C polymorphism were as follows: homozygous CC (41.8%), heterozygous TC (36.6%), and homozygous TT (21.5%). In the control group, the frequencies were significantly different, with 17.0% for CC, 49.0% for TC, and 34.0% for TT. Statistical analysis revealed that PTB patients had a higher occurrence of the homozygous CC genotype compared to controls (odds ratio [OR] = 3.82; 95% confidence interval [CI] = 1.79–8.16; P < 0.001). The C allele was also more prevalent in PTB patients (OR = 2.12; 95% CI: 1.42–3.10; P < 0.001). For the TLR4 A896G genotypes, PTB patients showed frequencies of 57.5% for homozygous GG, 26.8% for heterozygous AG, and 15.6% for homozygous AA. Conversely, control group frequencies were 30.0% for GG, 43.7% for AG, and 26.1% for AA, indicating a significantly greater presence of homozygous GG in PTB patients (OR = 3.18; 95% CI = 1.71–5.92; P < 0.001). The G allele frequency was higher in PTB patients than controls (OR = 2.25; 95% CI: 1.61–3.14; P < 0.001). Analyzing TLR9 G1174A genotypes, PTB patients had frequencies of 49.6% for homozygous AA, 32.6% for heterozygous GA, and 17.6% for homozygous GG compared to control frequencies of 33.9% for AA, 41.8% for GA, and 24.1% for GG. Statistically, PTB patients exhibited a higher proportion of homozygous AA (OR = 2.00; 95% CI = 1.09–3.68; P = 0.025), with the A allele more common in this group (OR = 1.60; 95% CI: 1.15–2.21; P < 0.001). All three polymorphisms were in Hardy-Weinberg equilibrium in the control group (p > 0.05 for all), indicating that the control population was genetically representative.

**Table 3:** Distribution of *TLR2*, *TLR4*, and *TLR9* genotypes and allele frequencies.

Gene/SNP	Genotype/Allele	PTB (n = 153)	Control (n = 153)	OR (95% CI)	p-value
TLR2 (rs3804099)	CC	64 (41.8%)	26 (17.0%)	3.82 (1.79-8.16)	< 0.001*
	TC	56 (36.6%)	75 (49.0%)	1.17 (0.60-2.28)	0.638
	TT	33 (21.5%)	52 (34.0%)	Reference	
	C	184 (60.1%)	127 (41.5%)	2.12 (1.42-3.10)	< 0.001*
	T	122 (39.9%)	179 (58.5%)	Reference	
TLR4 (rs4986790)	GG	88 (57.5%)	46 (30.0%)	3.18 (1.71-5.92)	< 0.001*
	AG	41 (26.8%)	67 (43.7%)	1.01 (0.53-1.93)	0.952
	AA	24 (15.6%)	40 (26.1%)	Reference	
	G	217 (70.7%)	159 (51.9%)	2.25 (1.61-3.14)	< 0.001*
	A	89 (29.0%)	147 (48.0%)	Reference	
TLR9 (rs352139)	AA	76 (49.6%)	52 (33.9%)	2.00 (1.09-3.68)	0.025*
	GA	50 (32.6%)	64 (41.8%)	1.07 (0.58-1.99)	0.830
	GG	27 (17.6%)	37 (24.1%)	Reference	
	A	202 (66.0%)	168 (55.0%)	1.60 (1.15-2.21)	<0.001*
	G	104 (34.0%)	138 (45.0%)	Reference	

Note: Reference denotes the homozygous wild-type genotype or the major allele used as the baseline comparison group; *P-value <0.05 is considered statistically significant. Abbreviations: CI, Confidence Interval; OR, Odds Ratio.

**Fig 1:**
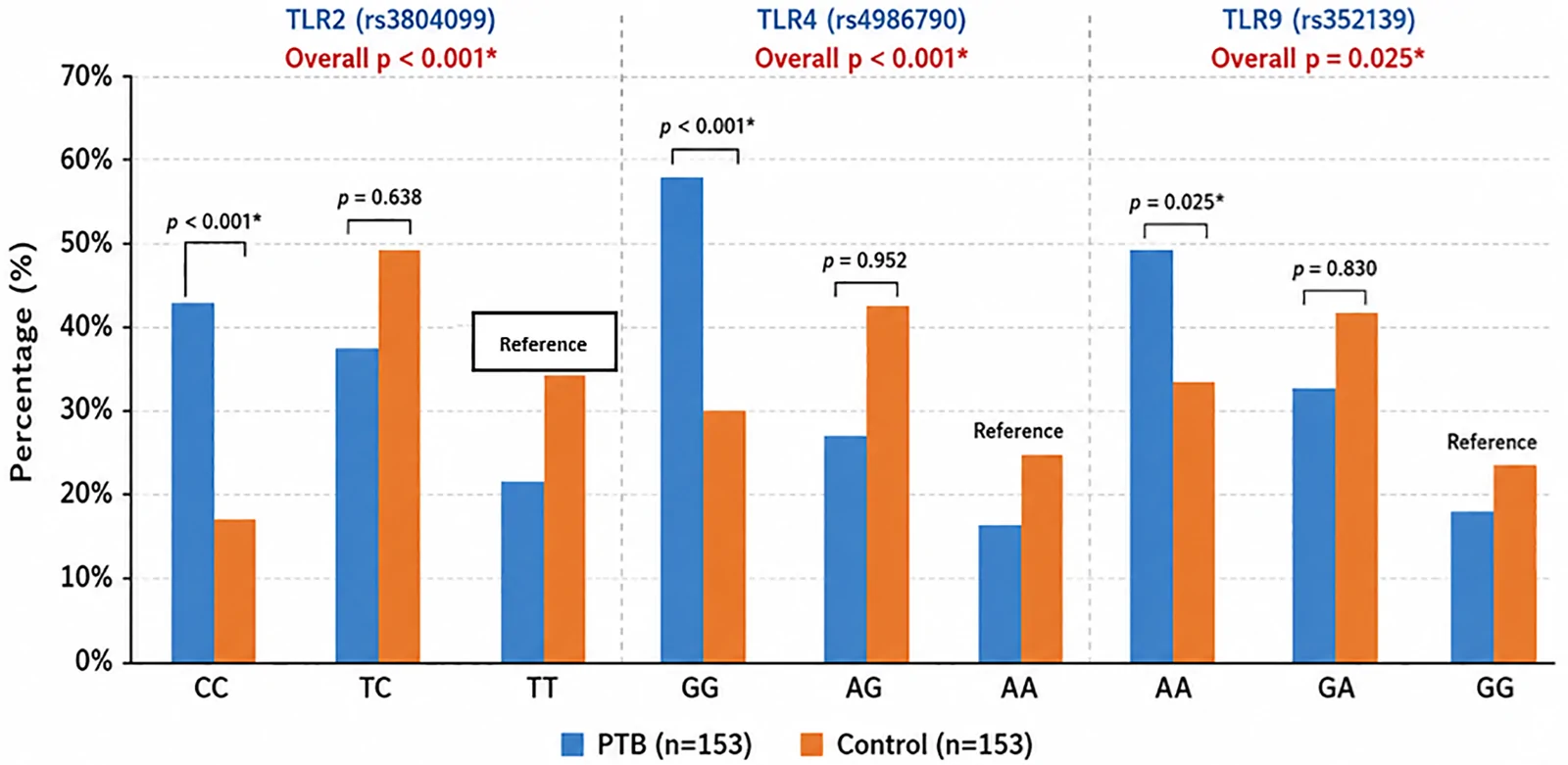
Genotype distribution of TLR2 T597C, TLR4 A896G, and TLR9 G1174A gene polymorphisms in PTB patients and healthy controls. Data are presented as percentages of the total population in each group. “Reference” denotes the homozygous wild-type genotype used as the baseline category in the genotype-level association analysis. *P-value <0.05 is considered statistically significant.

### 3.3. Combined multi-locus genotype analysis of TLR polymorphisms

To evaluate the joint distribution of genetic variations across the three TLR locus, a multilocus genotype analysis was performed using the wild-type combined profile (TT–AA–GG) as the reference category. The combined genotype pattern CC–GG–AA, containing all three homozygous risk variants, was significantly overrepresented in PTB patients compared to healthy controls (28.7% vs. 11.8%). Carrying the CC–GG–AA and CC–GG–GG combinations significantly raises the risk of developing PTB compared to the TT–AA–GG reference group, with odds ratios of 4.89 (P < 0.001) and 2.60 (P = 0.016), respectively. The remaining multilocus genotype combinations did not show statistically significant associations with PTB compared with the TT-AA-GG reference category (P > 0.05).

### 3.4. Multivariable predictors of pulmonary tuberculosis

A multivariable logistic regression analysis was performed to identify independent predictors of PTB (Table 5, Fig 2). After adjusting for potential confounders, HIV co-infection was a potent independent predictor, with HIV-positive individuals being over three times more likely to develop PTB (AOR = 3.12, 95% CI: 1.48–6.58, P = 0.003). Additionally, lower BMI was significantly associated with PTB status (AOR = 0.79, 95% CI: 0.71–0.88, P < 0.001), indicating that for every unit increase in BMI, the likelihood of being in the PTB group decreased by approximately 21%. The analysis also revealed that the TLR2 CC (AOR = 3.74; 95% CI: 1.69–8.29; p = 0.001), TLR4 GG (AOR = 2.97; 95% CI: 1.53–5.76; p = 0.001), and TLR9 AA genotypes (AOR = 1.94; 95% CI: 1.03–3.64; p = 0.039) were independently associated with an increased likelihood of PTB. Other factors, including sex, age, FBG, family history of PTB, smoking habits and alcohol intake, did not show significant predictive value in the adjusted model.

**Fig 2:**
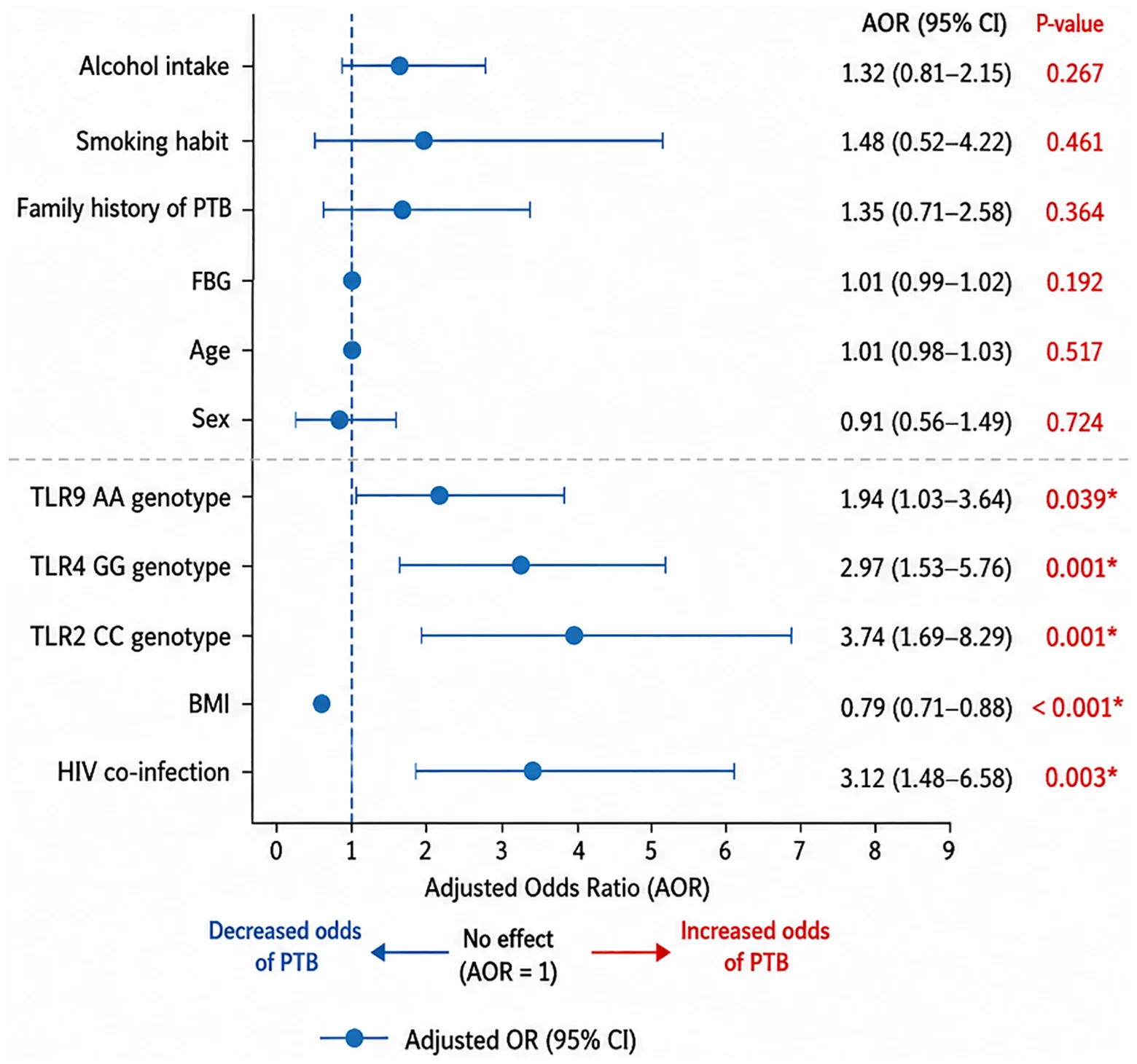
Forest plot of adjusted odds ratios for PTB associated risk factors. The vertical dashed line at AOR = 1 represents no association with PTB risk. Variables with AORs to the right of the reference line indicate increased odds of PTB, whereas those to the left indicate decreased odds. Error bars represent 95% confidence intervals, and *P-value <0.05 is considered statistically significant.

## 4. Discussion

The prevalence of PTB varies greatly between nations, ethnicities, and demographic groups, making it a major worldwide health concern [22]. PTB susceptibility results from a complex interaction between genetic predispositions and environmental factors, as evidenced by research showing that genetic variants within hosts greatly contribute to the likelihood of acquiring tuberculosis [2]. In the present study, the CC genotype and C allele of TLR2 T597C, the GG genotype and G allele of TLR4 A896G, and the AA genotype and A allele of TLR9 G1174A were significantly more frequent in the PTB cohort compared to healthy controls (Table 3, Fig 1). Our findings are consistent with those of other studies conducted in diverse populations, including Ethiopia [21], Sudan [11], Egypt [23], Saudi Arabia [24], Brazil [25], Russia [26], Moldova [27], Finland [28], Pakistan [29], Taiwan [30], China [31], and Mexico [32], which collectively reported that the TLR2 CC, TLR4 GG, and TLR9 AA genotypes were significantly associated with an increased likelihood of PTB. For example, Mhmoud et al. [11] in Sudan reported that the TLR2 T597C CC genotype was associated with a 2.5-fold increased risk of PTB, while Fouad et al. [24] in Egypt demonstrated that the TLR4 A896G GG genotype was significantly more prevalent among PTB patients. Similarly, Torres-García et al. [32] in Mexico found that the TLR9 G1174A AA genotype was associated with increased PTB susceptibility. These consistent findings across geographically and ethnically diverse populations suggest that these TLR polymorphisms may play a conserved role in modulating host susceptibility to MTB infection.

The increased risk associated with the TLR2 T597C polymorphism in our study could potentially be explained by its possible modulation of TLR2 expression and downstream signaling efficiency. It has been proposed that by altering post-transcriptional regulatory mechanisms and translation, this variant may lead to reduced TLR2 protein expression on macrophages and dendritic cells [33]. Such a cellular deficit could hinder the recognition of MTB components, potentially causing insufficient activation of the MyD88-IRAK-TRAF6 signaling pathway and a subsequent reduction in NF-κB activation [34]. Consequently, this might result in decreased production of pro-inflammatory cytokines essential for macrophage activation and granuloma formation, which could ultimately impair dendritic cell maturation, Th1 polarization, and the protective, interferon-gamma (IFN-γ) mediated response against intracellular bacilli [35]. Furthermore, it is plausible that weakened TLR2 signaling may compromise phagosome maturation and nitric oxide production, thereby potentially enabling MTB to evade clearance and persist intracellularly [36]. However, it is important to note that these mechanistic interpretations are extrapolated from studies of TLR2 signaling in general and have not been directly demonstrated for the rs3804099 variant in functional assays. Future studies incorporating gene expression analysis and functional immune assays are warranted to confirm these proposed mechanisms.

Similarly, our results may extend previous findings by suggesting that the TLR4 A896G polymorphism could favor PTB development by impairing TLR4 signaling, which might disturb the equilibrium between protective immunity and harmful inflammation in the lungs [24,37]. It has been hypothesized that this specific missense mutation may alter the extracellular domain of TLR4, potentially reducing its binding affinity to the MD-2/LPS complex and impairing receptor trafficking to the cell surface [37]. Such a signaling bottleneck through the MyD88 and TRIF pathways could diminish the production of crucial host-defense cytokines [38]. In carriers of this variant, it is possible that inadequate macrophage activation and defective phagosome maturation may facilitate intracellular MTB survival. This potential failure of early clearance might be compounded by impaired dendritic cell maturation and compromised Th1-type adaptive immune responses, potentially resulting in unchecked bacterial replication and progressive lung tissue damage [39]. As with TLR2, these proposed mechanisms require direct experimental validation to confirm the functional impact of the A896G variant on TLR4 signaling in the context of MTB infection.

Our observation of TLR9 G1174A may support the hypothesis that the polymorphism could influence PTB susceptibility by altering the transcription of this endosomal receptor, potentially leading to reduced expression levels and an impaired Th1-type cytokine response (specifically IFN-γ, IL-12, and TNF-α) upon exposure to unmethylated CpG motifs in bacterial DNA [17]. This putative transcription deficit might compromise downstream macrophage activation, potentially allowing MTB to establish infection. In certain populations, it has been suggested that the distribution of these TLR9 variants may be shaped by evolutionary selection pressures, where altered immune reactivity associated with the 1174G allele could significantly elevate the risk of transitioning from latent exposure to chronic active infection [18,31]. However, direct evidence linking the rs352139 variant to altered TLR9 transcription or function in the context of human tuberculosis remains limited, and our findings should be interpreted as hypothesis-generating rather than confirmatory.

In contrast to our findings, several studies have failed to demonstrate a significant association between the TLR2 T597C, TLR4 A896G, and TLR9 G1174A polymorphisms and PTB susceptibility. For instance, Wu et al. [40] found no significant association between TLR2 and TLR4 variants and TB risk in a Chinese cohort, while Kaushik et al. [22] did not observe a significant independent effect of TLR2, TLR4, or TLR9 variants on TB progression in an Indian HIV-co-infected population. Similarly, Biyikli et al. [41] reported no significant TLR2/TLR4 association in a Turkish cohort, and Davoodi et al. [42] found no significant TLR2 polymorphism association among Iranian TB patients. These discrepancies may be explained by several factors. First, allele and genotype frequencies for these SNPs vary considerably across ethnic groups due to differences in linkage disequilibrium patterns and population history, meaning a variant that is a strong risk marker in one population may be in low frequency or weak linkage in another [43]. Second, differences in study design—including sample size, matching criteria for controls (e.g., some studies did not match for HIV status or nutritional status, both of which we found to be independent predictors of PTB), and case definitions (bacteriologically confirmed vs. clinically diagnosed PTB)—can materially affect the ability to detect a true association. Third, gene-environment interactions, such as regional differences in M. tuberculosis lineage or in co-exposures like HIV prevalence and nutritional status, may modify the phenotypic effect of a given genotype, such that the same polymorphism confers different degrees of risk depending on background environmental pressures. Finally, publication bias favoring studies with positive findings cannot be excluded as a contributor to the heterogeneity observed across the literature [44].

An important contribution of this study was the combined multilocus genotype analysis of TLR2, TLR4, and TLR9 polymorphisms. Individuals harboring the triple-homozygous risk genotype profile (CC–GG–AA) demonstrated a significantly higher susceptibility to PTB (OR = 4.89; P < 0.001) compared to those carrying the baseline wild-type profile (TT–AA–GG) (Table 4). This finding indicates an association between this particular multilocus genotype pattern and PTB susceptibility in the study population. However, the multilocus analysis should be interpreted cautiously because the observed association does not demonstrate a dose-response relationship, cumulative genetic effect, or biological synergy among the three variants. Additionally, HIV co-infection was found to increase the odds of developing PTB by over three times, highlighting the immune impairment caused by HIV, particularly regarding CD4 + T-cells. Low BMI also emerged as an independent predictor of PTB, as undernourished individuals showed higher predisposition due to compromised immune responses (Table 5). These findings emphasize the role of genetic variants, HIV infection, and malnutrition in PTB risk, underscoring a significant public health challenge in regions like sub-Saharan Africa.

**Table 4:** Combined multilocus genotype patterns of TLR2, TLR4, and TLR9 polymorphisms among pulmonary tuberculosis patients and healthy controls.

Combined Genotype(TLR2–TLR4–TLR9)	PTB (n = 153)	Control (n = 153)	OR (95% CI)	P-value
CC–GG–AA	44 (28.7%)	18 (11.8%)	4.89 (2.38–10.05)	< 0.001*
CC–GG–GG	26 (17.0%)	20 (13.1%)	2.60 (1.20–5.65)	0.016*
CC–AA–AA	7 (4.5%)	8 (5.2%)	1.75 (0.56–5.45)	0.335
CC–AA–GG	15 (9.8%)	18 (11.8%)	1.67 (0.71–3.92)	0.242
TT–GG–AA	19 (12.4%)	17 (11.1%)	2.24 (0.97–5.13)	0.059
TT–GG–GG	9 (5.9%)	12 (7.8%)	1.50 (0.55–4.10)	0.430
TT–AA–AA	11 (7.2%)	16 (10.4%)	1.38 (0.55–3.46)	0.493
TT–AA–GG	22 (14.4%)	44 (28.7%)	Reference	

Note: ^*^P-value <0.05 is considered statistically significant.

**Table 5:** Multivariable logistic regression analysis of factors associated with pulmonary tuberculosis.

Variables	Adjusted OR (95% CI)	P-value
Sex	0.91 (0.56–1.49)	0.724
Age	1.01 (0.98–1.03)	0.517
BMI	0.79 (0.71–0.88)	< 0.001*
FBG	1.01 (0.99–1.02)	0.192
HIV co-infection	3.12 (1.48–6.58)	0.003*
Family history of PTB	1.35 (0.71–2.58)	0.364
Smoking habit	1.48 (0.52–4.22)	0.461
Alcohol intake	1.32 (0.81–2.15)	0.267
TLR2 CC genotype	3.74 (1.69–8.29)	0.001*
TLR4 GG genotype	2.97 (1.53–5.76)	0.001*
TLR9 AA genotype	1.94 (1.03–3.64)	0.039*

Note: ^*^P-value <0.05 is considered statistically significant.

This study has a few limitations. First, although genotyping was performed using validated PCR-RFLP methods with standardized quality-control procedures, the relatively modest sample size may have reduced the statistical power to detect weaker genetic associations and increased the uncertainty of effect estimates. Replication in larger, multicenter cohorts representing diverse Ethiopian ethnic groups is required to validate these findings. Second, only three TLR polymorphisms were investigated; future studies should evaluate additional innate immune gene variants and employ high-throughput genotyping or sequencing approaches to provide a more comprehensive assessment of host genetic susceptibility to pulmonary tuberculosis. Finally, functional studies investigating the biological effects of these polymorphisms on TLR expression and signaling are warranted to better understand the mechanisms underlying the observed associations. To the best of our knowledge, this study is the first to look at the relationship of PTB with TLR*4* A896G and TLR*9* G1174A and the second to look at TLR*2* T597C genetic variants with combined genotype analysis in the Ethiopian population. Even if the findings of this study serve as a baseline for these areas, further research is required to investigate other gene polymorphisms that could be reliable indicators of PTB in this population.

## 5. Conclusions

In conclusion, this study revealed a potential association of pulmonary tuberculosis with the *TLR2* T597C CC genotype and C allele, the TLR4 A896G GG genotype and G allele, and the TLR9 G1174A AA genotype and A allele, suggesting that the *TLR2* T597C, *TLR4* A896G, and *TLR9* G1174A gene polymorphisms may serve as biomarkers to identify PTB early in the Ethiopian population under study, but prospective studies are needed. Furthermore, the multi-locus genotype risk profile (CC-GG-AA), HIV co-infection, and low BMI were major determinants of disease risk. These results highlight the important role of innate immune genetic variability in tuberculosis pathogenesis and may contribute to the future development of genetic screening tools and personalized preventive strategies for high-risk populations. To fully comprehend the link between the *TLR2*, *TLR4*, and *TLR9* genes and PTB, more research with a large sample size and multi-ethnic Ethiopian cohorts is needed.

## Supporting information

S1 Fig Representative 2% agarose gel electrophoresis showing PCR-RFLP products of the TLR2 T597C (rs3804099) polymorphism. Lane M: 100-bp DNA ladder. Lanes 1, 2, and 8: homozygous TT genotype, showing a single undigested PCR product of 286 bp. Lanes 3, 4, and 9: heterozygous TC genotype, showing three fragments of 286 bp, 184 bp, and 102 bp. Lanes 5, 6, and 10: homozygous CC genotype, showing two restriction fragments of 184 bp and 102 bp. Lane 7: negative (no-template) control, confirming the absence of contamination during PCR amplification.
(PNG)

S2 Fig Representative 2% agarose gel electrophoresis showing PCR-RFLP products of the TLR4 A896G (rs4986790) polymorphism. Lane M: 100-bp DNA ladder. Lanes 8, 9, and 10: homozygous AA genotype, showing a single undigested PCR product of 249 bp. Lanes 1, 2, 3, and 4: heterozygous AG genotype, showing three fragments of 249 bp, 223 bp, and 26 bp. Lanes 5 and 6: homozygous GG genotype, showing two restriction fragments of 223 bp and 26 bp. Lane 7: negative (no-template) control, confirming the absence of contamination during PCR amplification.
(PNG)

S3 Fig Representative 2% agarose gel electrophoresis showing PCR-RFLP products of the TLR9 G1174A (rs352139) polymorphism. Lane M: 100-bp DNA ladder, labeled according to the manufacturer’s specifications. Lanes 5 and 6: homozygous GG genotype, showing a single undigested PCR product of 135 bp. Lanes 1, 2, 3, and 4: heterozygous GA genotype, showing three fragments of 135 bp, 108 bp, and 27 bp. Lanes 8, 9, and 10: homozygous AA genotype, showing two restriction fragments of 108 bp and 27 bp. Lane 7: negative (no-template) control, confirming the absence of contamination during PCR amplification. The reported 135-, 108-, and 27-bp values are expected experimental RFLP fragment sizes and are not molecular-weight ladder band sizes.
(PNG)

S1 Data Raw genotype and clinical dataset of the study participants (Excel file). Individual-level dataset of the 306 study participants (153 pulmonary tuberculosis [PTB] patients and 153 healthy controls) used for the analyses in this study. Each row represents one participant. Columns: Outcome (Case/Control); BMI (body mass index, kg/m²); FBG (fasting blood glucose, mg/dl); HIV (HIV status, Yes/No); Fam Hist (family history of PTB, Yes/No); Smoking (Yes/No); Alcohol (alcohol intake, Yes/No); and the genotypes of TLR2 T597C (TT/TC/CC), TLR4 A896G (AA/AG/GG), and TLR9 G1174A (GG/GA/AA).
(XLSX)

## Abbreviations

BMI: Body Mass Index
CI: Confidence Interval
DNA: Deoxyribonucleic Acid
FBG: Fasting Blood Glucose
MTB/M.tb: Mycobacterium Tuberculosis
OR: Odds Ratio
PCR: Polymerase Chain Reaction
PTB: Pulmonary Tuberculosis
TLR: Toll-Like Receptor
TB: Tuberculosis
