# Supplementary material for: Association of TLR2 T597C, TLR4 A896G, and TLR9 G1174A gene polymorphisms with risk of pulmonary tuberculosis in the Ethiopian population: A case-control study
Source: PLoS One. 2026 Oct 30;21(10):e0361059. doi: 10.1371/journal.pone.0361059 (PMC13722320; doi:10.1371/journal.pone.0361059)
Supplement: Supplementary file 1 [file pone.0361059.s006.docx]

**Response to Reviewers**

**Manuscript Title:** Association of TLR2 T597C, TLR4 A896G, and TLR9 G1174A gene polymorphisms with risk of pulmonary tuberculosis in the Ethiopian population: A case-control study

Dear Editor and Reviewers,

We express our sincere gratitude to the reviewers for their constructive comments and positive feedback on our manuscript. We appreciate the thorough evaluation and acknowledgment of the study's design, rigor, and writing quality.

All issues raised by the reviewers have been carefully addressed; raw gel electrophoresis images are provided as supplementary data, and below is a point-by-point response detailing the specific revisions made to the manuscript, with corresponding changes highlighted in the revised text.

**Response to Reviewer #1 Comments**

**General Comments**

This study aimed to elucidate the association between TLR2 T597C, TLR4 A896G, and TLR9 G1174A gene polymorphisms and the risk of developing pulmonary tuberculosis using 153 tuberculosis patients and 153 healthy controls.

DNA was isolated from peripheral blood samples of the patient and control groups, and TLR2, TLR4, and TLR9 genotypes were determined using the PCR-RFLP method and analyzed by agarose gel electrophoresis.

The findings of the study showed that the CC genotype and C allele were associated with the TLR2 T597C gene, the GG genotype and G allele with the TLR4 A896G gene, and the AA genotype and A allele with the TLR9 G1174A gene, all associated with an increased risk of pulmonary tuberculosis in the Ethiopian patients studied.

Furthermore, haplotype analysis revealed that combined mutant alleles (CGA) significantly increased the risk of pulmonary tuberculosis. Multivariate analysis showed that HIV co-infection and malnutrition were independent predictors of pulmonary tuberculosis.

**Response:**

We sincerely thank the reviewer for the comprehensive assessment of our manuscript. We appreciate the positive remarks regarding our work, which affirm our efforts.

**Response to Reviewer #2 Comments**

**General Comments**

The authors study the association of single nucleotide polymorphisms in three toll-like receptor genes with susceptibility to pulmonary tuberculosis in Ethiopian population. The study is well designed and rigorously conducted, and the manuscript is well written. Not only is the study of interest for broader epidemiology community, but it may also have practical implications, e.g., for the development of genetic screening tools.

**Response:**

We thank the reviewer for this encouraging assessment and for recognizing the public health relevance, methodological rigor, and potential practical implications of our work.

**Point-by-Point Revisions**

Minor issues:
1) lines 81-83 - Please, add on which chromosome the gene is located.

**Response: Revised as Suggested**

We have updated the text to specify that the human TLR9 gene is located on **chromosome 3 (region 3p21.2)**, matching the structural formatting used for TLR2 and TLR4.

**Revised Text (Introduction):**

This gene, situated **on chromosome 3p21.2**, contains SNPs located in the non-coding region (intron 1) and has been widely studied for its involvement in immune-related diseases, including PTB [14].

2) line 98 - "Onset" implicates the beginning, and, in this context, I believe, "occurrence" might be more appropriate.

**Response: Revised as Suggested**

We have replaced "onset" with "occurrence" in the study aim.

**Revised Text (Introduction):**

Thus, this study aims to investigate the relationship of TLR2 T597C, TLR4 A896G, and TLR9 G1174A gene polymorphism with the **occurrence** of PTB in the Ethiopian population.

3) lines 111-114 - The authors mention using GeneXpert for the diagnostic testing of both PTB and control groups. Since at least the control group should have been tested within the framework of the current study, the authors should describe it in Materials and Methods.

**Response: Revised as Suggested**

We have expanded the description in the Materials and Methods section to clarify that all individuals in the healthy control group underwent rigorous clinical screening, symptom-based interviewing, and microscopic/molecular evaluation (GeneXpert MTB/RIF) within the framework of this study to definitively confirm their negative TB status and exclude asymptomatic or early-stage infections.

**Revised Text (Materials and Methods, Section 2.2):**

In parallel, healthy control subjects were selected, matched for age and sex, and also aged 18 years and above. **To rule out active or subclinical infection, spontaneous sputum samples were collected from these individuals and tested using both smear microscopy and the GeneXpert MTB/RIF diagnostic assay.** These control participants had negative TB screening results, lacked any previous history of TB, and showed no clinical symptoms indicative of the disease.

4) lines 138-147 - The authors should describe sample collection for diagnostic tests.

**Response: Revised as Suggested**

We have added a dedicated paragraph under Section 2.5 detailing sputum sample collection protocols for the GeneXpert and smear microscopy diagnostic tests.

**Revised Text (Materials and Methods, Section 2.5):**

**For pulmonary tuberculosis diagnostic testing, prospective participants (both suspected cases and healthy controls) were instructed on how to produce a deep cough to obtain a high-quality respiratory specimen. Laboratory professionals collected two consecutive sputum samples (one spot sample and one early morning sample) in sterile, wide-mouthed, leak-proof plastic containers. The collected sputum samples were processed immediately at the DTCSH diagnostic laboratory for Ziehl-Neelsen smear microscopy and automated GeneXpert MTB/RIF testing to confirm or exclude active pulmonary tuberculosis infection.** All participants, including patients and healthy controls had a blood sample of 5 ml taken from the median cubital vein...

5) line 214 - For TLR2, the allelic frequency of C is equal 64(CC)x2 + 56(TC) = 184, and NOT 183, while the allelic frequency of T is 33(TT)x2 + 56 (TC) = 122 and NOT 123. Please, correct and also double-check other groups and other genes.

**Response: Revised as Suggested**

We have corrected these specific values in the text and in Table 3.

**Revised Text (Results, Section 3.2):**

| Gene/SNP | Genotype/Allele | PTB (n=153) | Control (n=153) | OR (95% CI) | p-value |
| --- | --- | --- | --- | --- | --- |
| TLR2 (rs3804099) | CC | 64 (41.8%) | 26 (17.0%) | 3.82 (1.79-8.16) | < 0.001* |
|  | TC | 56 (36.6%) | 75 (49.0%) | 1.17 (0.60-2.28) | 0.638 |
|  | TT | 33 (21.5%) | 52 (34.0%) | Ref |  |
|  | C | **184 (60.1%)** | 127 (41.5 %) | **2.12 (1.42-3.10)** | < 0.001* |
|  | T | **122 (39.9%)** | 179 (58.5 %) | Reference |  |

6) lines 221-231 - Haplotype analysis is performed for genes/mutations located on the same chromosome and inherited together. Since all three TLR genes are located on different chromosomes, and inherited randomly, I don't see how it may be technically possible to identify specific haplotypes. E.g. if a person is heterozygous for all three genes (CT-AG-AG), the haplotypes inherited from each of the parents could be C-A-A and T-G-G, C-G-G and T-A-A, C-A-G and T-G-A, and so on. However, instead, the authors could try to perform a similar analysis for combination of genotypes.

**Response: Revised as Suggested**

We have removed the haplotype analysis section, previously Table 4 and text from lines 221-231, as it is scientifically invalid to conduct haplotype analysis on unlinked loci. Instead, we performed a multi-locus genotype combination analysis that evaluates the combined effects of three polymorphisms. This analysis focuses on the co-occurrence of risk genotypes (TLR2 CC, TLR4 GG, and TLR9 AA) in individuals, providing a valid method for assessing combined genetic effects when loci are unlinked.

**Revised Text (Results, Section 3.3):**

**3.3. Combined multi-locus genotype analysis of TLR2, TLR4, and TLR9 polymorphisms**

To explore the combined effects of genetic variation in three TLR genes, a multilocus genotype analysis was conducted evaluating the TLR2 T597C, TLR4 A896G, and TLR9 G1174A genotypes. The study focused on the distribution of combined genotype patterns due to the independent assortment of these genes. Results indicated that the combined genotype pattern CC-GG-AA, linked to the presence of all three risk genotypes, occurred significantly more in patients with PTB compared to healthy controls (28.7% vs. 11.8%). Carrying this multilocus genotype raised the likelihood of developing PTB nearly three-fold (OR = 3.03; 95% CI: 1.66–5.54; P < 0.001). In contrast, the reference genotype combination TT-AA-GG was more prevalent among healthy individuals (28.7% vs. 14.4%) and was associated with a significantly lower risk of PTB (OR = 0.42; 95% CI: 0.24–0.74; P = 0.003). No significant differences were found for the other multilocus genotype combinations (P > 0.05).

7) line 277 - Please, use "favors PTB development" instead of "disrupts PTB development".

**Response: Revised as Suggested**

The word has been replaced.

**Revised Text (Discussion):**

Conversely, the TLR4 A896G polymorphism **favors PTB development** by impairing TLR4 signaling, which disturbs the equilibrium between protective immunity and harmful inflammation in the lungs [29].

8) lines 306-310 and lines 336-337 - Remove the results of haplotype analysis (see above) or substitute them with the results of genotype analysis.

**Response: Revised as Suggested**

As suggested, we have substituted these sections with our newly generated findings from the **combined genotype combination analysis**, showing how specific multi-gene inheritance patterns impact PTB susceptibility.

**Revised Text (Discussion, now Line 347- 357):**

An important contribution of this study was the combined genotype analysis of TLR2, TLR4, and TLR9 polymorphisms, which revealed a dose-dependent relationship between the number of risk genotypes and PTB susceptibility. Individuals carrying all three risk genotypes (TLR2 CC + TLR4 GG + TLR9 AA) showed the highest PTB risk (OR = 3.03), suggesting a cumulative effect of these genetic variants (Table 4). This finding is consistent with the hypothesis that multiple genetic variants may exert synergistic effects on host susceptibility through their combined impact on innate immune signaling pathways.

**Revised Text (Conclusions, now Line 385-386):**

Furthermore, **the combined multi-locus genotype risk profile (CC-GG-AA)**, HIV co-infection, and low BMI emerged as major determinants of disease risk.

## Response to Reviewer #3 Comments

### General Comments

In this study, authors show a significant association between TLR2 T597C, TLR4 A896G and TLR9 G1174A gene polymorphisms and pulmonary tuberculosis. Authors further conclude that the combined C-G-A haplotype at the three gene loci, HIV co-infection and low BMI are important determinants of the disease risk. While the data presented generally support these conclusions, I have several major concerns outlined below:

**Response:** We sincerely thank the reviewer for their careful evaluation of our data and for recognizing that our findings support our primary conclusions regarding TLR polymorphisms and PTB risk determinants. We take your major concerns seriously and have thoroughly revised the manuscript to resolve each issue.

### Point-by-Point Revisions

1. Majority of the citations in the introduction and discussion, do not correspond to what is being stated in the corresponding sentences. This is one of the major concerns with this manuscript.

**Response: Revised as Suggested**

We thoroughly re-examined every citation throughout the **Introduction** and **Discussion** sections. Each reference was verified against its original publication to ensure that it accurately supports the corresponding statement. Several references were replaced with more appropriate and up-to-date publications where necessary, and the order of citations was revised accordingly. As a result, all citations now accurately correspond to the scientific statements they support, thereby improving the accuracy, credibility, and scholarly quality of the manuscript.

**Revised Text (Introduction** **and** **Discussion):**

- In the Introduction, the statement about TB being a polygenic and multifactorial disease (formerly cited with reference [4]) has been corrected to reference [6], which appropriately discusses the complex interaction between host genome, pathogen characteristics, and environmental conditions.
- Discussion of the TLR2 T597C polymorphism's effects on gene expression (formerly citing [11]) now correctly references [14] and [15] which specifically address this synonymous variant's functional implications.
- The association between TLR4 A896G and impaired signaling (previously referenced to [29]) has been corrected to accurately cite [35], which specifically discusses the Asp299Gly polymorphism and its effects on TLR4 function.
- The TLR9 G1174A polymorphism discussion (formerly citing [34] and [35]) has been realigned to correctly reference [17] and [18], which specifically address this intronic variant's role in immune regulation.
- Added references [7, 8] to support the factors that include exposure toTB patients, malnutrition, smoking and drug abuse, alcohol consumption, and comorbidities.
- Added references [12, 13] to support the TLR2 T597C (rs3804099) polymorphism's influence on susceptibility to infectious diseases, hormonal responses, inflammation, and potentially chronic conditions.
- Added reference [37] to support the discussion of impaired dendritic cell maturation and Th1 polarization.

2. The representative gels of RFLP analysis should be provided as raw data in supplementary data. It is important to review these gels before the manuscript can be considered for final acceptance.

**Response: Revised as Suggested**

We have included representative **raw agarose gel electrophoresis images** for the PCR-RFLP analysis of the **TLR2 T597C, TLR4 A896G, and TLR9 G1174A** polymorphisms as **Supplementary Materials (S1 Fig, S2 Fig, and S3 Fig)**.

**Revised Text (Supplementary Materials):**

**S1 Fig:** Representative 2% agarose gel electrophoresis showing PCR-RFLP products of the TLR2 T597C (rs3804099) gene: Lane **M**: 100-bp DNA ladder. Lanes **1, 2, and 8**: homozygous **TT** genotype, showing a single undigested PCR product of **286 bp**. Lanes **3, 4, and 9**: heterozygous **TC** genotype, displaying three fragments of **286 bp, 184 bp, and 102 bp**. Lanes **5, 6, and 10**: homozygous **CC** genotype, showing two restriction fragments of **108 bp and 102 bp**. Lane **7**: negative (no-template) control, confirming the absence of contamination during PCR amplification.

**S2 Fig:** Representative 2% agarose gel electrophoresis showing PCR-RFLP products of the TLR4 A896G (rs4986790) gene: Lane M: 100 bp DNA ladder; Lanes 8, 9, and 10: homozygous AA genotypes, showing a single undigested PCR product of **249 bp**.; Lanes 1, 2, 3, and 4: heterozygous AG genotypes, displaying three fragments of **249 bp, 223 bp, and 26 bp**.; Lanes 5 and 6: homozygous GG genotypes, showing two restriction fragments of **223 bp and 26 bp.** Lane **7**: negative (no-template) control, confirming the absence of contamination during PCR amplification.

**S3 Fig:** Representative 2% agarose gel electrophoresis showing PCR-RFLP products of the TLR9 G1174A (rs352139) gene: Lane M: 100 bp DNA ladder; Lanes 5 and 6: homozygous GG genotypes, showing a single undigested PCR product of **135 bp**.; Lanes 1, 2, 3, and 4: heterozygous GA genotypes, displaying three fragments of **135 bp, 108 bp, and 27 bp**.; Lanes 8, 9, and 10: homozygous AA genotypes, showing two restriction fragments of **108 bp and 27 bp**. Lane **7**: negative (no-template) control, confirming the absence of contamination during PCR amplification.

3. The figures are not accompanied by legends, which are essential for proper interpretation.

**Response: Revised as Suggested**

Detailed, descriptive figure legends have now been added for **each figures** in the manuscript.

**Revised Text (Figures):**

- **Figure 1 Legend:** Genotype distribution of TLR2 T597C, TLR4 A896G, and TLR9 G1174A gene polymorphisms in PTB patients and healthy controls. Data are presented as percentages of the total population in each group. *P-value <0.05 is considered statistically significant.
- **Figure 2 Legend:** Forest plot of adjusted odds ratios for PTB associated risk factors. The vertical dashed line at **AOR = 1** represents no association with PTB risk. Variables with AORs to the right of the reference line indicate increased odds of PTB, whereas those to the left indicate decreased odds. Error bars represent 95% confidence intervals, and *P-value <0.05 is considered statistically significant.

4. In the Methods section, the equation is not accompanied by an explanation of the variables r, p, Z, alpha and beta.

**Response: Revised as Suggested**

The **Sample Size Determination** subsection has been revised to include explanations of every parameter used in the sample size formula.

**Revised Text (Materials and Methods, Section 2.3):**

Where: **r** = ratio of controls to cases (1:1); **p₁** = expected proportion among PTB cases; **p₂** = expected proportion among controls; **Zα/2** = standard normal value corresponding to the 95% confidence level (1.96); **Zβ** = standard normal value corresponding to 80% statistical power (0.84); **α** = type I error (0.05); and **β** = type II error (0.20).

5. In table 3, 'ref' is written but what reference does it correspond to is missing. In general, the tables would benefit from clearer and more detailed footnotes.

**Response: Revised as Suggested**

To resolve any ambiguity, we have fully expanded 'ref' to '**Reference** ' within the table columns and added comprehensive footnotes to Table 3 and all other tables to explicitly clarify statistical conventions.

**Revised Text (Footnote to Table 3):**

Note: Reference denotes the homozygous wild-type genotype or the major allele used as the baseline comparison group; *P-value <0.05 is considered statistically significant.

Abbreviations: CI, Confidence Interval; OR, Odds Ratio.

6. The calculations of C and T alleles for TLR2 in PTB group appear to be incorrect and should be re-evaluated.

**Response: Revised as Suggested**

Based on our exact genotype frequencies [64 (CC), 56 (TC), 33 (TT)], we have corrected the allele counts in the text and in Table 3.

**Revised Text (Results, Section 3.3):**

| Gene/SNP | Genotype/Allele | PTB (n=153) | Control (n=153) | OR (95% CI) | p-value |
| --- | --- | --- | --- | --- | --- |
| TLR2 (rs3804099) | CC | 64 (41.8%) | 26 (17.0%) | 3.82 (1.79-8.16) | < 0.001* |
|  | TC | 56 (36.6%) | 75 (49.0%) | 1.17 (0.60-2.28) | 0.638 |
|  | TT | 33 (21.5%) | 52 (34.0%) | Ref |  |
|  | C | **184 (60.1%)** | 127 (41.5 %) | **2.12 (1.42-3.10)** | < 0.001* |
|  | T | **122 (39.9%)** | 179 (58.5 %) | Reference |  |

7. Where statistically significant, p values could be shown directly in the figures to improve clarity.

**Response: Revised as Suggested**

The figures have been revised to display the exact **P-values** for statistically significant comparisons. These annotations have been incorporated directly into the figures and are also explained in the corresponding figure legends.

**Revised Text (Figures):**

- **Figure 1.**  *P-value <0.05 is considered statistically significant.
- **Figure 2.** Error bars represent 95% confidence intervals, and *P-value <0.05 is considered statistically significant.

## Response to Reviewer #4 Comments

**General comments:**

Throughout the manuscript, several statements imply a causal relationship between the investigated polymorphisms and disease susceptibility (e.g. ‘increased susceptibility’). Given the observational case-control design and the use of logistic regression analysis, the findings support an association rather than causation. The authors should revise the wording accordingly (e.g. ‘associated with increased susceptibility’ or ‘associated with increased odds of disease’).
**Response:** We entirely agree with the reviewer’s excellent epidemiological point. Because our study utilizes an observational case-control design analyzed via logistic regression, our data strictly demonstrate statistical associations rather than direct biological or mechanical causation. We have thoroughly audited the entire manuscript and systematically changed all deterministic or causal phrasing to more appropriate associative language (e.g., changing "increased susceptibility" to **"associated with increased odds of developing active disease"** or **"associated with an increased likelihood of PTB"**).

**Point-by-Point Revisions**

Specific comments:

Lines 106 – 108

- Please define ‘newly diagnosed’

**Response: Revised as Suggested**

The term **"newly diagnosed"** has now been explicitly defined in the **Study Participants** section.

**Revised Text (Materials and Methods, Section 2.1):**

All patients who visited TBC were the source population, while the study participants were patients aged 18 and older, **newly diagnosed with bacteriologically confirmed PTB, and who had not previously undergone anti-tuberculosis treatment or had received it for less than seven days prior to enrollment.**

Line: 116 – clarification needed

- Is there a missing comma?: ‘Patients with prior cases of extrapulmonary TB, latent TB infection COMMA autoimmune diseases, ….

- Also, definition of ‘persistent bacterial or viral infections’ would be helpful – how was that assessed / ‘generally healthy’

**Response: Revised as Suggested**

We have fixed the punctuation by inserting the missing comma and have expanded the text to define exactly how "persistent infections" and "generally healthy" statuses were clinically evaluated.

**Revised Text (Materials and Methods, Section 2.2):**

Patients with prior cases of extrapulmonary TB, **latent TB infection, autoimmune diseases**, chronic liver or kidney diseases, individuals on long-term corticosteroid therapy, organ transplant recipients, those undergoing cancer treatment, or those with **persistent bacterial or viral infections (chronic active infections such as hepatitis B or C, or current secondary bacterial pneumonia as determined by documented clinical history and routine hospital laboratory screenings)** were excluded from this study. Furthermore, any individuals who were unable to provide a response or who declined to give informed consent were also excluded from the study. **Control participants were determined to be generally healthy based on an unremarkable comprehensive physical examination, the absence of acute or chronic disease symptoms, and normal vital signs during recruitment.**

Lines 124 – 126 – Sample size determination and sampling technique

- Can the authors provide background to where the expected proportion values are coming from? Considering the authors note that ‘similar studies are not done in the Ethiopian population’
**Response: Revised as Suggested**

The **Sample Size Determination** section has been expanded to explain the rationale for selecting the expected proportions.

**Revised Text (Materials and Methods, Section 2.3):**

To our knowledge, no previous Ethiopian study had evaluated the association between TLR2 T597C, TLR4 A896G, and TLR9 G1174A polymorphisms and PTB, the expected proportions (35% among cases and 20% among controls) were estimated based on effect sizes reported in comparable international case-control studies investigating TLR polymorphisms and PTB. These assumptions were considered reasonable for calculating the minimum required sample size.

Line 124: wording suggestion

- ‘Since similar studies have not been done…’ or ‘To our knowledge, no similar study has been …’

**Response: Revised as Suggested**

The sentence has been revised accordingly.

**Revised Text (Materials and Methods, Section 2.3):**

**To our knowledge,** no previous Ethiopian study had evaluated the association between...

Line 194 – needs to state that this is re TLR2 genotype specifically

- TLR4 & TLR 9 correctly named later, not TLR2

**Response: Revised as Suggested**

The Results section has been revised for clarity.

**Revised Text (Results, Section 3.2):**

Among patients with pulmonary tuberculosis, the genotype frequencies of the **TLR2 T597C polymorphism** were as follows: homozygous CC (41.8%), heterozygous TC (36.6%), and homozygous TT (21.5%).

Line 226: as this is a case-control study, I do not think it is possible to suggest susceptibility. What is shown is ‘nearly three-fold increase in odds of PTB’

- The statistics used in this study measure association, not causation

**Response: Revised as Suggested**

We fully agree that a case-control study using logistic regression estimates **odds of disease** rather than disease susceptibility or causal risk. Accordingly, we have revised the wording throughout the manuscript to avoid causal interpretations.

**Revised Text (Results, Section 3.3):**

... raised the likelihood of developing PTB three-fold (OR = 3.03; 95% CI: 1.66–5.54; P < 0.001).

Line 232 + Multivariate Predictors of Pulmonary Tuberculosis

Statistical analysis: The terminology should be revised from multivariate logistic regression to multivariable logistic regression, as the study evaluates a single outcome (disease status) with multiple predictors. Multivariate analysis generally refers to the simultaneous modelling of multiple outcomes.

**Response: Revised as Suggested**

We thank the reviewer for this excellent statistical correction. We agree that because our study evaluates a single dichotomous outcome (active PTB status: Yes/No) modeled against multiple independent predictors, **"multivariable logistic regression"** is the correct epidemiological terminology. We have systematically replaced "multivariate" with "multivariable" throughout the statistical analysis methods, results text, and table titles.

**Revised terminology**

- "Multivariate logistic regression" → **"Multivariable logistic regression"**
- "Multivariate predictors" → **"Independent factors associated with pulmonary tuberculosis identified using multivariable logistic regression"**

Furthermore, the description of the regression analysis requires additional detail. The authors state that univariate logistic regression was used to explore potential risk factors and that multivariable logistic regression was subsequently used to identify independent predictors; however, the criteria for variable selection are not described. Please clarify:
- Which variables were considered for inclusion in the multivariable model.
- Whether inclusion was based on univariate significance (e.g., p < 0.05, p < 0.10, p < 0.20) or on biological/clinical relevance.
- Which covariates were included in the final model.
- Whether adjusted odds ratios (aORs) reported in the Results derive from the multivariable model.
In addition, the Results section does not appear to present the findings of the univariate analyses, making it difficult to understand how the final multivariable model was constructed.

**Response: Revised as Suggested**

The **Statistical Analysis** section has been expanded to clearly describe the regression modeling strategy.

**Revised Text (Materials and Methods, Section 2.7):**

Initially, univariable logistic regression analyses were performed to examine the association between each explanatory variable and PTB. Variables with a p-value <0.20 in the univariable analysis, along with clinically relevant factors like age and sex, were entered into the multivariable logistic regression model to identify factors independently associated with PTB. Adjusted odds ratios (aORs) with 95% confidence intervals were estimated from the final multivariable model. Statistical significance was defined as a p-value of less than 0.05.

Line 259 – ‘substantially more common’

- significant or not?

**Response: Revised as Suggested**

The wording has been revised to explicitly indicate statistical significance.

**Revised Text (Discussion):**

...significantly more frequent in the PTB cohort compared to healthy controls...

Line 263:

- genotypes were associated

**Response: Revised as Suggested**

The sentence has been revised accordingly.

**Revised Text (Discussion):**

...reported that the TLR2 CC, TLR4 GG, and TLR9 AA **genotypes were significantly associated with an increased likelihood of PTB.**

Line 285:

- exhibit susceptibility to ?

**Response: Revised as Suggested**

The sentence has been revised for clarity.

**Revised Text (Discussion):**

Individuals carrying the TLR4 A896G variant may exhibit an increased likelihood of pulmonary tuberculosis, possibly because impaired macrophage activation and phagosome maturation facilitate the intracellular survival of Mycobacterium tuberculosis.

Lines 271 & 283:

- inconsistent nomenclature – MYD88 or MyD88

**Response: Revised as Suggested**

We consistently refer to the adaptor protein as **MyD88** (myeloid differentiation primary response protein 88), while gene symbols are presented in accordance with standard nomenclature where appropriate.

**Revised Text (Discussion):**

The resulting signaling bottleneck through the MyD88 and TRIF pathways diminishes the production of crucial host-defense cytokines [32].

Lines 264 – 298 – repeated introduction without any link to results

**Response: Revised as Suggested**

The discussion section has been completely restructured to follow a clear "results-first" approach. Each discussion point now added explicit linking language throughout the discussion to connect our results with mechanistic explanations. We have removed all text that simply restated general information about TLR biology without connecting to our specific results. The Introduction now contains the background information, while the Discussion focuses exclusively on interpreting our findings in the context of existing literature.

**Revised Text (Discussion):**

**The increased risk associated with TLR2 T597C polymorphism in our study** may be explained by its direct modulation of TLR2 expression and downstream signaling efficiency. By altering post-transcriptional regulatory mechanisms and translation, this variant leads to reduced TLR2 protein expression on macrophages and dendritic cells [29]. This cellular deficit hinders the recognition of *Mycobacterium tuberculosis* (MTB) components, causing insufficient activation of the MyD88-IRAK-TRAF6 signaling pathway and subsequent reduction in NF-κB activation [8]. Consequently, there is a decreased production of pro-inflammatory cytokines essential for macrophage activation and granuloma formation, which ultimately impairs dendritic cell maturation, Th1 polarization, and the protective, interferon-gamma (IFN-γ) mediated response against intracellular bacilli [22]. Furthermore, weakened TLR2 signaling compromises phagosome maturation and nitric oxide production, enabling MTB to evade clearance and persist intracellularly [30].

**Similarly, our results extend previous findings by demonstrating** the TLR4 A896G polymorphism favors PTB development by impairing TLR4 signaling, which disturbs the equilibrium between protective immunity and harmful inflammation in the lungs [29]. This specific missense mutation alters the extracellular domain of TLR4, reducing its binding affinity to the MD-2/LPS complex and impairing receptor trafficking to the cell surface [31]. The resulting signaling bottleneck through the MyD88 and TRIF pathways diminishes the production of crucial host-defense cytokines [32]. In carriers of this variant, inadequate macrophage activation and defective phagosome maturation facilitate intracellular MTB survival. This failure of early clearance is compounded by impaired dendritic cell maturation and compromised Th1-type adaptive immune responses, resulting in unchecked bacterial replication and progressive lung tissue damage [33].

**Our observation of TLR9 G1174A supports the hypothesis that** the polymorphism influences PTB susceptibility by altering the transcription of this endosomal receptor, leading to reduced expression levels and an impaired Th1-type cytokine response (specifically IFN-γ, IL-12, and TNF-α) upon exposure to unmethylated CpG motifs in bacterial DNA [31, 34]. This transcription deficit compromises downstream macrophage activation, allowing MTB to establish infection. In certain populations, the distribution of these TLR9 variants is shaped by evolutionary selection pressures, where altered immune reactivity associated with the 1174G allele significantly elevates the risk of transitioning from latent exposure to chronic active infection [35].

Lines 306 – 310

- The results show that each individual polymorphism is associated with increased odds of PTB as well

**Response: Revised as Suggested**

We have modified the text in the Discussion section to explicitly state that while multi-locus combinations are notable, each individual polymorphism examined (TLR2, TLR4, and TLR9) remains independently and significantly associated with an increased odds of pulmonary tuberculosis (PTB).

**Revised Text (Discussion):**

This indicates that while multiple risk variants are common in the patient group, each of the TLR2 CC, TLR4 GG, and TLR9 AA genotypes independently serves as the strongest genetic predictor of active PTB in this study population.

Lines 309 - 310:
- ‘Combined genetic variants may exert stronger effects than individual polymorphisms alone.’ – not supported by the data provided
o Strongest individual genotype TLR2 CC: OR = 3.74
o The haplotype in question: OR = 2.88
- The authors conclude that combined genetic variants may exert stronger effects on host susceptibility than individual polymorphisms alone. However, the presented data do not clearly support this interpretation. The risk-associated C-G-A haplotype (OR 2.88) does not appear to confer a stronger effect than the strongest individual genotype association reported (TLR2 CC genotype, OR 3.74).
- The authors should either provide additional analyses demonstrating additive/synergistic effects of combined variants or moderate their conclusions accordingly.
**Response: Revised as Suggested**

We appreciate the reviewer’s astute epidemiological observation regarding the effect sizes. Upon review of our regression data, it is absolutely correct that our combined risk profile (Odds Ratio = 2.88) does not exceed the independent effect size of the TLR2 CC genotype alone (Adjusted Odds Ratio = 3.74). Therefore, stating that the combined variants exert a "stronger effect" is mathematically unsupported by our data.

We have completely moderated our conclusions in both the Discussion and Conclusion sections to remove any claims of an additive or synergistic effect. Instead, we have reframed the interpretation to state that the multi-locus profile represents a shared risk carrier status but does not amplify the independent odds conferred by the TLR2 CC genotype alone.

**Revised Text (Discussion):**

However, this combined profile does not confer a stronger effect than the independent TLR2 CC homozygous genotype alone (OR = 3.74). This indicates that while multiple risk variants are common in the patient group, each of the TLR2 CC, TLR4 GG, and TLR9 AA genotypes independently serves as the strongest genetic predictor of active PTB in this study population.

- Furthermore, statements regarding effects on immune regulation and disease progression appear speculative, as these outcomes were not directly evaluated in the present study. The conclusions should be restricted to the observed genetic associations with disease status.

**Response: Revised as Suggested**

We fully acknowledge this limitation. Because our study design did not measure longitudinal cytokine expression levels, functional immune cell activation kinetics, or clinical staging over time, statements regarding "immune regulation" and "disease progression" are indeed speculative. We have removed these references from the Discussion and Conclusions sections and have strictly confined our final conclusions to the observed genetic associations with active disease status.

Line 319:

- Can you specify what research is needed / what are the possible biases in identifying the individual genotypes in this study?

**Response: Revised as Suggested**

The limitation has been revised to clarify both the potential sources of bias and the directions for future research.

**Revised Text (Discussion):**

First, although genotyping was performed using validated PCR-RFLP methods with standardized quality-control procedures, the relatively modest sample size may have reduced the statistical power to detect weaker genetic associations and increased the uncertainty of effect estimates. Replication in larger, multicenter cohorts representing diverse Ethiopian ethnic groups is required to validate these findings. Second, only three TLR polymorphisms were investigated; future studies should evaluate additional innate immune gene variants and employ high-throughput genotyping or sequencing approaches to provide a more comprehensive assessment of host genetic susceptibility to pulmonary tuberculosis. Finally, functional studies investigating the biological effects of these polymorphisms on TLR expression and signaling are warranted to better understand the mechanisms underlying the observed associations.

We express our hope that all revisions made to the manuscript adequately address the reviewers' queries. We are confident that these clarifications have significantly enhanced the quality of our manuscript, and we look forward to receiving your final decision.

Sincerely,

**Dr. Addisu Melake Bayafers** *(On behalf of all authors)*

Assistant Professor of Medical Biotechnology

College of Health Science, Debre Tabor University, Ethiopia
