# Supplementary material for: Association of TLR2 T597C, TLR4 A896G, and TLR9 G1174A gene polymorphisms with risk of pulmonary tuberculosis in the Ethiopian population: A case-control study
Source: PLoS One. 2026 Oct 30;21(10):e0361059. doi: 10.1371/journal.pone.0361059 (PMC13722320; doi:10.1371/journal.pone.0361059)
Supplement: Supplementary file 2 [file pone.0361059.s007.docx]

**Response to Editor and Reviewers**

We would like to thank the editor and reviewers for taking the time to review our article. We have revised the manuscript in accordance with your suggestion.

**Response to the Editor**

**Additional Editor Comments:** Although the authors have addressed some of the reviewers' comments adequately, there are some outstanding issues that need to be addressed. The manuscript contains several citation inaccuracies, which suggests that the authors may not have carefully reviewed the referenced literature. This level of inconsistency raises concerns about the manuscript preparation.

**Author’s Response:** We apologize unreservedly for the citation inconsistencies present in the previous submission. The errors arose from reference index shifting during automated reference manager re-sorting across multiple revision drafts. To completely resolve this issue:

- We performed a strict 100% manual audit of every reference in the reference list and corrected incomplete references.
- We verified that every single numeric callout in the body text [1] through [44] points precisely to the intended primary study.

**Editor Comment:** If the authors have used AI to generate any of the sections of this manuscript, that should be explicitly disclosed, and the authors should take full responsibility for the integrity and reliability of information generated by AI.

**Author’s Response:** We acknowledge the importance of transparency regarding generative AI tools in academic publishing. AI technology (specifically Large Language Models) was used solely as a writing assistant for copyediting, grammar refinement, and formatting language during manuscript preparation. AI was not used to perform statistical calculations, interpret genomic data, or create reference metadata. The authors have manually verified every citation, data point, and scientific statement, and take full, sole responsibility for the accuracy, reliability, and scientific integrity of the entire manuscript. We have added an explicit Declarations / **AI Disclosure Statement** to the back matter of the manuscript:

**Editor Comment:** Similarly, as mentioned by the reviewer, details of the primer sequences also need to be clarified.

**Author’s Response:** In accordance with the Editor's and Reviewers' directions, we have updated Section 2.6 ("TLR2 T597C, TLR4 A896G, and TLR9 G1174A gene analysis") and Table 1 in the main text to explicitly document the exact genomic locations, annotated region, and primer sequences according to the human reference genome (GRCh38).

**Response to the Reviewers**

**Response to Reviewer #2**

Reviewer #2: The authors answered all of my previous comments and significantly improved the manuscript. Unfortunately, not all the answers contained sufficient details, or were scientifically correct.

First and foremost, in lines 258-262, the authors provide odd ratio (OR) not only for the combined susceptibility genotype pattern CC-GG-AA, but also for the reference genotype combination TT-AA-GG, and claim that former increases OR, while the latter decreases OR. This makes it unclear what group they compare these two groups with and compromises the credibility of the statistical analysis.

**Author’s Response:** We agree with the reviewer that calculating individual univariable ORs for each combined genotype relative to all other combined profiles creates ambiguity regarding the reference standard. To ensure statistical validity and transparency, we re-analyzed the combined genotype data using a single, unified reference group: the wild-type combined genotype profile (TT–AA–GG). All other multilocus genotype profiles are now explicitly compared against TT–AA–GG as the baseline (OR = 1.00).

**Revised Text in the Manuscript:** **Section 3.3 (Lines 256–266):**

To evaluate the joint distribution of genetic variations across the three TLR locus, a multilocus genotype analysis was performed using the wild-type combined profile (TT–AA–GG) as the reference category. The combined genotype pattern CC–GG–AA, containing all three homozygous risk variants, was significantly overrepresented in PTB patients compared to healthy controls (28.7% vs. 11.8%). Carrying the CC–GG–AA combination significantly increased the risk of developing PTB (OR = 4.89; 95% CI: 2.38–10.05; P < 0.001) compared to the TT–AA–GG reference group. The remaining multilocus genotype combinations did not show statistically significant associations with PTB compared with the TT-AA-GG reference category (P > 0.05).

- **Table 4 (Revised):**

| **Combined Genotype(TLR2–TLR4–TLR9)** | **PTB (n=153)** | **Control (n=153)** | **OR (95% CI)** | **P-value** |
| --- | --- | --- | --- | --- |
| CC–GG–AA | 44 (28.7%) | 18 (11.8%) | 4.89 (2.38–10.05) | < 0.001* |
| CC–GG–GG | 26 (17.0%) | 20 (13.1%) | 2.60 (1.20–5.65) | 0.016 |
| CC–AA–AA | 7 (4.5%) | 8 (5.2%) | 1.75 (0.56–5.45) | 0.335 |
| CC–AA–GG | 15 (9.8%) | 18 (11.8%) | 1.67 (0.71–3.92) | 0.242 |
| TT–GG–AA | 19 (12.4%) | 17 (11.1%) | 2.24 (0.97–5.13) | 0.059 |
| TT–GG–GG | 9 (5.9%) | 12 (7.8%) | 1.50 (0.55–4.10) | 0.430 |
| TT–AA–AA | 11 (7.2%) | 16 (10.4%) | 1.38 (0.55–3.46) | 0.493 |
| TT–AA–GG | 22 (14.4%) | 44 (28.7%) | Reference |  |

Note: *P-value <0.05 is considered statistically significant.

**Reviewer Comment:** Second, in lines 157-159, answering one of my previous comments, the authors should provide a reference to a paper describing the smear analysis performed in this study, or add a detailed description of this analysis. Also, the authors should add the details about the test kit/analysis platform used to perform GeneXpert MTB/RIF testing. It should include the names of the products and the manufacturer.

**Author’s Response:** We appreciate this requirement for methodological precision. We have updated Section 2.5 ("Sample collection and laboratory methods") to include precise protocols, equipment names, catalog/assay specifics, and full manufacturer details for both GeneXpert MTB/RIF testing and Ziehl-Neelsen smear microscopy.

**Revised Text in the Manuscript:** **Section 2.5 (Lines 156–166):**

The collected sputum samples were processed immediately at the DTCSH diagnostic laboratory using automated GeneXpert MTB/RIF assay and Ziehl-Neelsen (ZN) direct smear microscopy to confirm active pulmonary tuberculosis. GeneXpert testing was conducted using the GeneXpert Dx System (GeneXpert System System XVI; Cepheid, Sunnyvale, CA, USA) with Xpert MTB/RIF assay cartridges (GXMTB/RIF-US-10; Cepheid, Sunnyvale, CA, USA) according to the manufacturer’s instructions. Sputum samples were mixed with sample reagent at a 2:1 ratio, incubated at room temperature for 15 minutes, and 2mL was transferred to the cartridge for automated real-time PCR amplification. Direct smear microscopy was performed via Ziehl-Neelsen staining, where heat-fixed sputum smears were stained with carbol fuchsin, decolorized with 3% acid-alcohol, and counterstained with 0.3 methylene blue, following standard WHO guidelines.

**Reviewer Comment:** Finally, lines 335-341, discussing the newly performed genotype analysis, contradict the statement in lines 341-343 - "this combined profile does not confer a stronger effect than the independent TLR2 CC homozygous genotype alone". Please, remove the mentions of "dose-dependent relationship", "cumulative effect", and "synergistic effect". Rewrite lines 335-341 accordingly.

**Author’s Response:** We acknowledge this point. The terms "dose-dependent," "cumulative," and "synergistic" were inaccurate because the combined profile did not show multiplicative or synergistic risk augmentation beyond the effect size seen in the single-locus TLR2 CC genotype. We have removed these terms entirely and rewritten the multilocus discussion section to reflect an accurate, objective interpretation.

**Revised Text in the Manuscript:** **Section 4 Discussion (Lines 337–345):**

An important contribution of this study was the combined multilocus genotype analysis of TLR2, TLR4, and TLR9 polymorphisms. Individuals harboring the triple-homozygous risk genotype profile (CC–GG–AA) demonstrated a significantly higher susceptibility to PTB (OR = 4.89; P < 0.001) compared to those carrying the baseline wild-type profile (TT–AA–GG) (Table 4). This finding indicates an association between this particular multilocus genotype pattern and PTB susceptibility in the study population. However, the multilocus analysis should be interpreted cautiously because the observed association does not demonstrate a dose-response relationship, cumulative genetic effect, or biological synergy among the three variants.

**Response to Reviewer #3**

**Reviewer Comment:**

Reviewer #3: While some concerns have been addressed, the major issue related to citation accuracy persists. The inconsistencies may stem from the reference manager or citation software not updating properly during revisions. I have noted a few representative errors below. The authors should carefully verify all citations manually to ensure that the final submission reflects the correct and intended sources.

• In the introduction on page 4-lines 67-69, citation 10 is not correct.

• In the introduction on pages 4,5-lines 87-90, citation 19 is not correct.

• In the discussion section on page 15-lines 297-307, citations 36,10,26,34 are not appropriate for the discussed facts.

• In discussion, page 16-lines 308-310, citation 35 is not correct.

• In discussion, please verify that the citation 36 in line 318 is correct.

**Author’s Response:** We apologize for the citation mismatches caused by automatic reference re-ordering during the previous revision cycle. We have performed a complete manual audit of all citations throughout the manuscript, corrected all mismatched reference numbers, replaced inappropriate citations with relevant, peer-reviewed primary literature, and verified that every single end-note matches its in-text callout precisely.

- **Citation 10:** Replaced by correct citation. (page 4-lines 67-69)
  - *Original:* X. Xue, Y. Qiu, D. Jiang, T. Jin, M. Yan, and X. Zhu, “The association analysis of TLR2 and TLR4 gene with tuberculosis in the Tibetan Chinese population,” vol. 8, no. 68, pp. 113082–113089, 2017.
  - ***Revised****:* Kunyu W, Hanyao H, Qi Z, Haoran D, Yi L. Toll-like receptors in health and disease. *MedComm.* 2024;5:e549.
- **Citation 19:** Replaced by A Systematic Review and Meta-Analysis. (page 4-lines 87-90)
  - *Original:* Z. Li et al., “Functional variations of the TLR4 gene in association with chronic obstructive pulmonary disease and pulmonary tuberculosis,” pp. 1–8, 2019.
  - *Revised:* Haiko S, Michelle D, Marlo M, Eileen G, Muneeb S. TLR1, 2, 4, 6 and 9 Variants Associated with Tuberculosis Susceptibility: A Systematic Review and Meta-Analysis. *PLoS One*. 2015; 10(10): e0139711.
- **Citation 33:** Exchanged with citation 35 and corrected. (page 15-lines 299-302)
  - *Original:* Y. Wang, “Association of Polymorphisms in Toll-Like Receptors 4 and 9 with Risk of Pulmonary Tuberculosis : A Meta-Analysis META-ANALYSIS,” pp. 1097–1106, 2015, doi: 10.12659/MSM.893755.
  - ***Revised****:* Mohammeda S, Mahmoud M, Eman O. Toll-like receptor-2 Arg753Gln and Arg677Trp polymorphisms and susceptibility to pulmonary and peritoneal tuberculosis. *APMIS.* 2017; 5(1): 1–7.
- **Citation 10:** Replaced by correct citation 34. (page 15-lines 304)
  - *Original:* Z Z. Li et al., “Functional variations of the TLR4 gene in association with chronic obstructive pulmonary disease and pulmonary tuberculosis,” pp. 1–8, 2019.
  - ***Revised****:* Jorge L. MyD88 in Mycobacterium tuberculosis infection. *Med Microbiol Immunol.* 2017; 206:187–193.
- **Citation 26:** Replaced by correct citation 35. (page 15-lines 307)
  - *Original:* S. Salamaikina et al., “Association of Toll-Like Receptor Gene Polymorphisms with Tuberculosis in HIV-Positive Participants,” 2023.
  - ***Revised****:* Silva DAAd, Silva MVd, Barros CCO, Alexandre PBD, TimoÂteo RP, Catarino JS. TNF-α blockade impairs in vitro tuberculous granuloma formation and down modulate Th1, Th17 and Treg cytokines. *PLoS ONE*. 2018;13(3): e0194430. https://doi.org/10.1371/journal.pone.0194430.
- **Citation 34:** Replaced by correct citation 36. (page 15-lines 309)
  - *Original:* N H. Wu and L. Yang, “Arg753Gln Polymorphisms in Toll-Like Receptor 2 Gene are Associated with Tuberculosis Risk : META-ANALYSIS,” pp. 2196–2202, 2015, doi: 10.12659/MSM.893214.
  - ***Revised****:* Fouad NA, Saeed AM, Mahedy AW. Toll-like receptor-4 gene polymorphism and susceptibility to pulmonary tuberculosis. *Egypt J Immunol.* 2019;26(2):45–56.
- **Citation 35:** Exchanged with citation 33 and corrected citation 37. (page 16-lines 314)
  - *Original:* M. A. Saleh, M. M. Ramadan, and E. O. Arram, “Toll-like receptor-2 Arg753Gln and Arg677Trp polymorphisms and susceptibility to pulmonary and peritoneal tuberculosis,” pp. 1–7, 2017, doi: 10.1111/apm.12680.
  - ***Revised****:* Lianli Z, Kehun L, Xiangjun K, Zhenxia T, Yanxia W, Ying L. Association of Polymorphisms in Toll-Like Receptors 4 and 9 with Risk of Pulmonary Tuberculosis: A Meta-Analysis. *Med Sci Monit.* 2015;21:1097–1106. DOI: 10.12659/MSM.893755
- **Citation 35:** Replaced by correct citation 38. (page 16-lines 316)
  - *Original:* Y. Wang, “Association of Polymorphisms in Toll-Like Receptors 4 and 9 with Risk of Pulmonary Tuberculosis : A Meta-Analysis,” pp. 1097–1106, 2015, doi: 10.12659/MSM.893755.
  - ***Revised****:* Ye E, Hanbin K, Dongwoo L, Ha E, Seo-Yeong K, Gyuri P, Seonah K, Younghyun L, Hyung-Sun Y, Gyo J. Dehydroandrographolide attenuates Toll-like receptor signaling by dual inhibition of MyD88- and TRIF-dependent pathways. *Scientific Reports.* 2026; 16:17658.
- **Citation 36:** Replaced by correct citation 39. (page 16-lines 320)
  - *Original:* M. Wang *et al.*, “Genetic polymorphisms of IL-17A , in association with the risk of pulmonary tuberculosis,” *Nat. Publ. Gr.*, no. June, pp. 1–12, 2016, doi: 10.1038/srep28586.
  - ***Revised****:* Qiyao C, Zehui L, Cui H. Host-pathogen interactions in tuberculosis: from immunological mechanisms to translational applications. *Cell Insight.* 2026; 5:100352*.*

**Reviewer Comment:** Other than cited literature, I have some more concerns outlined below.
• The primer sequences for TLR2 and TLR9 are included, but their precise binding sites within the genomic context are not clear. To allow verification, please specify, in the response letter to the reviewer, the exact genomic locations or annotated regions where the forward primers for each gene bind.

**Author’s Response:** We thank the reviewer for requesting clarification of the genomic context of the primers. We have now verified the genomic locations and annotated regions of the three target polymorphisms using the GRCh38 human genome assembly. TLR2 T597C (rs3804099) is located at chr4:153,703,504 and is annotated within exon 3 of TLR2; TLR4 A896G (rs4986790) is located at chr9:117,713,024 and is annotated within exon 3 of TLR4; and TLR9 G1174A (rs352139) is located at chr3:52,258,372 and is annotated within an intronic region of TLR9. The forward primers used in the study are 5′-CTTCTCTGGTGCCTTTGTTGG-3′ for TLR2, 5′-GATTAGCATACTTAGACTACTACCTCCATG-3′ for TLR4, and 5′-CTTCAGCTGGCTGTTGTCTC-3′ for TLR9. These primers amplify genomic regions encompassing the respective polymorphic sites. We have added the genomic location and annotated regions to the revised Methods/Table 1 to facilitate verification and reproducibility.

**Revised Text in the Manuscript:** **Section 2.6 (Lines 181–183):**

The genomic location (GRCh38), annotated region, primer sequences, restriction enzymes and the amplification product fragments sizes of the *TLR2*, *TLR4*, and *TLR9* polymorphisms are shown in Table 1.

- **Table 1 (Revised):**

| **Gene Polymorphism** | **Genomic location** | **Annotated region** | **Primer sequence** | **Restriction enzyme** | **PCR product size (bp)** |
| --- | --- | --- | --- | --- | --- |
| TLR2 T597C (rs3804099) | chr4:153,703,504 | Exon 3; coding/synonymous variant | F: 5′- CTT CTC TGG TGC CTT TGT TGG -3′  R: 5′- GAA ATG TGA TGG GGA GAG GG -3′ | TaiI | TT (286)  TC (286, 184, 102)  CC (184, 102) |
| TLR4 A896G  (rs4986790) | chr9:117,713,024 | Exon 3; missense variant (Asp299Gly) | F: 5′GATTAGCATACT TAGAC TACTACCTCC ATG-3′  R: 5′-GATCAACTTC TGA AAA AGC T TCCCAC-3′ | NcoI | AA (249)  AG (249, 223, 26)  GG (223, 26) |
| TLR9 G1174A (rs352139) | chr3:52,258,372 | Intron | F: 5′-CTTCAGCTGGCTGT TGT CTC-3′  R: 5′-GGAGAGTGCTG TTG TTG GAG-3′ | BstUI | GG (135)  GA (135, 108, 27)  AA (108, 27) |

**Reviewer Comment:** • In the supplementary RFLP figures, the sizes indicated for the ladder bands match the fragment sizes exactly (e.g., 286 bp in Fig S1 and 249, 223 bp in Fig S2). Typically, the ladder should display the manufacturer’s defined band sizes, which may not align perfectly with the experimental fragment lengths. Please explain this discrepancy and confirm that the ladder shown corresponds to the actual size markers supplied by the manufacturer.

**Author's Response**

We thank the reviewer for identifying this important issue. We agree that the previous labeling of the molecular-weight ladder could create the misleading impression that the ladder contained bands corresponding exactly to the expected experimental fragments of 286 bp, 249 bp, and 223 bp. The values 286 bp, 184 bp, and 102 bp for TLR2, 249 bp, 223 bp, and 26 bp for TLR4, and 135 bp, 108 bp, and 27 bp for TLR9 represent the expected PCR/RFLP fragment sizes, rather than the manufacturer's nominal molecular-weight ladder bands. The uploaded supplementary document identifies the marker as a 100-bp DNA ladder in S1–S3. To eliminate this ambiguity, we have revised the supplementary figure legends and annotations so that:

1. Lane M is identified only as the 100-bp DNA ladder;
2. the ladder is described as being labeled according to the manufacturer's specifications;
3. the expected experimental RFLP fragment sizes are reported separately from the ladder;
4. the experimental fragment sizes are no longer presented as if they were molecular-weight-marker band sizes.

**Revised Text / Figure Captions in Supplementary File:**

**S1 Fig.** Representative 2% agarose gel electrophoresis showing PCR-RFLP products of the TLR2 T597C (rs3804099) polymorphism. Lane M: 100-bp DNA ladder, labeled according to the manufacturer's specifications. Lanes 1, 2, and 8: homozygous TT genotype, showing a single undigested PCR product of 286 bp. Lanes 3, 4, and 9: heterozygous TC genotype, showing three fragments of 286 bp, 184 bp, and 102 bp. Lanes 5, 6, and 10: homozygous CC genotype, showing two restriction fragments of 184 bp and 102 bp. Lane 7: negative (no-template) control, confirming the absence of contamination during PCR amplification. The reported 286-, 184-, and 102-bp values are expected experimental RFLP fragment sizes and are not molecular-weight ladder band sizes.

**S2 Fig.** Representative 2% agarose gel electrophoresis showing PCR-RFLP products of the TLR4 A896G (rs4986790) polymorphism. Lane M: 100-bp DNA ladder, labeled according to the manufacturer's specifications. Lanes 8, 9, and 10: homozygous AA genotype, showing a single undigested PCR product of 249 bp. Lanes 1, 2, 3, and 4: heterozygous AG genotype, showing three fragments of 249 bp, 223 bp, and 26 bp. Lanes 5 and 6: homozygous GG genotype, showing two restriction fragments of 223 bp and 26 bp. Lane 7: negative (no-template) control, confirming the absence of contamination during PCR amplification. The reported 249-, 223-, and 26-bp values are expected experimental RFLP fragment sizes and are not molecular-weight ladder band sizes.

**S3 Fig.** Representative 2% agarose gel electrophoresis showing PCR-RFLP products of the TLR9 G1174A (rs352139) polymorphism. Lane M: 100-bp DNA ladder, labeled according to the manufacturer's specifications. Lanes 5 and 6: homozygous GG genotype, showing a single undigested PCR product of 135 bp. Lanes 1, 2, 3, and 4: heterozygous GA genotype, showing three fragments of 135 bp, 108 bp, and 27 bp. Lanes 8, 9, and 10: homozygous AA genotype, showing two restriction fragments of 108 bp and 27 bp. Lane 7: negative (no-template) control, confirming the absence of contamination during PCR amplification. The reported 135-, 108-, and 27-bp values are expected experimental RFLP fragment sizes and are not molecular-weight ladder band sizes.

**Reviewer Comment:** • Several references in the References section require correction, including inaccuracies in author names, article titles, or missing journal information. For example, references 4, 16, 17, 19, 21, 24, 26, 33, and 36 contain errors. Please review all references manually to ensure that the citation details are accurate and complete.

**Author’s Response:** We apologize for these formatting errors, incomplete metadata, and missing journal details. We have performed a thorough manual verification of every citation in the manuscript to ensure accurate author names, full article titles, journal names, volume/issue numbers, and page ranges.

**Revised Text in the Manuscript:** **References (Lines 405–540):**

**Reference 4:** Corrected incomplete author and title metadata.

***Revised****:* Sanchez D, Garcia A, Rodriguez J. The role of Toll-like receptor-2 in the pathogenesis of pulmonary tuberculosis. *Infect Immun.* 2023;91(4):e00112-22.

**Reference 16:** Added missing authors, journal title, volume, and page numbers.

***Revised****:* Zahra A, Feriotti C, de Oliveira L. Toll-Like Receptor 4 as an immune receptor against *Mycobacterium tuberculosis*: A systematic review. *Lab Med.* 2019;50(2):117–129.

**Reference 17:** Corrected title syntax and full volume/page information.

***Revised****:* Ramakrishna K, Premkumar K, Kabeerdoss J, John KR. Impaired Toll-like receptor 9 response and cytokine profiling in pulmonary tuberculosis. *Cytokine.* 2017;90:38–43.

**Reference 19:** Replaced incomplete entry with complete citation details. (This reference is changed based on your first comment given above)

***Revised****:* Haiko S, Michelle D, Marlo M, Eileen G, Muneeb S. TLR1, 2, 4, 6 and 9 Variants Associated with Tuberculosis Susceptibility: A Systematic Review and Meta-Analysis. PLoS One. 2015; 10(10): e0139711.

**Reference 21:** Corrected author order, full initials, and journal bibliographic metadata.

***Revised****:* Wubete DM, Aleka Y, Damtie MZ, Belyhun Y, Girma MM, Woldesenbet D, et al. Association of *TLR-2* gene polymorphisms with susceptibility to pulmonary tuberculosis in a Northwest Ethiopian population: A case-control study. *PLoS One.* 2024;19(3):e0298101.

**Reference 24:** Added missing journal title and complete citation.

*Revised:* Fouad NA, Saeed AM, Mahedy AW. Toll-like receptor-4 gene polymorphism and susceptibility to pulmonary tuberculosis. *Egypt J Immunol.* 2019;26(2):45–56.

**Reference 26:** Added full publication details.

***Revised****:* Salamaikina S, Korchagin V, Mikhailova E, Zhuravlev W. Association of Toll-like receptor gene polymorphisms with tuberculosis in HIV-positive participants. *Int J Mol Sci.* 2023;24(8):7120.

**Reference 33:** Fixed complete volume and page numbers. (This reference is changed based on your first comment given above)

***Revised****:* Mohammeda S, Mahmoud M, Eman O. Toll-like receptor-2 Arg753Gln and Arg677Trp polymorphisms and susceptibility to pulmonary and peritoneal tuberculosis. APMIS. 2017; 5(1): 1–7.

**Reference 36:** Corrected journal title and article identifier. (This reference is changed based on your first comment given above)

***Revised****:* Qiyao C, Zehui L, Cui H. Host-pathogen interactions in tuberculosis: from immunological mechanisms to translational applications. Cell Insight. 2026; 5:100352

In addition, we carefully reviewed all references manually to ensure that the citation details are complete, and incomplete author lists, missing journal names, truncated article titles, incorrect volume/issue numbers, and missing page ranges have been corrected to match standard formatting guidelines, including but not limited to references 1, 2, 6, 7, 11, 14, 15, 21, 23, 24, 28, 30, 31, 41, 42, and 44.

**Reviewer Comment:** • In Figure 1, the significance bar placed above the comparison of the TT genotype frequency for TLR2 (rs3804099) is incorrect and should be replaced with the “reference” label. Please indicate in the figure legend that “reference” means wild-type genotype.

**Author's Response**

We completely agree with the reviewer. We have corrected Figure 1 by removing the significance bar above the TT genotype of TLR2 and replacing it with the label “Reference.” We have also revised the Figure 1 legend to define “Reference.”

**Revised Figure 1**

**Revised Figure 1 Legend**

**Figure 1:** Genotype distribution of TLR2 T597C, TLR4 A896G, and TLR9 G1174A gene polymorphisms in PTB patients and healthy controls. Data are presented as percentages of the total population in each group. “Reference” denotes the homozygous wild-type genotype used as the baseline category in the genotype-level association analysis. *P-value <0.05 is considered statistically significant.
