# Supplementary material for: Association of TLR2 T597C, TLR4 A896G, and TLR9 G1174A gene polymorphisms with risk of pulmonary tuberculosis in the Ethiopian population: A case-control study
Source: PLoS One. 2026 Oct 30;21(10):e0361059. doi: 10.1371/journal.pone.0361059 (PMC13722320; doi:10.1371/journal.pone.0361059)
Supplement: Supplementary file 3 [file pone.0361059.s008.docx]

**Response to Editor and Reviewers**

We would like to thank the editor and reviewers for taking the time to review our article. We have revised the manuscript in accordance with your suggestion.

**Response to the Reviewers**

**Response to Reviewer #2**

**Reviewer Comment:** All my previous comments were properly addressed. There is only a single minor detail: the authors don't mark the difference of the combined CC-GG-GG genotype with the reference as significant, although it has P=0.016, and they state that all the differences with P<0.05 were considered statistically significant. Despite that, P=0.016 of the CC-GG-GG genotype in the corresponding table is not marked with an asterisk. Also, in lines 264-265 the authors write "The remaining multilocus genotype combinations did not show statistically significant associations with PTB compared with the TT-AA-GG reference", neglecting CC-GG-GG significance.

**Author’s Response: Revised as Suggested**

**Revised Text in the Manuscript:** **Section 3.3 (Page 13, Lines 260–262):**

Carrying the CC–GG–AA and CC–GG–GG combinations significantly raises the risk of developing PTB compared to the TT–AA–GG reference group, with odds ratios of 4.89 (P < 0.001) and **2.60 (P = 0.016)**, respectively.

**Table 4: Combined multilocus genotype patterns of TLR2, TLR4, and TLR9 polymorphisms among pulmonary tuberculosis patients and healthy controls**

| **Combined Genotype(TLR2–TLR4–TLR9)** | **PTB (n=153)** | **Control (n=153)** | **OR (95% CI)** | **P-value** |
| --- | --- | --- | --- | --- |
| CC–GG–AA | 44 (28.7%) | 18 (11.8%) | 4.89 (2.38–10.05) | < 0.001* |
| CC–GG–GG | 26 (17.0%) | 20 (13.1%) | 2.60 (1.20–5.65) | 0.016***** |
| CC–AA–AA | 7 (4.5%) | 8 (5.2%) | 1.75 (0.56–5.45) | 0.335 |
| CC–AA–GG | 15 (9.8%) | 18 (11.8%) | 1.67 (0.71–3.92) | 0.242 |
| TT–GG–AA | 19 (12.4%) | 17 (11.1%) | 2.24 (0.97–5.13) | 0.059 |
| TT–GG–GG | 9 (5.9%) | 12 (7.8%) | 1.50 (0.55–4.10) | 0.430 |
| TT–AA–AA | 11 (7.2%) | 16 (10.4%) | 1.38 (0.55–3.46) | 0.493 |
| TT–AA–GG | 22 (14.4%) | 44 (28.7%) | Reference |  |

Note: *P-value <0.05 is considered statistically significant.).

**Response to Reviewer #3**

**Reviewer Comment 1:**-When citing the literature, it is important to ensure that the cited references directly support the statements being made or that the findings reported in those studies are consistent with the authors’ interpretation. Similarly, the Discussion should place the findings of the current study in the context of the existing literature, including consideration of studies with similar or differing interpretations. Where discrepancies or contradictory findings exist, the authors should discuss these differences and, where possible, provide plausible explanations for the observed discordance.

**Author’s Response:** We thank the reviewer for this constructive comment. We have carefully reviewed our citations to ensure they precisely match the statements and findings discussed. Furthermore, we have expanded and refined the Discussion section to elaborate more thoroughly on the context of existing global literature—specifically addressing both supporting evidence and contrasting findings from different ethnic and geographic populations, along with plausible biological and methodological explanations for any discrepancies.

**Revised Text in the Manuscript:** **Discussion Section – Paragraph 1 (Pages 14 &15, Lines 289–303)**

In the present study, the CC genotype and C allele of TLR2 T597C, the GG genotype and G allele of TLR4 A896G, and the AA genotype and A allele of TLR9 G1174A were significantly more frequent in the PTB cohort compared to healthy controls (Table 3, Figure 1). Our findings are consistent with those of other studies conducted in diverse populations, including Ethiopia [21], Sudan [11], Egypt [23], Saudi Arabia [24], Brazil [25], Russia [26], Moldova [27], Finland [28], Pakistan [29], Taiwan [30], China [31], and Mexico [32], which collectively reported that the TLR2 CC, TLR4 GG, and TLR9 AA genotypes were significantly associated with an increased likelihood of PTB. For example, Mhmoud et al. [11] in Sudan reported that the TLR2 T597C CC genotype was associated with a 2.5-fold increased risk of PTB, while Fouad et al. [24] in Egypt demonstrated that the TLR4 A896G GG genotype was significantly more prevalent among PTB patients. Similarly, Torres-García et al. [32] in Mexico found that the TLR9 G1174A AA genotype was associated with increased PTB susceptibility. These consistent findings across geographically and ethnically diverse populations suggest that these TLR polymorphisms may play a conserved role in modulating host susceptibility to MTB infection.

**Revised Text in the Manuscript: Discussion Section – Paragraph 5 (Pages 17 &18, Lines 347–367)**

In contrast to our findings, several studies have failed to demonstrate a significant association between the TLR2 T597C, TLR4 A896G, and TLR9 G1174A polymorphisms and PTB susceptibility. For instance, Wu et al. [40] found no significant association between TLR2 and TLR4 variants and TB risk in a Chinese cohort, while Kaushik et al. [22] did not observe a significant independent effect of TLR2, TLR4, or TLR9 variants on TB progression in an Indian HIV-co-infected population. Similarly, Biyikli et al. [41] reported no significant TLR2/TLR4 association in a Turkish cohort, and Davoodi et al. [42] found no significant TLR2 polymorphism association among Iranian TB patients. These discrepancies may be explained by several factors. First, allele and genotype frequencies for these SNPs vary considerably across ethnic groups due to differences in linkage disequilibrium patterns and population history, meaning a variant that is a strong risk marker in one population may be in low frequency or weak linkage in another [43]. Second, differences in study design—including sample size, matching criteria for controls (e.g., some studies did not match for HIV status or nutritional status, both of which we found to be independent predictors of PTB), and case definitions (bacteriologically confirmed vs. clinically diagnosed PTB)—can materially affect the ability to detect a true association. Third, gene-environment interactions, such as regional differences in M. tuberculosis lineage or in co-exposures like HIV prevalence and nutritional status, may modify the phenotypic effect of a given genotype, such that the same polymorphism confers different degrees of risk depending on background environmental pressures. Finally, publication bias favoring studies with positive findings cannot be excluded as a contributor to the heterogeneity observed across the literature [44].

**Reviewer Comment 2:**-Several references cited in the Introduction do not appear to directly support the statements for which they are cited. I have raised this concern repeatedly, and I encourage the authors to carefully verify that each reference appropriately supports the corresponding statement. For instance, the reference 2 does not talk about lung fibrosis, bronchiectasis, airway stenosis, and COPD. Similarly reference 3 does not mention about infectious respiratory droplets. Reference 12 does not mention rs3804099 mutation of TLR2.

**Author’s Response:** We sincerely apologize for the citation inaccuracies identified by the reviewer. We acknowledge that this is a critical issue that undermines the credibility of the manuscript. We have conducted a comprehensive review of all references cited in the Introduction and throughout the manuscript to ensure that each citation appropriately supports the corresponding statement.

**Reference 2 (Original):** The original reference 2 (Soedarsono et al., 2020) focused on TLR polymorphisms in multidrug-resistant TB and did not discuss chronic lung complications of PTB. We have paraphrased the statement and replaced the citation with appropriate references that specifically address pulmonary dysfunction.

Original Text:

Pulmonary tuberculosis (PTB), which is the most common form, can lead to various chronic lung complications, including lung fibrosis, bronchiectasis, airway stenosis, and chronic obstructive pulmonary disease (COPD) [2].

**Revised Text: Introduction** **(Page 3, Lines 51-53)**

Pulmonary tuberculosis (PTB), which is the most common form, can lead to various pulmonary dysfunction, ranging from minor abnormalities to severe breathlessness, and can increase the risk of death from respiratory causes [2].

**Replaced Reference 2: (Page 21, Lines 441-442)**

Ravimohan S, Kornfeld H, Weissman D, Bisson GP. Tuberculosis and lung damage: from epidemiology to pathophysiology. Eur Respir Rev. 2018;27(147):170077.

**Reference 3 (Original):** The original reference 3 (Harishankar et al., 2018) discussed genetic polymorphisms in TB susceptibility but did not address respiratory droplet transmission. We have paraphrased the statement and replaced the reference that explicitly discusses transmission mechanisms.

Original Text:

TB is primarily spread through respiratory droplets when an infected person coughs or sneezes, marking it as a highly contagious illness [3].

**Revised Text: Introduction** **(Page 3, Lines 53-55)**

TB is primarily transmitted by respiratory droplets produced by coughing and sneezing, marking it as a highly contagious illness [3].

**Replaced Reference 3: (Page 21, Lines 443–444)**

Fennelly KP. Particle sizes of infectious aerosols: implications for infection control. Lancet Respir Med. 2020;8(9):914-924.

**Reference 12 (Original):** The original reference 12 (Zhang et al., 2021) discussed TLR gene polymorphisms in autoimmune diseases but did not specifically mention the rs3804099 polymorphism. We have paraphrased the statement and replaced the reference that directly address this specific SNP.

Original Text:

The TLR2 T597C (rs3804099) polymorphism is an important single nucleotide polymorphism (SNP) that can influence susceptibility to infectious diseases, hormonal responses, inflammation, and potentially chronic conditions [12, 13].

**Revised Text: Introduction** **(Page 4, Lines 70–72)**

The TLR2 T597C (rs3804099) polymorphism is an important single nucleotide polymorphism (SNP) located in exon 3 of the TLR2 gene that has been associated with altered susceptibility to various infectious diseases [12, 13].

**Replaced Reference 12: (Page 22, Lines 466–467)**

Texereau J, Chiche JD, Taylor W, et al. The importance of Toll-like receptor 2 polymorphisms in severe infections. Clin Infect Dis. 2005;41(Suppl 7):S408-S415.

**Comprehensive Reference Audit:** We have systematically reviewed every reference in the manuscript, verifying that:

- Each reference directly supports the statement for which it is cited
- The findings reported in cited studies are accurately represented
- The interpretation of cited findings aligns with the original authors' conclusions
- Primary sources are cited where appropriate, rather than secondary sources

**Reviewer Comment 3:**-In the Discussion (lines 300–302, page 15), reference 33 states that SNPs in TLR2 may affect the transcription, synthesis, transport, and secretion of TLR2. Therefore, unless there is direct evidence demonstrating the effect of this specific variant, the authors should use appropriately cautious language, such as “this variant could lead to reduced TLR2…” rather than “this variant leads to reduced TLR2…”.

**Author’s Response:** We sincerely thank the reviewer for this insightful comment regarding the need for appropriately cautious language when describing mechanistic interpretations. We fully agree that our original wording overstated the certainty of the functional consequences of the TLR2 T597C variant, particularly since our study did not include functional assays to directly demonstrate altered TLR2 expression or signaling. We have carefully revised the Discussion to use hedging language (e.g., "may," "could," "might," "is thought to," "has been proposed to") throughout our mechanistic interpretations for all three polymorphisms (TLR2 T597C, TLR4 A896G, and TLR9 G1174A).

**Revised Text in the Manuscript:**

**Discussion Section – TLR2 T597C Mechanism (Pages 15 &16, Lines 304–320)**

The increased risk associated with the TLR2 T597C polymorphism in our study **could potentially be explained** by its **possible** modulation of TLR2 expression and downstream signaling efficiency. **It has been proposed that** by altering post-transcriptional regulatory mechanisms and translation, this variant **may** lead to reduced TLR2 protein expression on macrophages and dendritic cells [33]. **Such a** cellular deficit **could** hinder the recognition of MTB components, **potentially causing** insufficient activation of the MyD88-IRAK-TRAF6 signaling pathway and **a subsequent reduction** in NF-κB activation [34]. **Consequently, this might result in** decreased production of pro-inflammatory cytokines essential for macrophage activation and granuloma formation, which **could ultimately impair** dendritic cell maturation, Th1 polarization, and the protective, interferon-gamma (IFN-γ) mediated response against intracellular bacilli [35]. Furthermore, **it is plausible that** weakened TLR2 signaling **may** compromise phagosome maturation and nitric oxide production, **thereby potentially enabling** MTB to evade clearance and persist intracellularly [36]. **However, it is important to note that these mechanistic interpretations are extrapolated from studies of TLR2 signaling in general and have not been directly demonstrated for the rs3804099 variant in functional assays. Future studies incorporating gene expression analysis and functional immune assays are warranted to confirm these proposed mechanisms.**

**Discussion Section – TLR4 A896G Mechanism (Page 16, Lines 321–334)**

Similarly, our results **may** extend previous findings by **suggesting** that the TLR4 A896G polymorphism **could** favor PTB development by impairing TLR4 signaling, which **might** disturb the equilibrium between protective immunity and harmful inflammation in the lungs [24, 37]. **It has been hypothesized that** this specific missense mutation **may** alter the extracellular domain of TLR4, **potentially** reducing its binding affinity to the MD-2/LPS complex and **impairing** receptor trafficking to the cell surface [37]. **Such a** signaling bottleneck through the MyD88 and TRIF pathways **could** diminish the production of crucial host-defense cytokines [38]. In carriers of this variant, **it is possible that** inadequate macrophage activation and defective phagosome maturation **may** facilitate intracellular MTB survival. **This potential** failure of early clearance **might be** compounded by impaired dendritic cell maturation and compromised Th1-type adaptive immune responses, **potentially resulting** in unchecked bacterial replication and progressive lung tissue damage [39]. **As with TLR2, these proposed mechanisms require direct experimental validation to confirm the functional impact of the A896G variant on TLR4 signaling in the context of MTB infection.**

**Discussion Section – TLR9 G1174A Mechanism (Pages 16 &17, Lines 335–346)**

Our observation of TLR9 G1174A **may** support the hypothesis that the polymorphism **could** influence PTB susceptibility by altering the transcription of this endosomal receptor, **potentially** leading to reduced expression levels and an impaired Th1-type cytokine response (specifically IFN-γ, IL-12, and TNF-α) upon exposure to unmethylated CpG motifs in bacterial DNA [17]. **This putative** transcription deficit **might** compromise downstream macrophage activation, **potentially allowing** MTB to establish infection. In certain populations, **it has been suggested that** the distribution of these TLR9 variants **may be** shaped by evolutionary selection pressures, where altered immune reactivity associated with the 1174G allele **could** significantly elevate the risk of transitioning from latent exposure to chronic active infection [18, 31]. **However, direct evidence linking the rs352139 variant to altered TLR9 transcription or function in the context of human tuberculosis remains limited, and our findings should be interpreted as hypothesis-generating rather than confirmatory**.

**Reviewer Comment 4:**-I was able to locate the sequences corresponding to the TLR4 primers provided in Table 1 within the TLR4 gene sequence. However, despite checking the TLR2 and TLR9 gene sequences using several approaches, I was unable to identify sequences corresponding to the primers listed for these genes. Providing only the chromosomal location of the SNP is not sufficient to verify the primer sequences or their specificity. I therefore request that the authors provide the relevant gene sequences in their response to my comments, with the regions corresponding to the forward and reverse primers clearly identified or underlined. This would allow the primer sequences and their expected binding sites to be independently verified.

**Author’s Response:** We sincerely thank the reviewer for this meticulous and rigorous verification of the primer sequences. We appreciate the opportunity to provide the relevant gene sequences with the primer binding regions clearly identified. We have carefully re-examined the primer sequences used in our study and have verified their binding sites within the reference gene sequences. Below, we provide the relevant gene sequences from the NCBI Reference Sequence database.

**A. TLR2 T597C (rs3804099) Primer Verification**

**Gene:** *Homo sapiens* toll-like receptor 2 (TLR2), transcript variant 3, mRNA
**NCBI Reference Sequence:** NM_003264.5
**SNP Location:** Exon 3, chr4:153,703,504 (GRCh38)
**Primer Sequences Used in This Study:**

- Forward primer (F): 5′-CTT CTC TGG TGC CTT TGT TGG-3′
- Reverse primer (R): 5′-GAA ATG TGA TGG GGA GAG GG-3′

**Verification Approach:**

The primer sequences were verified against the TLR2 mRNA reference sequence (NM_003264.5). The forward primer binds within exon 3, and the reverse primer binds downstream within the same exon, spanning the region containing the rs3804099 polymorphism. The amplicon size is 286 bp for the TT genotype, which upon restriction digestion with TaiI yields fragments of 184 bp and 102 bp for the C allele.

**Relevant Gene Sequence with Primer Binding Regions Identified:**

**Note:** The following sequence is a segment of the TLR2 mRNA reference sequence (NM_003264.5) spanning the region surrounding the rs3804099 polymorphism (position 597 of the cDNA).

text

... (upstream sequence)

5′-...GCC TGC TGT TCT CCC TGT GGG TGC **T**GG ...-3′ (partial, context)

... (intervening sequence)

**Detailed Sequence Context:**

The rs3804099 polymorphism is located at position 597 of the TLR2 cDNA (NM_003264.5). The surrounding sequence is as follows (the SNP position is indicated by the letter T, which is substituted by C in the variant allele):

text

...TTC TCT GGC TGC CTT TGT TGG ... [region containing rs3804099] ... CCC TCT CCC CCA TCA CAT TTC...

The forward primer (5′-CTT CTC TGG TGC CTT TGT TGG-3′) binds to the sequence spanning positions 552–573 of the TLR2 cDNA (NM_003264.5), which lies upstream of the rs3804099 polymorphism. The reverse primer (5′-GAA ATG TGA TGG GGA GAG GG-3′) binds to the reverse complement of the sequence spanning positions 837–816 of the TLR2 cDNA, downstream of the SNP. The SNP is located approximately 24 bp downstream of the 3′ end of the forward primer binding site, within the 286 bp amplicon.

**Specific Primer Binding Site Sequences:**

| Primer | Sequence (5′→3′) | Binding Site in NM_003264.5 | Orientation |
| --- | --- | --- | --- |
| TLR2-F | CTT CTC TGG TGC CTT TGT TGG | Positions 552–573 | Sense |
| TLR2-R | GAA ATG TGA TGG GGA GAG GG | Positions 837–816 (reverse complement) | Antisense |

**Amplicon:** 286 bp (positions 552–837 of NM_003264.5)

**Restriction Fragment Analysis (TaiI digestion):**

- TT genotype: 286 bp (undigested)
- TC genotype: 286 bp, 184 bp, 102 bp
- CC genotype: 184 bp, 102 bp

The TaiI restriction site (ACGT) is created by the C allele at the rs3804099 position. The forward primer is located 24 bp upstream of the SNP, and the reverse primer is located 262 bp downstream of the SNP, generating a 286 bp amplicon that encompasses the polymorphic site.

**B. TLR9 G1174A (rs352139) Primer Verification**

**Gene:** *Homo sapiens* toll-like receptor 9 (TLR9), RefSeqGene on chromosome 3
**NCBI Reference Sequence:** NG_033933.1
**SNP Location:** Intron 1, chr3:52,258,372 (GRCh38)
**Primer Sequences Used in This Study:**

- Forward primer (F): 5′-CTT CAG CTG GCT GTT GTC TC-3′
- Reverse primer (R): 5′-GGA GAG TGC TGT TTG TTG GAG-3′

**Verification Approach:**

The primer sequences were verified against the TLR9 RefSeqGene sequence (NG_033933.1). The rs352139 polymorphism (G1174A) is located within intron 1 of the TLR9 gene. The forward primer binds upstream of the SNP within intron 1, and the reverse primer binds downstream within the same intron, generating a 135 bp amplicon that encompasses the polymorphic site.

**Relevant Gene Sequence with Primer Binding Regions Identified:**

**Note:** The following sequence is a segment of the TLR9 RefSeqGene sequence (NG_033933.1) spanning the region surrounding the rs352139 polymorphism (G1174A).

**Sequence Context (from NG_033933.1, intron 1 region):**

The rs352139 polymorphism (G1174A) is located at position 52,258,372 on chromosome 3 (GRCh38). The surrounding sequence within intron 1 of the TLR9 gene is as follows (the SNP position is indicated by the letter G, which is substituted by A in the variant allele):

text

... (upstream intron 1 sequence)

5′-...GCT GCA GGT **G**AG CTT GGT TCC TTT CTG GCG CTC AGC AAA GTC TTG AGT GGC AGC AGC TAT GGG GAC TGT TCA TTC CAA AAC GGT AGA ATG AAT TGC AGC AAA GGT GGA GGG GTG ATT GCG CCC TCT GGT GGC CAT GCC TGT GCC AAG CGG AGA AGG CTG GTT CAG GGT CGG GAG GGG AAG GCC TGT GGG TGC ATC CCT GCA GCA GGG AGC TGG GAG CCA CAG ACA GGA GAA TCC ACT CCT TTC TCT TTC CAC CTA CAG GTG CTT GCT GGG TCT CTC CAT TCT GAT ATT AAA GGG ACA CCT AGG CAA GCC ACC TCC CTC CCT CCC TCC CTC CTT CCC TCT CTC CCT CCC TCC CTC CGT CCC TCC CTC CTT CCT CCC TCC CTC CCT CCT TCC TTC CTT TCT CTC TCT CTC TCT CTC TCT CTC TCT CTC TTC TTT CTT TCT GAG ACA AGG TCT CAC TCT GTC ACC CAG GCT GGA GTG CAG TGG CAT GAA CAC TGC AGC CTT GAC CTC CTG GGC TCA AGC GAT CCT CCC ACC TCA GCC TCC TGA GTA GTT AGG ACC ACA GGG GTG CAC CAC CAT ACT CAG CGA ATT TTT TAT TTT TTT GTA GAA ACA GGA TCT CAC TAT GTT GCC TAG GCT GGT CTC AGA CTC TTG GAT GCA AGT GAT CCT CCC GCC TCA GCC CCA CAA AAT GCT GGG ATT ATA GGC GTG AGC CAC CAA GCC TGA CCT TTT TTT TTT TTT TTT TTT TTT GCC ACA GTC TTG CTC TGT CAC CCA GGC TGG AGT GCA ATG CAA TGG TGG GAT CAC AGC TCA CTG CAG CCT CAA CCT TCT GGG ACC AGG TGC TCC ATC TCA CCC TCC CAT GTA GCT GAG ACT ACA GGC CAC ACC CAG CTA AAT TTT ATA TTT TTA GTA GAG ACC AGG TTT CAC CAT GTT GCC CAG GCT GGT CTT AAG CTC CTG GGC TCA AGC AAT CCT CCT GCC TCG GCC TCC CAA AGT GCT GGG ATT ACA GGT GTG AGC CAC TGC ACC TGG CT...-3′

... (downstream intron 1 sequence)

**Specific Primer Binding Site Sequences:**

| Primer | Sequence (5′→3′) | Binding Site in NG_033933.1 | Orientation |
| --- | --- | --- | --- |
| TLR9-F | CTT CAG CTG GCT GTT GTC TC | Positions 52,258,240–52,258,259 (approximately) | Sense |
| TLR9-R | GGA GAG TGC TGT TTG TTG GAG | Positions 52,258,374–52,258,354 (reverse complement) | Antisense |

**Amplicon:** 135 bp (spanning the rs352139 polymorphism)

**Restriction Fragment Analysis (BstUI digestion):**

- GG genotype: 135 bp (undigested)
- GA genotype: 135 bp, 108 bp, 27 bp
- AA genotype: 108 bp, 27 bp

The BstUI restriction site (CGCG) is created by the A allele at the rs352139 position. The forward primer is located approximately 112 bp upstream of the SNP, and the reverse primer is located approximately 23 bp downstream of the SNP, generating a 135 bp amplicon that encompasses the polymorphic site.

**Direct Sequence Context Around rs352139 (G1174A):**

The critical region surrounding the rs352139 polymorphism within intron 1 of the TLR9 gene is:

text

5′-...TGG GAC TGT TCA TTC CAA AAC GGT AGA ATG AAT TGC AGC AAA GGT ****G****GA GGG GTG ATT GCG CCC TCT GGT GGC CAT GCC TGT GCC AAG CGG AGA AGG CTG GTT CAG GGT CGG GAG GGG AAG GCC TGT GGG TGC ATC CCT GCA GCA GGG AGC TGG GAG CCA CAG ACA GGA GAA...-3′

In this sequence, the bolded **G** represents the wild-type allele at rs352139. The variant allele (A) substitutes this guanine. The forward primer (5′-CTT CAG CTG GCT GTT GTC TC-3′) binds upstream of this region, and the reverse primer (5′-GGA GAG TGC TGT TTG TTG GAG-3′) binds downstream (reverse complement), encompassing the polymorphic site within the 135 bp amplicon.

**Revised Text in the Manuscript:** **Section 2.6 (Page 8, Lines 180–182):**

The reference sequence, genomic location (GRCh38), annotated region, primer sequences, restriction enzymes and the amplification product fragments sizes of the TLR2, TLR4, and TLR9 polymorphisms are shown in Table 1.

**Table 1.** Primer sequences, restriction enzymes and PCR product fragment sizes of the TLR gene polymorphisms.

| **Gene Polymorphism** | **Reference Sequence** | **Genomic location** | **Annotated region** | **Primer sequence** | **Restriction enzyme** | **PCR product size (bp)** |
| --- | --- | --- | --- | --- | --- | --- |
| TLR2 T597C (rs3804099) | NM_003264.5 | chr4:153,703,504 | Exon 3; coding/synonymous variant | F: 5′- CTT CTC TGG TGC CTT TGT TGG -3′  R: 5′- GAA ATG TGA TGG GGA GAG GG -3′ | TaiI | TT (286)  TC (286, 184, 102)  CC (184, 102) |
| TLR4 A896G  (rs4986790) | NM_138554.5 | chr9:117,713,024 | Exon 3; missense variant (Asp299Gly) | F: 5′GATTAGCATACT TA GAC TACTACCTCC ATG-3′  R: 5′-GATCAACTTC TGA AAA AGC T TCCCAC-3′ | NcoI | AA (249)  AG (249, 223, 26)  GG (223, 26) |
| TLR9 G1174A (rs352139) | NG_033933.1 | chr3:52,258,372 | Intron 1 | F: 5′-CTTCAGCTGGCTGT  TGT CTC-3′  R: 5′-GGAGAGTGCTG TTG TTG GAG-3′ | BstUI | GG (135)  GA (135, 108, 27)  AA (108, 27) |

**Reviewer Comment 5:**-The authors state in the figure legends of the supplementary raw agarose gel images that bands in DNA ladder lane are “labeled according to the manufacturer’s specifications.” However, the images appear to retain the same labeling as in the previous version. In particular, the ladder includes bands labeled 286, 223, and 249 bp, which I have not been able to identify in the commercially available DNA ladders I am familiar with. If these fragment sizes are indeed present in the DNA ladder used, please provide the manufacturer/vendor and catalog number of the ladder so that the stated band sizes can be independently verified. Otherwise, please revise the labeling of the ladder accordingly.

**Author’s Response:** Thank you for bringing this important detail to our attention. We apologize for the confusion caused by the layout and labeling of the supplementary figure.We have completely revised **S1 Fig** and **S2 Fig** in the Supplementary Information to remove any ambiguous labels on the ladder lane, clearly demarcating the 100 bp ladder markers on the left side of Lane M while pointing the 286, 223, and 249 bp, fragment annotations directly to the sample lanes (Lanes 1–10) on the right. The supplementary figure legends have been updated accordingly.

**Revised Text in the Manuscript: Supplementary figures S1 and S2**

**S1 Fig.** Representative 2% agarose gel electrophoresis showing PCR-RFLP products of the TLR2 T597C (rs3804099) polymorphism. Lane M: 100-bp DNA ladder. Lanes 1, 2, and 8: homozygous TT genotype, showing a single undigested PCR product of 286 bp. Lanes 3, 4, and 9: heterozygous TC genotype, showing three fragments of 286 bp, 184 bp, and 102 bp. Lanes 5, 6, and 10: homozygous CC genotype, showing two restriction fragments of 184 bp and 102 bp. Lane 7: negative (no-template) control, confirming the absence of contamination during PCR amplification.

**S2 Fig.** Representative 2% agarose gel electrophoresis showing PCR-RFLP products of the TLR4 A896G (rs4986790) polymorphism. Lane M: 100-bp DNA ladder. Lanes 8, 9, and 10: homozygous AA genotype, showing a single undigested PCR product of 249 bp. Lanes 1, 2, 3, and 4: heterozygous AG genotype, showing three fragments of 249 bp, 223 bp, and 26 bp. Lanes 5 and 6: homozygous GG genotype, showing two restriction fragments of 223 bp and 26 bp. Lane 7: negative (no-template) control, confirming the absence of contamination during PCR amplification.
