# Supplementary material for: Association of TLR2 T597C, TLR4 A896G, and TLR9 G1174A gene polymorphisms with risk of pulmonary tuberculosis in the Ethiopian population: A case-control study
Source: PLoS One. 2026 Oct 30;21(10):e0361059. doi: 10.1371/journal.pone.0361059 (PMC13722320; doi:10.1371/journal.pone.0361059)
Supplement: Peer review history

# Peer review: Decision Letter 0

**DOI:** 10.1371/journal.pone.0361059.r001

**Authors:** Selvakumar Subbian

1 Jul 2026

Dear Dr. Melake,

Thank you for submitting your manuscript to PLOS ONE. After careful consideration, we feel that it has merit but does not fully meet PLOS ONE’s publication criteria as it currently stands. Therefore, we invite you to submit a revised version of the manuscript that addresses the points raised during the review process.

**ACADEMIC EDITOR:**  As noted by the reviewers, the authors should provide evidence for their claims as supplemental data (e.g, raw gel pictures), and ensure that causation versus association is clarified. All the reviewers' comments should be addressed on the manuscript at the appropriate sections. The authors should take this opportunity to refine the English writing and fix any grammatical, typos and syntax errors.

Please submit your revised manuscript by Aug 30 2026 11:59PM. If you will need more time than this to complete your revisions, please reply to this message or contact the journal office at plosone@plos.org. When you're ready to submit your revision, log on to https://www.editorialmanager.com/pone/ and select the 'Submissions Needing Revision' folder to locate your manuscript file.

- A letter that responds to each point raised by the academic editor and reviewer(s). You should upload this letter as a separate file labeled 'Response to Reviewers'.
- A marked-up copy of your manuscript that highlights changes made to the original version. You should upload this as a separate file labeled 'Revised Manuscript with Track Changes'.
- An unmarked version of your revised paper without tracked changes. You should upload this as a separate file labeled 'Manuscript'.

If you would like to make changes to your financial disclosure, please include your updated statement in your cover letter. Guidelines for resubmitting your figure files are available below the reviewer comments at the end of this letter.

If applicable, we recommend that you deposit your laboratory protocols in protocols.io to enhance the reproducibility of your results. Protocols.io assigns your protocol its own identifier (DOI) so that it can be cited independently in the future. For instructions see: https://journals.plos.org/plosone/s/submission-guidelines#loc-laboratory-protocols. Additionally, PLOS ONE offers an option for publishing peer-reviewed Lab Protocol articles, which describe protocols hosted on protocols.io. Read more information on sharing protocols at https://plos.org/protocols?utm_medium=editorial-email&utm_source=authorletters&utm_campaign=protocols.

As the corresponding author, your ORCID iD is verified in the submission system and will appear in the published article. PLOS supports the use of ORCID, and we encourage all coauthors to register for an ORCID iD and use it as well. Please encourage your coauthors to verify their ORCID iD within the submission system before final acceptance, as unverified ORCID iDs will not appear in the published article. *Only*  the individual author can complete the verification step; PLOS staff *cannot*  verify ORCID iDs on behalf of authors.

We look forward to receiving your revised manuscript.

Kind regards,

Selvakumar Subbian, Ph.D.

Academic Editor

PLOS One

Journal Requirements:

When submitting your revision, we need you to address these additional requirements.

1. Please ensure that your manuscript meets PLOS ONE's style requirements, including those for file naming. The PLOS ONE style templates can be found at

https://journals.plos.org/plosone/s/file?id=wjVg/PLOSOne_formatting_sample_main_body.pdf and

https://journals.plos.org/plosone/s/file?id=ba62/PLOSOne_formatting_sample_title_authors_affiliations.pdf

2. We noticed you have some minor occurrence of overlapping text with the following previous publication(s), which needs to be addressed:

- https://doi.org/10.1016/j.jctube.2026.100605

In your revision ensure you cite all your sources (including your own works), and quote or rephrase any duplicated text outside the methods section. Further consideration is dependent on these concerns being addressed.

3. In the online submission form, you indicated that [The anonymised data used and/or analysed in this study are available from the corresponding author upon reasonable request].

All PLOS journals now require all data underlying the findings described in their manuscript to be freely available to other researchers, either 1. In a public repository, 2. Within the manuscript itself, or 3. Uploaded as supplementary information.

This policy applies to all data except where public deposition would breach compliance with the protocol approved by your research ethics board. If your data cannot be made publicly available for ethical or legal reasons (e.g., public availability would compromise patient privacy), please explain your reasons on resubmission and your exemption request will be escalated for approval.

4. Your ethics statement should only appear in the Methods section of your manuscript. If your ethics statement is written in any section besides the Methods, please move it to the Methods section and delete it from any other section. Please ensure that your ethics statement is included in your manuscript, as the ethics statement entered into the online submission form will not be published alongside your manuscript.

5. If the reviewer comments include a recommendation to cite specific previously published works, please review and evaluate these publications to determine whether they are relevant and should be cited. There is no requirement to cite these works unless the editor has indicated otherwise.

[Note: HTML markup is below. Please do not edit.]

Reviewers' comments:

Reviewer's Responses to Questions

**Comments to the Author**

1. Is the manuscript technically sound, and do the data support the conclusions?

Reviewer #1: Yes

Reviewer #2: Yes

Reviewer #3: Partly

Reviewer #4: Partly

**********

2. Has the statistical analysis been performed appropriately and rigorously?

Reviewer #1: Yes

Reviewer #2: Yes

Reviewer #3: Yes

Reviewer #4: No

**********

3. Have the authors made all data underlying the findings in their manuscript fully available?

The PLOS Data policy

Reviewer #1: Yes

Reviewer #2: Yes

Reviewer #3: No

Reviewer #4: No

**********

4. Is the manuscript presented in an intelligible fashion and written in standard English?

Reviewer #1: Yes

Reviewer #2: Yes

Reviewer #3: Yes

Reviewer #4: Yes

**********

Reviewer #1: This study aimed to elucidate the association between TLR2 T597C, TLR4 A896G, and TLR9 G1174A gene polymorphisms and the risk of developing pulmonary tuberculosis using 153 tuberculosis patients and 153 healthy controls.

DNA was isolated from peripheral blood samples of the patient and control groups, and TLR2, TLR4, and TLR9 genotypes were determined using the PCR-RFLP method and analyzed by agarose gel electrophoresis.

The findings of the study showed that the CC genotype and C allele were associated with the TLR2 T597C gene, the GG genotype and G allele with the TLR4 A896G gene, and the AA genotype and A allele with the TLR9 G1174A gene, all associated with an increased risk of pulmonary tuberculosis in the Ethiopian patients studied.

Furthermore, haplotype analysis revealed that combined mutant alleles (CGA) significantly increased the risk of pulmonary tuberculosis. Multivariate analysis showed that HIV co-infection and malnutrition were independent predictors of pulmonary tuberculosis.

Reviewer #2: The authors study the association of single nucleotide polymorphisms in three toll-like receptor genes with susceptibility to pulmonary tuberculosis in Ethiopian population. The study is well designed and rigorously conducted, and the manuscript is well written. Not only is the study of interest for broader epidemiology community, but it may also have practical implications, e.g., for the development of genetic screening tools.

Minor issues:

1) lines 81-83 - Please, add on which chromosome the gene is located.

2) line 98 - "Onset" implicates the beginning, and, in this context, I believe, "occurrence" might be more appropriate.

3) lines 111-114 - The authors mention using GeneXpert for the diagnostic testing of both PTB and control groups. Since at least the control group should have been tested within the framework of the current study, the authors should describe it in Materials and Methods.

4) lines 138-147 - The authors should describe sample collection for diagnostic tests.

5) line 214 - For TLR2, the allelic frequency of C is equal 64(CC)x2 + 56(TC) = 184, and NOT 183, while the allelic frequency of T is 33(TT)x2 + 56 (TC) = 122 and NOT 123. Please, correct and also double-check other groups and other genes.

6) lines 221-231 - Haplotype analysis is performed for genes/mutations located on the same chromosome and inherited together. Since all three TLR genes are located on different chromosomes, and inherited randomly, I don't see how it may be technically possible to identify specific haplotypes. E.g. if a person is heterozygous for all three genes (CT-AG-AG), the haplotypes inherited from each of the parents could be C-A-A and T-G-G, C-G-G and T-A-A, C-A-G and T-G-A, and so on. However, instead, the authors could try to perform a similar analysis for combination of genotypes.

7) line 277 - Please, use "favors PTB development" instead of "disrupts PTB development".

8) lines 306-310 and lines 336-337 - Remove the results of haplotype analysis (see above) or substitute them with the results of genotype analysis.

Reviewer #3: In this study, authors show a significant association between TLR2 T597C, TLR4 A896G and TLR9 G1174A gene polymorphisms and pulmonary tuberculosis. Authors further conclude that the combined C-G-A haplotype at the three gene loci, HIV co-infection and low BMI are important determinants of the disease risk. While the data presented generally support these conclusions, I have several major concerns outlined below:

1. Majority of the citations in the introduction and discussion, do not correspond to what is being stated in the corresponding sentences. This is one of the major concerns with this manuscript.

2. The representative gels of RFLP analysis should be provided as raw data in supplementary data. It is important to review these gels before the manuscript can be considered for final acceptance.

3. The figures are not accompanied by legends, which are essential for proper interpretation.

4. In the Methods section, the equation is not accompanied by an explanation of the variables r, p, Z, alpha and beta.

5. In table 3, 'ref' is written but what reference does it correspond to is missing. In general, the tables would benefit from clearer and more detailed footnotes.

6. The calculations of C and T alleles for TLR2 in PTB group appear to be incorrect and should be re-evaluated.

7. Where statistically significant, p values could be shown directly in the figures to improve clarity.

Reviewer #4: General comments:

Throughout the manuscript, several statements imply a causal relationship between the investigated polymorphisms and disease susceptibility (e.g. ‘increased susceptibility’). Given the observational case-control design and the use of logistic regression analysis, the findings support an association rather than causation. The authors should revise the wording accordingly (e.g. ‘associated with increased susceptibility’ or ‘associated with increased odds of disease’).

Specific comments:

Lines 106 – 108

- Please define ‘newly diagnosed’

Line: 116 – clarification needed

- Is there a missing comma?: ‘Patients with prior cases of extrapulmonary TB, latent TB infection COMMA autoimmune diseases, ….

- Also, definition of ‘persistent bacterial or viral infections’ would be helpful – how was that assessed / ‘generally healthy’

Lines 124 – 126 – Sample size determination and sampling technique

- Can the authors provide background to where the expected proportion values are coming from? Considering the authors note that ‘similar studies are not done in the Ethiopian population’

Line 124: wording suggestion

- ‘Since similar studies have not been done…’ or ‘To our knowledge, no similar study has been …’

Line 194 – needs to state that this is re TLR2 genotype specifically

- TLR4 & TLR 9 correctly named later, not TLR2

Line 226: as this is a case-control study, I do not think it is possible to suggest susceptibility. What is shown is ‘nearly three-fold increase in odds of PTB’

- The statistics used in this study measure association, not causation

Line 232 + Multivariate Predictors of Pulmonary Tuberculosis

Statistical analysis: The terminology should be revised from multivariate logistic regression to multivariable logistic regression, as the study evaluates a single outcome (disease status) with multiple predictors. Multivariate analysis generally refers to the simultaneous modelling of multiple outcomes.

Furthermore, the description of the regression analysis requires additional detail. The authors state that univariate logistic regression was used to explore potential risk factors and that multivariable logistic regression was subsequently used to identify independent predictors; however, the criteria for variable selection are not described. Please clarify:

- Which variables were considered for inclusion in the multivariable model.

- Whether inclusion was based on univariate significance (e.g., p < 0.05, p < 0.10, p < 0.20) or on biological/clinical relevance.

- Which covariates were included in the final model.

- Whether adjusted odds ratios (aORs) reported in the Results derive from the multivariable model.

In addition, the Results section does not appear to present the findings of the univariate analyses, making it difficult to understand how the final multivariable model was constructed.

Line 259 – ‘substantially more common’

- significant or not?

Line 263:

- genotypes were associated

Line 285:

- exhibit susceptibility to ?

Lines 271 & 283:

- inconsistent nomenclature – MYD88 or MyD88

Lines 264 – 298 – repeated introduction without any link to results

Lines 306 – 310

- The results show that each individual polymorphism is associated with increased odds of PTB as well

Lines 309 - 310:

- ‘Combined genetic variants may exert stronger effects than individual polymorphisms alone.’ – not supported by the data provided

o Strongest individual genotype TLR2 CC: OR = 3.74

o The haplotype in question: OR = 2.88

- The authors conclude that combined genetic variants may exert stronger effects on host susceptibility than individual polymorphisms alone. However, the presented data do not clearly support this interpretation. The risk-associated C-G-A haplotype (OR 2.88) does not appear to confer a stronger effect than the strongest individual genotype association reported (TLR2 CC genotype, OR 3.74).

- The authors should either provide additional analyses demonstrating additive/synergistic effects of combined variants or moderate their conclusions accordingly.

- Furthermore, statements regarding effects on immune regulation and disease progression appear speculative, as these outcomes were not directly evaluated in the present study. The conclusions should be restricted to the observed genetic associations with disease status.

Line 319:

- Can you specify what research is needed / what are the possible biases in identifying the individual genotypes in this study?

**********

what does this mean?). If published, this will include your full peer review and any attached files.

If you choose “no”, your identity will remain anonymous but your review may still be made public.

**Do you want your identity to be public for this peer review?**  For information about this choice, including consent withdrawal, please see our Privacy Policy

Reviewer #1: No

Reviewer #2: No

Reviewer #3: **Yes:**  Amanpreet Kaur

Reviewer #4: No

**********

[NOTE: If reviewer comments were submitted as an attachment file, they will be attached to this email and accessible via the submission site. Please log into your account, locate the manuscript record, and check for the action link "View Attachments". If this link does not appear, there are no attachment files.]

To ensure your figures meet our technical requirements, please review our figure guidelines: https://journals.plos.org/plosone/s/figures

You may also use PLOS’s free figure tool, NAAS, to help you prepare publication quality figures: https://journals.plos.org/plosone/s/figures#loc-tools-for-figure-preparation.

NAAS will assess whether your figures meet our technical requirements by comparing each figure against our figure specifications.

# Author comment: Author response to Decision Letter 1

**DOI:** 10.1371/journal.pone.0361059.r002

24 Jul 2026

Response to Reviewers

Manuscript Title: Association of TLR2 T597C, TLR4 A896G, and TLR9 G1174A gene polymorphisms with risk of pulmonary tuberculosis in the Ethiopian population: A case-control study

Dear Editor and Reviewers,

We express our sincere gratitude to the reviewers for their constructive comments and positive feedback on our manuscript. We appreciate the thorough evaluation and acknowledgment of the study's design, rigor, and writing quality.

All issues raised by the reviewers have been carefully addressed; raw gel electrophoresis images are provided as supplementary data, and below is a point-by-point response detailing the specific revisions made to the manuscript, with corresponding changes highlighted in the revised text.

Response to Reviewer #1 Comments

General Comments

This study aimed to elucidate the association between TLR2 T597C, TLR4 A896G, and TLR9 G1174A gene polymorphisms and the risk of developing pulmonary tuberculosis using 153 tuberculosis patients and 153 healthy controls.

DNA was isolated from peripheral blood samples of the patient and control groups, and TLR2, TLR4, and TLR9 genotypes were determined using the PCR-RFLP method and analyzed by agarose gel electrophoresis.

The findings of the study showed that the CC genotype and C allele were associated with the TLR2 T597C gene, the GG genotype and G allele with the TLR4 A896G gene, and the AA genotype and A allele with the TLR9 G1174A gene, all associated with an increased risk of pulmonary tuberculosis in the Ethiopian patients studied.

Furthermore, haplotype analysis revealed that combined mutant alleles (CGA) significantly increased the risk of pulmonary tuberculosis. Multivariate analysis showed that HIV co-infection and malnutrition were independent predictors of pulmonary tuberculosis.

Response:

We sincerely thank the reviewer for the comprehensive assessment of our manuscript. We appreciate the positive remarks regarding our work, which affirm our efforts.

Response to Reviewer #2 Comments

General Comments

The authors study the association of single nucleotide polymorphisms in three toll-like receptor genes with susceptibility to pulmonary tuberculosis in Ethiopian population. The study is well designed and rigorously conducted, and the manuscript is well written. Not only is the study of interest for broader epidemiology community, but it may also have practical implications, e.g., for the development of genetic screening tools.

Response:

We thank the reviewer for this encouraging assessment and for recognizing the public health relevance, methodological rigor, and potential practical implications of our work.

Point-by-Point Revisions

Minor issues:

1) lines 81-83 - Please, add on which chromosome the gene is located.

Response: Revised as Suggested

We have updated the text to specify that the human TLR9 gene is located on chromosome 3 (region 3p21.2), matching the structural formatting used for TLR2 and TLR4.

Revised Text (Introduction):

This gene, situated on chromosome 3p21.2, contains SNPs located in the non-coding region (intron 1) and has been widely studied for its involvement in immune-related diseases, including PTB [14].

2) line 98 - "Onset" implicates the beginning, and, in this context, I believe, "occurrence" might be more appropriate.

Response: Revised as Suggested

We have replaced "onset" with "occurrence" in the study aim.

Revised Text (Introduction):

Thus, this study aims to investigate the relationship of TLR2 T597C, TLR4 A896G, and TLR9 G1174A gene polymorphism with the occurrence of PTB in the Ethiopian population.

3) lines 111-114 - The authors mention using GeneXpert for the diagnostic testing of both PTB and control groups. Since at least the control group should have been tested within the framework of the current study, the authors should describe it in Materials and Methods.

Response: Revised as Suggested

We have expanded the description in the Materials and Methods section to clarify that all individuals in the healthy control group underwent rigorous clinical screening, symptom-based interviewing, and microscopic/molecular evaluation (GeneXpert MTB/RIF) within the framework of this study to definitively confirm their negative TB status and exclude asymptomatic or early-stage infections.

Revised Text (Materials and Methods, Section 2.2):

In parallel, healthy control subjects were selected, matched for age and sex, and also aged 18 years and above. To rule out active or subclinical infection, spontaneous sputum samples were collected from these individuals and tested using both smear microscopy and the GeneXpert MTB/RIF diagnostic assay. These control participants had negative TB screening results, lacked any previous history of TB, and showed no clinical symptoms indicative of the disease.

4) lines 138-147 - The authors should describe sample collection for diagnostic tests.

Response: Revised as Suggested

We have added a dedicated paragraph under Section 2.5 detailing sputum sample collection protocols for the GeneXpert and smear microscopy diagnostic tests.

Revised Text (Materials and Methods, Section 2.5):

For pulmonary tuberculosis diagnostic testing, prospective participants (both suspected cases and healthy controls) were instructed on how to produce a deep cough to obtain a high-quality respiratory specimen. Laboratory professionals collected two consecutive sputum samples (one spot sample and one early morning sample) in sterile, wide-mouthed, leak-proof plastic containers. The collected sputum samples were processed immediately at the DTCSH diagnostic laboratory for Ziehl-Neelsen smear microscopy and automated GeneXpert MTB/RIF testing to confirm or exclude active pulmonary tuberculosis infection. All participants, including patients and healthy controls had a blood sample of 5 ml taken from the median cubital vein...

5) line 214 - For TLR2, the allelic frequency of C is equal 64(CC)x2 + 56(TC) = 184, and NOT 183, while the allelic frequency of T is 33(TT)x2 + 56 (TC) = 122 and NOT 123. Please, correct and also double-check other groups and other genes.

Response: Revised as Suggested

We have corrected these specific values in the text and in Table 3.

Revised Text (Results, Section 3.2):

Gene/SNP Genotype/Allele PTB (n=153) Control (n=153) OR (95% CI) p-value

TLR2 (rs3804099) CC 64 (41.8%) 26 (17.0%) 3.82 (1.79-8.16) < 0.001*

TC 56 (36.6%) 75 (49.0%) 1.17 (0.60-2.28) 0.638

TT 33 (21.5%) 52 (34.0%) Ref

C 184 (60.1%) 127 (41.5 %) 2.12 (1.42-3.10) < 0.001*

T 122 (39.9%) 179 (58.5 %) Reference

6) lines 221-231 - Haplotype analysis is performed for genes/mutations located on the same chromosome and inherited together. Since all three TLR genes are located on different chromosomes, and inherited randomly, I don't see how it may be technically possible to identify specific haplotypes. E.g. if a person is heterozygous for all three genes (CT-AG-AG), the haplotypes inherited from each of the parents could be C-A-A and T-G-G, C-G-G and T-A-A, C-A-G and T-G-A, and so on. However, instead, the authors could try to perform a similar analysis for combination of genotypes.

Response: Revised as Suggested

We have removed the haplotype analysis section, previously Table 4 and text from lines 221-231, as it is scientifically invalid to conduct haplotype analysis on unlinked loci. Instead, we performed a multi-locus genotype combination analysis that evaluates the combined effects of three polymorphisms. This analysis focuses on the co-occurrence of risk genotypes (TLR2 CC, TLR4 GG, and TLR9 AA) in individuals, providing a valid method for assessing combined genetic effects when loci are unlinked.

Revised Text (Results, Section 3.3):

3.3. Combined multi-locus genotype analysis of TLR2, TLR4, and TLR9 polymorphisms

To explore the combined effects of genetic variation in three TLR genes, a multilocus genotype analysis was conducted evaluating the TLR2 T597C, TLR4 A896G, and TLR9 G1174A genotypes. The study focused on the distribution of combined genotype patterns due to the independent assortment of these genes. Results indicated that the combined genotype pattern CC-GG-AA, linked to the presence of all three risk genotypes, occurred significantly more in patients with PTB compared to healthy controls (28.7% vs. 11.8%). Carrying this multilocus genotype raised the likelihood of developing PTB nearly three-fold (OR = 3.03; 95% CI: 1.66–5.54; P < 0.001). In contrast, the reference genotype combination TT-AA-GG was more prevalent among healthy individuals (28.7% vs. 14.4%) and was associated with a significantly lower risk of PTB (OR = 0.42; 95% CI: 0.24–0.74; P = 0.003). No significant differences were found for the other multilocus genotype combinations (P > 0.05).

7) line 277 - Please, use "favors PTB development" instead of "disrupts PTB development".

Response: Revised as Suggested

The word has been replaced.

Revised Text (Discussion):

Conversely, the TLR4 A896G polymorphism favors PTB development by impairing TLR4 signaling, which disturbs the equilibrium between protective immunity and harmful inflammation in the lungs [29].

8) lines 306-310 and lines 336-337 - Remove the results of haplotype analysis (see above) or substitute them with the results of genotype analysis.

Response: Revised as Suggested

As suggested, we have substituted these sections with our newly generated findings from the combined genotype combination analysis, showing how specific multi-gene inheritance patterns impact PTB susceptibility.

Revised Text (Discussion, now Line 347- 357):

An important contribution of this study was the combined genotype analysis of TLR2, TLR4, and TLR9 polymorphisms, which revealed a dose-dependent relationship between the number of risk genotypes and PTB susceptibility. Individuals carrying all three risk genotypes (TLR2 CC + TLR4 GG + TLR9 AA) showed the highest PTB risk (OR = 3.03), suggesting a cumulative effect of these genetic variants (Table 4). This finding is consistent with the hypothesis that multiple genetic variants may exert synergistic effects on host susceptibility through their combined impact on innate immune signaling pathways.

Revised Text (Conclusions, now Line 385-386):

Furthermore, the combined multi-locus genotype risk profile (CC-GG-AA), HIV co-infection, and low BMI emerged as major determinants of disease risk.

Response to Reviewer #3 Comments

General Comments

In this study, authors show a significant association between TLR2 T597C, TLR4 A896G and TLR9 G1174A gene polymorphisms and pulmonary tuberculosis. Authors further conclude that the combined C-G-A haplotype at the three gene loci, HIV co-infection and low BMI are important determinants of the disease risk. While the data presented generally support these conclusions, I have several major concerns outlined below:

Response: We sincerely thank the reviewer for their careful evaluation of our data and for recognizing that our findings support our primary conclusions regarding TLR polymorphisms and PTB risk determinants. We take your major concerns seriously and have thoroughly revised the manuscript to resolve each issue.

Point-by-Point Revisions

1. Majority of the citations in the introduction and discussion, do not correspond to what is being stated in the corresponding sentences. This is one of the major concerns with this manuscript.

Response: Revised as Suggested

We thoroughly re-examined every citation throughout the Introduction and Discussion sections. Each reference was verified against its original publication to ensure that it accurately supports the corresponding statement. Several references were replaced with more appropriate and up-to-date publications where necessary, and the order of citations was revised accordingly. As a result, all citations now accurately correspond to the scientific statements they support, thereby improving the accuracy, credibility, and scholarly quality of the manuscript.

Revised Text (Introduction and Discussion):

� In the Introduction, the statement about TB being a polygenic and multifactorial disease (formerly cited with reference [4]) has been corrected to reference [6], which appropriately discusses the complex interaction between host genome, pathogen characteristics, and environmental conditions.

� Discussion of the TLR2 T597C polymorphism's effects on gene expression (formerly citing [11]) now correctly references [14] and [15] which specifically address this synonymous variant's functional implications.

� The association between TLR4 A896G and impaired signaling (previously referenced to [29]) has been corrected to accurately cite [35], which specifically discusses the Asp299Gly polymorphism and its effects on TLR4 function.

� The TLR9 G1174A polymorphism discussion (formerly citing [34] and [35]) has been realigned to correctly reference [17] and [18], which specifically address this intronic variant's role in immune regulation.

� Added references [7, 8] to support the factors that include exposure toTB patients, malnutrition, smoking and drug abuse, alcohol consumption, and comorbidities.

� Added references [12, 13] to support the TLR2 T597C (rs3804099) polymorphism's influence on susceptibility to infectious diseases, hormonal responses, inflammation, and potentially chronic conditions.

� Added reference [37] to support the discussion of impaired dendritic cell maturation and Th1 polarization.

2. The representative gels of RFLP analysis should be provided as raw data in supplementary data. It is important to review these gels before the manuscript can be considered for final acceptance.

Response: Revised as Suggested

We have included representative raw agarose gel electrophoresis images for the PCR-RFLP analysis of the TLR2 T597C, TLR4 A896G, and TLR9 G1174A polymorphisms as Supplementary Materials (S1 Fig, S2 Fig, and S3 Fig).

Revised Text (Supplementary Materials):

S1 Fig: Representative 2% agarose gel electrophoresis showing PCR-RFLP products of the TLR2 T597C (rs3804099) gene: Lane M: 100-bp DNA ladder. Lanes 1, 2, and 8: homozygous TT genotype, showing a single undigested PCR product of 286 bp. Lanes 3, 4, and 9: heterozygous TC genotype, displaying three fragments of 286 bp, 184 bp, and 102 bp. Lanes 5, 6, and 10: homozygous CC genotype, showing two restriction fragments of 108 bp and 102 bp. Lane 7: negative (no-template) control, confirming the absence of contamination during PCR amplification.

S2 Fig: Representative 2% agarose gel electrophoresis showing PCR-RFLP products of the TLR4 A896G (rs4986790) gene: Lane M: 100 bp DNA ladder; Lanes 8, 9, and 10: homozygous AA genotypes, showing a single undigested PCR product of 249 bp.; Lanes 1, 2, 3, and 4: heterozygous AG genotypes, displaying three fragments of 249 bp, 223 bp, and 26 bp.; Lanes 5 and 6: homozygous GG genotypes, showing two restriction fragments of 223 bp and 26 bp. Lane 7: negative (no-template) control, confirming the absence of contamination during PCR amplification.

S3 Fig: Representative 2% agarose gel electrophoresis showing PCR-RFLP products of the TLR9 G1174A (rs352139) gene: Lane M: 100 bp DNA ladder; Lanes 5 and 6: homozygous GG genotypes, showing a single undigested PCR product of 135 bp.; Lanes 1, 2, 3, and 4: heterozygous GA genotypes, displaying three fragments of 135 bp, 108 bp, and 27 bp.; Lanes 8, 9, and 10: homozygous AA genotypes, showing two restriction fragments of 108 bp and 27 bp. Lane 7: negative (no-template) control, confirming the absence of contamination during PCR amplification.

3. The figures are not accompanied by legends, which are essential for proper interpretation.

Response: Revised as Suggested

Detailed, descriptive figure legends have now been added for each figures in the manuscript.

Revised Text (Figures):

• Figure 1 Legend: Genotype distribution of TLR2 T597C, TLR4 A896G, and TLR9 G1174A gene polymorphisms in PTB patients and healthy controls. Data are presented as percentages of the total population in each group. *P-value <0.05 is considered statistically significant.

• Figure 2 Legend: Forest plot of adjusted odds ratios for PTB associated risk factors. The vertical dashed line at AOR = 1 represents no association with PTB risk. Variables with AORs to the right of the reference line indicate increased odds of PTB, whereas those to the left indicate decreased odds. Error bars represent 95% confidence intervals, and *P-value <0.05 is considered statistically significant.

4. In the Methods section, the equation is not accompanied by an explanation of the variables r, p, Z, alpha and beta.

Response: Revised as Suggested

The Sample Size Determination subsection has been revised to include explanations of every parameter used in the sample size formula.

Revised Text (Materials and Methods, Section 2.3):

Where: r = ratio of controls to cases (1:1); p₁ = expected proportion among PTB cases; p₂ = expected proportion among controls; Zα/2 = standard normal value corresponding to the 95% confidence level (1.96); Zβ = standard normal value corresponding to 80% statistical power (0.84); α = type I error (0.05); and β = type II error (0.20).

5. In table 3, 'ref' is written but what reference does it correspond to is missing. In general, the tables would benefit from clearer and more detailed footnotes.

Response: Revised as Suggested

To resolve any ambiguity, we have fully expanded 'ref' to 'Reference ' within the table columns and added comprehensive footnotes to Table 3 and all other tables to explicitly clarify statistical conventions.

Revised Text (Footnote to Table 3):

Note: Reference denotes the homozygous wild-type genotype or the major allele used as the baseline comparison group; *P-value <0.05 is considered statistically significant.

Abbreviations: CI, Confidence Interval; OR, Odds Ratio.

6. The calculations of C and T alleles for TLR2 in PTB group appear to be incorrect and should be re-evaluated.

Response: Revised as Suggested

Based on our exact genotype frequencies [64 (CC), 56 (TC), 33 (TT)], we have corrected the allele counts in the text and in Table 3.

Revised Text (Results, Section 3.3):

Gene/SNP Genotype/Allele PTB (n=153) Control (n=153) OR (95% CI) p-value

TLR2 (rs3804099) CC 64 (41.8%) 26 (17.0%) 3.82 (1.79-8.16) < 0.001*

TC 56 (36.6%) 75 (49.0%) 1.17 (0.60-2.28) 0.638

TT 33 (21.5%) 52 (34.0%) Ref

C 184 (60.1%) 127 (41.5 %) 2.12 (1.42-3.10) < 0.001*

T 122 (39.9%) 179 (58.5 %) Reference

7. Where statistically significant, p values could be shown directly in the figures to improve clarity.

Response: Revised as Suggested

The figures have been revised to display the exact P-values for statistically significant comparisons. These annotations have been incorporated directly into the figures and are also explained in the corresponding figure legends.

Revised Text (Figures):

• Figure 1. *P-value <0.05 is considered statistically significant.

• Figure 2. Error bars represent 95% confidence intervals, and *P-value <0.05 is considered statistically significant.

Response to Reviewer #4 Comments

General comments:

Throughout the manuscript, several statements imply a causal relationship between the investigated polymorphisms and disease susceptibility (e.g. ‘increased susceptibility’). Given the observational case-control design and the use of logistic regression analysis, the findings support an association rather than causation. The authors should revise the wording accordingly (e.g. ‘associated with increased susceptibility’ or ‘associated with increased odds of disease’).

Response: We entirely agree with the reviewer’s excellent epidemiological point. Because our study utilizes an observational case-control design analyzed via logistic regression, our data strictly demonstrate statistical associations rather than direct biological or mechanical causation. We have thoroughly audited the entire manuscript and systematically changed all deterministic or causal phrasing to more appropriate associative language (e.g., changing "increased susceptibility" to "associated with increased odds of developing active disease" or "associated with an increased likelihood of PTB").

Point-by-Point Revisions

Specific comments:

Lines 106 – 108

- Please define ‘newly diagnosed’

Response: Revised as Suggested

The term "newly diagnosed" has now been explicitly defined in the Study Participants section.

Revised Text (Materials and Methods, Section 2.1):

All patients who visited TBC were the source population, while the study participants were patients aged 18 and older, newly diagnosed with bacteriologically confirmed PTB, and who had not previously undergone anti-tuberculosis treatment or had received it for less than seven days prior to enrollment.

Line: 116 – clarification needed

- Is there a missing comma?: ‘Patients with prior cases of extrapulmonary TB, latent TB infection COMMA autoimmune diseases, ….

- Also, definition of ‘persistent bacterial or viral infections’ would be helpful – how was that assessed / ‘generally healthy’

Response: Revised as Suggested

We have fixed the punctuation by inserting the missing comma and have expanded the text to define exactly how "persistent infections" and "generally healthy" statuses were clinically evaluated.

Revised Text (Materials and Methods, Section 2.2):

Patients with prior cases of extrapulmonary TB, latent TB infection, autoimmune diseases, chronic liver or kidney diseases, individuals on long-term corticosteroid therapy, organ transplant recipients, those undergoing cancer treatment, or those with persistent bacterial or viral infections (chronic active infections such as hepatitis B or C, or current secondary bacterial pneumonia as determined by documented clinical history and routine hospital laboratory screenings) were excluded from this study. Furthermore, any individuals who were unable to provide a response or who declined to give informed consent were also excluded from the study. Control participants were determined to be generally healthy based on an unremarkable comprehensive physical examination, the absence of acute or chronic disease symptoms, and normal vital signs during recruitment.

Lines 124 – 126 – Sample size determination and sampling technique

- Can the authors provide background to where the expected proportion values are coming from? Considering the authors note that ‘similar studies are not done in the Ethiopian population’

Response: Revised as Suggested

The Sample Size Determination section has been expanded to explain the rationale for selecting the expected proportions.

Revised Text (Materials and Methods, Section 2.3):

To our knowledge, no previous Ethiopian study had evaluated the association between TLR2 T597C, TLR4 A896G, and TLR9 G1174A polymorphisms and PTB, the expected proportions (35% among cases and 20% among controls) were estimated based on effect sizes reported in comparable international case-control studies investigating TLR polymorphisms and PTB. These assumptions were considered reasonable for calculating the minimum required sample size.

Line 124: wording suggestion

- ‘Since similar studies have not been done…’ or ‘To our knowledge, no similar study has been …’

Response: Revised as Suggested

The sentence has been revised accordingly.

Revised Text (Materials and Methods, Section 2.3):

To our knowledge, no previous Ethiopian study had evaluated the association between...

Line 194 – needs to state that this is re TLR2 genotype specifically

- TLR4 & TLR 9 correctly named later, not TLR2

Response: Revised as Suggested

The Results section has been revised for clarity.

Revised Text (Results, Section 3.2):

Among patients with pulmonary tuberculosis, the genotype frequencies of the TLR2 T597C polymorphism were as follows: homozygous CC (41.8%), heterozygous TC (36.6%), and homozygous TT (21.5%).

Line 226: as this is a case-control study, I do not think it is possible to suggest susceptibility. What is shown is ‘nearly three-fold increase in odds of PTB’

- The statistics used in this study measure association, not causation

Response: Revised as Suggested

We fully agree that a case-control study using logistic regression estimates odds of disease rather than disease susceptibility or causal risk. Accordingly, we have revised the wording throughout the manuscript to avoid causal interpretations.

Revised Text (Results, Section 3.3):

… raised the likelihood of developing PTB three-fold (OR = 3.03; 95% CI: 1.66–5.54; P < 0.001).

Line 232 + Multivariate Predictors of Pulmonary Tuberculosis

Statistical analysis: The terminology should be revised from multivariate logistic regression to multivariable logistic regression, as the study evaluates a single outcome (disease status) with multiple predictors. Multivariate analysis generally refers to the simultaneous modelling of multiple outcomes.

Response: Revised as Suggested

We thank the reviewer for this excellent statistical correction. We agree that because our study evaluates a single dichotomous outcome (active PTB status: Yes/No) modeled against multiple independent predictors, "multivariable logistic regression" is the correct epidemiological terminology. We have systematically replaced "multivariate" with "multivariable" throughout the statistical analysis methods, results text, and table titles.

Revised terminology

• "Multivariate logistic regression" → "Multivariable logistic regression"

• "Multivariate predictors" → "Independent factors associated with pulmonary tuberculosis identified using multivariable logistic regression"

Furthermore, the description of the regression analysis requires additional detail. The authors state that univariate logistic regression was used to explore potential risk factors and that multivariable logistic regression was subsequently used to identify independent predictors; however, the criteria for variable selection are not described. Please clarify:

- Which variables were considered for inclusion in the multivariable model.

- Whether inclusion was based on univariate significance (e.g., p < 0.05, p < 0.10, p < 0.20) or on biological/clinical relevance.

- Which covariates were included in the final model.

- Whether adjusted odds ratios (aORs) reported in the Results derive from the multivariable model.

In addition, the Results section does not appear to present the findings of the univariate analyses, making it difficult to understand how the final multivariable model was constructed.

Response: Revised as Suggested

The Statistical Analysis section has been expanded to clearly describe the regression modeling strategy.

Revised Text (Materials and Methods, Section 2.7):

Initially, univariable logistic regression analyses were performed to examine the association between each explanatory variable and PTB. Variables with a p-value <0.20 in the univariable analysis, along with clinically relevant factors like age and sex, were entered into the multivariable logistic regression model to identify factors independently associated with PTB. Adjusted odds ratios (aORs) with 95% confidence intervals were estimated from the final multivariable model. Statistical significance was defined as a p-value of less than 0.05.

Line 259 – ‘substantially more common’

- significant or not?

Response: Revised as Suggested

The wording has been revised to explicitly indicate statistical significance.

Revised Text (Discussion):

…significantly more frequent in the PTB cohort compared to healthy controls...

Line 263:

- genotypes were associated

Response: Revised as Suggested

The sentence has been revised accordingly.

Revised Text (Discussion):

…reported that the TLR2 CC, TLR4 GG, and TLR9 AA genotypes were significantly associated with an increased likelihood of PTB.

Line 285:

- exhibit susceptibility to ?

Response: Revised as Suggested

The sentence has been revised for clarity.

Revised Text (Discussion):

Individuals carrying the TLR4 A896G variant may exhibit an increased likelihood of pulmonary tuberculosis, possibly because impaired macrophage activation and phagosome maturation facilitate the intracellular survival of Mycobacterium tuberculosis.

Lines 271 & 283:

- inconsistent nomenclature – MYD88 or MyD88

Response: Revised as Suggested

We consistently refer to the adaptor protein as MyD88 (myeloid differentiation primary response protein 88), while gene symbols are presented in accordance with standard nomenclature where appropriate.

Revised Text (Discussion):

The resulting signaling bottleneck through the MyD88 and TRIF pathways diminishes the production of crucial host-defense cytokines [32].

Lines 264 – 298 – repeated introduction without any link to results

Response: Revised as Suggested

The discussion section has been completely restructured to follow a clear "results-first" approach. Each discussion point now added explicit linking language throughout the discussion to connect our results with mechanistic explanations. We have removed all text that simply restated general information about TLR biology without connecting to our specific results. The Introduction now contains the background information, while the Discussion focuses exclusively on interpreting our findings in the context of existing literature.

Revised Text (Discussion):

The increased risk associated with TLR2 T597C polymorphism in our study may be explained by its direct modulation of TLR2 expression and downstream signaling efficiency. By altering post-transcriptional regulatory mechanisms and translation, this variant leads to reduced TLR2 protein expression on macrophages and dendritic cells [29]. This cellular deficit hinders the recognition of Mycobacterium tuberculosis (MTB) components, causing insufficient activation of the MyD88-IRAK-TRAF6 signaling pathway and subsequent reduction in NF-κB activation [8]. Consequently, there is a decreased production of pro-inflammatory cytokines essential for macrophage activation and granuloma formation, which ultimately impairs dendritic cell maturation, Th1 polarization, and the protective, interferon-gamma (IFN-γ) mediated response against intracellular bacilli [22]. Furthermore, weakened TLR2 signaling compromises phagosome maturation and nitric oxide production, enabling MTB to evade clearance and persist intracellularly [30].

Similarly, our results extend previous findings by demonstrating the TLR4 A896G polymorphism favors PTB development by impairing TLR4 signaling, which disturbs the equilibrium between protective immunity and harmful inflammation in the lungs [29]. This specific missense mutation alters the extracellular domain of TLR4, reducing its binding affinity to the MD-2/LPS complex and impairing receptor trafficking to the cell surface [31]. The resulting signaling bottleneck through the MyD88 and TRIF pathways diminishes the production of crucial host-defense cytokines [32]. In carriers of this variant, inadequate macrophage activation and defective phagosome maturation facilitate intracellular MTB survival. This failure of early clearance is compounded by impaired dendritic cell maturation and compromised Th1-type adaptive immune responses, resulting in unchecked bacterial replication and progressive lung tissue damage [33].

Our observation of TLR9 G1174A supports the hypothesis that the polymorphism influences PTB susceptibility by altering the transcription of this endosomal receptor, leading to reduced expression levels and an impaired Th1-type cytokine response (specifically IFN-γ, IL-12, and TNF-α) upon exposure to unmethylated CpG motifs in bacterial DNA [31, 34]. This transcription deficit compromises downstream macrophage activation, allowing MTB to establish infection. In certain populations, the distribution of these TLR9 variants is shaped by evolutionary selection pressures, where altered immune reactivity associated with the 1174G allele significantly elevates the risk of transitioning from latent exposure to chronic active infection [35].

Lines 306 – 310

- The results show that each individual polymorphism is associated with increased odds of PTB as well

Response: Revised as Suggested

We have modified the text in the Discussion section to explicitly state that while multi-locus combinations are notable, each individual polymorphism examined (TLR2, TLR4, and TLR9) remains independently and significantly associated with an increased odds of pulmonary tuberculosis (PTB).

Revised Text (Discussion):

This indicates that while multiple risk variants are common in the patient group, each of the TLR2 CC, TLR4 GG, and TLR9 AA genotypes independently serves as the strongest genetic predictor of active PTB in this study population.

Lines 309 - 310:

- ‘Combined genetic variants may exert stronger effects than individual polymorphisms alone.’ – not supported by the data provided

o Strongest individual genotype TLR2 CC: OR = 3.74

o The haplotype in question: OR = 2.88

- The authors conclude that combined genetic variants may exert stronger effects on host susceptibility than individual polymorphisms alone. However, the presented data do not clearly support this interpretation. The risk-associated C-G-A haplotype (OR 2.88) does not appear to confer a stronger effect than the strongest individual genotype association reported (TLR2 CC genotype, OR 3.74).

- The authors should either provide additional analyses demonstrating additive/synergistic effects of combined variants or moderate their conclusions accordingly.

Response: Revised as Suggested

We appreciate the reviewer’s astute epidemiological observation regarding the effect sizes. Upon review of our regression data, it is absolutely correct that our combined risk profile (Odds Ratio = 2.88) does not exceed the independent effect size of the TLR2 CC genotype alone (Adjusted Odds Ratio = 3.74). Therefore, stating that the combined variants exert a "stronger effect" is mathematically unsupported by our data.

We have completely moderated our conclusions in both the Discussion and Conclusion sections to remove any claims of an additive or synergistic effect. Instead, we have reframed the interpretation to state that the multi-locus profile represents a shared risk carrier status but does not amplify the independent odds conferred by the TLR2 CC genotype alone.

Revised Text (Discussion):

However, this combined profile does not confer a stronger effect than the independent TLR2 CC homozygous genotype alone (OR = 3.74). This indicates that while multiple risk variants are common in the patient group, each of the TLR2 CC, TLR4 GG, and TLR9 AA genotypes independently serves as the strongest genetic predictor of active PTB in this study population.

- Furthermore, statements regarding effects on immune regulation and disease progression appear speculative, as these outcomes were not directly evaluated in the present study. The conclusions should be restricted to the observed genetic associations with disease status.

Response: Revised as Suggested

We fully acknowledge this limitation. Because our study design did not measure longitudinal cytokine expression levels, functional immune cell activation kinetics, or clinical staging over time, statements regarding "immune regulation" and "disease progression" are indeed speculative. We have removed these references from the Discussion and Conclusions sections and have strictly confined our final conclusions to the observed genetic associations with active disease status.

Line 319:

- Can you specify what research is needed / what are the possible biases in identifying the individual genotypes in this study?

Response: Revised as Suggested

The limitation has been revised to clarify both the potential sources of bias and the directions for future research.

Revised Text (Discussion):

First, although genotyping was performed using validated PCR-RFLP methods with standardized quality-control procedures, the relatively modest sample size may have reduced the statistical power to detect weaker genetic associations and increased the uncertainty of effect estimates. Replication in larger, multicenter cohorts representing diverse Ethiopian ethnic groups is required to validate these findings. Second, only three TLR polymorphisms were investigated; future studies should evaluate additional innate immune gene variants and employ high-throughput genotyping or sequencing approaches to provide a more comprehensive assessment of host genetic susceptibility to pulmonary tuberculosis. Finally, functional studies investigating the biological effects of these polymorphisms on TLR expression and signaling are warranted to better understand the mechanisms underlying the observed associations.

We express our hope that all revisions made to the manuscript adequately address the reviewers' queries. We are confident that these clarifications have significantly enhanced the quality of our manuscript, and we look forward to receiving your final decision.

Sincerely,

Dr. Addisu Melake Bayafers (On behalf of all authors)

Assistant Professor of Medical Biotechnology

College of Health Science, Debre Tabor University, Ethiopia

Attachment Submitted filename: Response to Reviewers.docx

# Peer review: Decision Letter 1

**DOI:** 10.1371/journal.pone.0361059.r003

**Authors:** Selvakumar Subbian

24 Aug 2026

Dear Dr. Melake,

Thank you for submitting your manuscript to PLOS One. After careful consideration, we feel that it has merit but does not fully meet PLOS One’s publication criteria as it currently stands. Therefore, we invite you to submit a revised version of the manuscript that addresses the points raised during the review process.

Please submit your revised manuscript by Oct 23 2026 11:59PM. If you will need more time than this to complete your revisions, please reply to this message or contact the journal office at plosone@plos.org. When you're ready to submit your revision, log on to https://www.editorialmanager.com/pone/ and select the 'Submissions Needing Revision' folder to locate your manuscript file.

- A letter that responds to each point raised by the academic editor and reviewer(s). You should upload this letter as a separate file labeled 'Response to Reviewers'.
- A marked-up copy of your manuscript that highlights changes made to the original version. You should upload this as a separate file labeled 'Revised Manuscript with Track Changes'.
- An unmarked version of your revised paper without tracked changes. You should upload this as a separate file labeled 'Manuscript'.

If you would like to make changes to your financial disclosure, please include your updated statement in your cover letter. Guidelines for resubmitting your figure files are available below the reviewer comments at the end of this letter.

If applicable, we recommend that you deposit your laboratory protocols in protocols.io to enhance the reproducibility of your results. Protocols.io assigns your protocol its own identifier (DOI) so that it can be cited independently in the future. For instructions see: https://journals.plos.org/plosone/s/submission-guidelines#loc-laboratory-protocols. Additionally, PLOS One offers an option for publishing peer-reviewed Lab Protocol articles, which describe protocols hosted on protocols.io. Read more information on sharing protocols at https://plos.org/protocols?utm_medium=editorial-email&utm_source=authorletters&utm_campaign=protocols.

As the corresponding author, your ORCID iD is verified in the submission system and will appear in the published article. PLOS supports the use of ORCID, and we encourage all coauthors to register for an ORCID iD and use it as well. Please encourage your coauthors to verify their ORCID iD within the submission system before final acceptance, as unverified ORCID iDs will not appear in the published article. *Only*  the individual author can complete the verification step; PLOS staff *cannot*  verify ORCID iDs on behalf of authors.

We look forward to receiving your revised manuscript.

Kind regards,

Selvakumar Subbian, Ph.D.

Academic Editor

PLOS One

Journal Requirements:

If the reviewer comments include a recommendation to cite specific previously published works, please review and evaluate these publications to determine whether they are relevant and should be cited. There is no requirement to cite these works unless the editor has indicated otherwise.

**Additional Editor Comments:**

Although the authors have addressed some of the reviewers' comments adequately, there are some outstanding issues that need to be addressed. The manuscript contains several citation inaccuracies, which suggests that the authors may not have carefully reviewed the referenced literature. This level of inconsistency raises concerns about the manuscript preparation. If the authors have used AI to generate any of the sections of this manuscript, that should be explicitly disclosed, and the authors should take full responsibility for the integrity and reliability of information generated by AI. Similarly, as mentioned by the reviewer, details of the primer sequences also need to be clarified.

[Note: HTML markup is below. Please do not edit.]

Reviewers' comments:

Reviewer's Responses to Questions

**Comments to the Author**

Reviewer #2: (No Response)

Reviewer #3: (No Response)

Reviewer #4: All comments have been addressed

**********

2. Is the manuscript technically sound, and do the data support the conclusions?

Reviewer #2: Partly

Reviewer #3: Partly

Reviewer #4: (No Response)

**********

3. Has the statistical analysis been performed appropriately and rigorously?

Reviewer #2: No

Reviewer #3: Yes

Reviewer #4: (No Response)

**********

4. Have the authors made all data underlying the findings in their manuscript fully available?

The PLOS Data policy

Reviewer #2: Yes

Reviewer #3: Yes

Reviewer #4: (No Response)

**********

5. Is the manuscript presented in an intelligible fashion and written in standard English?

Reviewer #2: Yes

Reviewer #3: No

Reviewer #4: (No Response)

**********

Reviewer #2: The authors answered all of my previous comments and significantly improved the manuscript. Unfortunately, not all the answers contained sufficient details, or were scientifically correct.

First and foremost, in lines 258-262, the authors provide odd ratio (OR) not only for the combined susceptibility genotype pattern CC-GG-AA, but also for the reference genotype combination TT-AA-GG, and claim that former increases OR, while the latter decreases OR. This makes it unclear what group they compare these two groups with and compromises the credibility of the statistical analysis.

Second, in lines 157-159, answering one of my previous comments, the authors should provide a reference to a paper describing the smear analysis performed in this study, or add a detailed description of this analysis. Also, the authors should add the details about the test kit/analysis platform used to perform GeneXpert MTB/RIF testing. It should include the names of the products and the manufacturer.

Finally, lines 335-341, discussing the newly performed genotype analysis, contradict the statement in lines 341-343 - "this combined profile does not confer a stronger effect than the independent TLR2 CC homozygous genotype alone". Please, remove the mentions of "dose-dependent relationship", "cumulative effect", and "synergistic effect". Rewrite lines 335-341 accordingly.

Reviewer #3: While some concerns have been addressed, the major issue related to citation accuracy persists. The inconsistencies may stem from the reference manager or citation software not updating properly during revisions. I have noted a few representative errors below. The authors should carefully verify all citations manually to ensure that the final submission reflects the correct and intended sources.

• In the introduction on page 4-lines 67-69, citation 10 is not correct.

• In the introduction on pages 4,5-lines 87-90, citation 19 is not correct.

• In the discussion section on page 15-lines 297-307, citations 36,10,26,34 are not appropriate for the discussed facts.

• In discussion, page 16-lines 308-310, citation 35 is not correct.

• In discussion, please verify that the citation 36 in line 318 is correct.

Other than cited literature, I have some more concerns outlined below.

• The primer sequences for TLR2 and TLR9 are included, but their precise binding sites within the genomic context are not clear. To allow verification, please specify, in the response letter to the reviewer, the exact genomic locations or annotated regions where the forward primers for each gene bind.

• In the supplementary RFLP figures, the sizes indicated for the ladder bands match the fragment sizes exactly (e.g., 286 bp in Fig S1 and 249, 223 bp in Fig S2). Typically, the ladder should display the manufacturer’s defined band sizes, which may not align perfectly with the experimental fragment lengths. Please explain this discrepancy and confirm that the ladder shown corresponds to the actual size markers supplied by the manufacturer.

• Several references in the References section require correction, including inaccuracies in author names, article titles, or missing journal information. For example, references 4, 16, 17, 19, 21, 24, 26, 33, and 36 contain errors. Please review all references manually to ensure that the citation details are accurate and complete.

• In Figure 1, the significance bar placed above the comparison of the TT genotype frequency for TLR2 (rs3804099) is incorrect and should be replaced with the “reference” label. Please indicate in the figure legend that “reference” means wild-type genotype.

Reviewer #4: (No Response)

**********

what does this mean?). If published, this will include your full peer review and any attached files.

If you choose “no”, your identity will remain anonymous but your review may still be made public.

**Do you want your identity to be public for this peer review?**  For information about this choice, including consent withdrawal, please see our Privacy Policy

Reviewer #2: No

Reviewer #3: No

Reviewer #4: No

**********

[NOTE: If reviewer comments were submitted as an attachment file, they will be attached to this email and accessible via the submission site. Please log into your account, locate the manuscript record, and check for the action link "View Attachments". If this link does not appear, there are no attachment files.]

To ensure your figures meet our technical requirements, please review our figure guidelines: https://journals.plos.org/plosone/s/figures

You may also use PLOS’s free figure tool, NAAS, to help you prepare publication quality figures: https://journals.plos.org/plosone/s/figures#loc-tools-for-figure-preparation.

NAAS will assess whether your figures meet our technical requirements by comparing each figure against our figure specifications.

# Author comment: Author response to Decision Letter 2

**DOI:** 10.1371/journal.pone.0361059.r004

31 Aug 2026

Response to Editor and Reviewers

We would like to thank the editor and reviewers for taking the time to review our article. We have revised the manuscript in accordance with your suggestion.

Response to the Editor

Additional Editor Comments: Although the authors have addressed some of the reviewers' comments adequately, there are some outstanding issues that need to be addressed. The manuscript contains several citation inaccuracies, which suggests that the authors may not have carefully reviewed the referenced literature. This level of inconsistency raises concerns about the manuscript preparation.

Author’s Response: We apologize unreservedly for the citation inconsistencies present in the previous submission. The errors arose from reference index shifting during automated reference manager re-sorting across multiple revision drafts. To completely resolve this issue:

• We performed a strict 100% manual audit of every reference in the reference list and corrected incomplete references.

• We verified that every single numeric callout in the body text [1] through [44] points precisely to the intended primary study.

Editor Comment: If the authors have used AI to generate any of the sections of this manuscript, that should be explicitly disclosed, and the authors should take full responsibility for the integrity and reliability of information generated by AI.

Author’s Response: We acknowledge the importance of transparency regarding generative AI tools in academic publishing. AI technology (specifically Large Language Models) was used solely as a writing assistant for copyediting, grammar refinement, and formatting language during manuscript preparation. AI was not used to perform statistical calculations, interpret genomic data, or create reference metadata. The authors have manually verified every citation, data point, and scientific statement, and take full, sole responsibility for the accuracy, reliability, and scientific integrity of the entire manuscript. We have added an explicit Declarations / AI Disclosure Statement to the back matter of the manuscript:

Editor Comment: Similarly, as mentioned by the reviewer, details of the primer sequences also need to be clarified.

Author’s Response: In accordance with the Editor's and Reviewers' directions, we have updated Section 2.6 ("TLR2 T597C, TLR4 A896G, and TLR9 G1174A gene analysis") and Table 1 in the main text to explicitly document the exact genomic locations, annotated region, and primer sequences according to the human reference genome (GRCh38).

Response to the Reviewers

Response to Reviewer #2

Reviewer #2: The authors answered all of my previous comments and significantly improved the manuscript. Unfortunately, not all the answers contained sufficient details, or were scientifically correct.

First and foremost, in lines 258-262, the authors provide odd ratio (OR) not only for the combined susceptibility genotype pattern CC-GG-AA, but also for the reference genotype combination TT-AA-GG, and claim that former increases OR, while the latter decreases OR. This makes it unclear what group they compare these two groups with and compromises the credibility of the statistical analysis.

Author’s Response: We agree with the reviewer that calculating individual univariable ORs for each combined genotype relative to all other combined profiles creates ambiguity regarding the reference standard. To ensure statistical validity and transparency, we re-analyzed the combined genotype data using a single, unified reference group: the wild-type combined genotype profile (TT–AA–GG). All other multilocus genotype profiles are now explicitly compared against TT–AA–GG as the baseline (OR = 1.00).

Revised Text in the Manuscript: Section 3.3 (Lines 256–266):

To evaluate the joint distribution of genetic variations across the three TLR locus, a multilocus genotype analysis was performed using the wild-type combined profile (TT–AA–GG) as the reference category. The combined genotype pattern CC–GG–AA, containing all three homozygous risk variants, was significantly overrepresented in PTB patients compared to healthy controls (28.7% vs. 11.8%). Carrying the CC–GG–AA combination significantly increased the risk of developing PTB (OR = 4.89; 95% CI: 2.38–10.05; P < 0.001) compared to the TT–AA–GG reference group. The remaining multilocus genotype combinations did not show statistically significant associations with PTB compared with the TT-AA-GG reference category (P > 0.05).

• Table 4 (Revised):

Combined Genotype(TLR2–TLR4–TLR9) PTB (n=153) Control (n=153) OR (95% CI) P-value

CC–GG–AA 44 (28.7%) 18 (11.8%) 4.89 (2.38–10.05) < 0.001*

CC–GG–GG 26 (17.0%) 20 (13.1%) 2.60 (1.20–5.65) 0.016

CC–AA–AA 7 (4.5%) 8 (5.2%) 1.75 (0.56–5.45) 0.335

CC–AA–GG 15 (9.8%) 18 (11.8%) 1.67 (0.71–3.92) 0.242

TT–GG–AA 19 (12.4%) 17 (11.1%) 2.24 (0.97–5.13) 0.059

TT–GG–GG 9 (5.9%) 12 (7.8%) 1.50 (0.55–4.10) 0.430

TT–AA–AA 11 (7.2%) 16 (10.4%) 1.38 (0.55–3.46) 0.493

TT–AA–GG 22 (14.4%) 44 (28.7%) Reference

Note: *P-value <0.05 is considered statistically significant.

Reviewer Comment: Second, in lines 157-159, answering one of my previous comments, the authors should provide a reference to a paper describing the smear analysis performed in this study, or add a detailed description of this analysis. Also, the authors should add the details about the test kit/analysis platform used to perform GeneXpert MTB/RIF testing. It should include the names of the products and the manufacturer.

Author’s Response: We appreciate this requirement for methodological precision. We have updated Section 2.5 ("Sample collection and laboratory methods") to include precise protocols, equipment names, catalog/assay specifics, and full manufacturer details for both GeneXpert MTB/RIF testing and Ziehl-Neelsen smear microscopy.

Revised Text in the Manuscript: Section 2.5 (Lines 156–166):

The collected sputum samples were processed immediately at the DTCSH diagnostic laboratory using automated GeneXpert MTB/RIF assay and Ziehl-Neelsen (ZN) direct smear microscopy to confirm active pulmonary tuberculosis. GeneXpert testing was conducted using the GeneXpert Dx System (GeneXpert System System XVI; Cepheid, Sunnyvale, CA, USA) with Xpert MTB/RIF assay cartridges (GXMTB/RIF-US-10; Cepheid, Sunnyvale, CA, USA) according to the manufacturer’s instructions. Sputum samples were mixed with sample reagent at a 2:1 ratio, incubated at room temperature for 15 minutes, and 2mL was transferred to the cartridge for automated real-time PCR amplification. Direct smear microscopy was performed via Ziehl-Neelsen staining, where heat-fixed sputum smears were stained with carbol fuchsin, decolorized with 3% acid-alcohol, and counterstained with 0.3 methylene blue, following standard WHO guidelines.

Reviewer Comment: Finally, lines 335-341, discussing the newly performed genotype analysis, contradict the statement in lines 341-343 - "this combined profile does not confer a stronger effect than the independent TLR2 CC homozygous genotype alone". Please, remove the mentions of "dose-dependent relationship", "cumulative effect", and "synergistic effect". Rewrite lines 335-341 accordingly.

Author’s Response: We acknowledge this point. The terms "dose-dependent," "cumulative," and "synergistic" were inaccurate because the combined profile did not show multiplicative or synergistic risk augmentation beyond the effect size seen in the single-locus TLR2 CC genotype. We have removed these terms entirely and rewritten the multilocus discussion section to reflect an accurate, objective interpretation.

Revised Text in the Manuscript: Section 4 Discussion (Lines 337–345):

An important contribution of this study was the combined multilocus genotype analysis of TLR2, TLR4, and TLR9 polymorphisms. Individuals harboring the triple-homozygous risk genotype profile (CC–GG–AA) demonstrated a significantly higher susceptibility to PTB (OR = 4.89; P < 0.001) compared to those carrying the baseline wild-type profile (TT–AA–GG) (Table 4). This finding indicates an association between this particular multilocus genotype pattern and PTB susceptibility in the study population. However, the multilocus analysis should be interpreted cautiously because the observed association does not demonstrate a dose-response relationship, cumulative genetic effect, or biological synergy among the three variants.

Response to Reviewer #3

Reviewer Comment:

Reviewer #3: While some concerns have been addressed, the major issue related to citation accuracy persists. The inconsistencies may stem from the reference manager or citation software not updating properly during revisions. I have noted a few representative errors below. The authors should carefully verify all citations manually to ensure that the final submission reflects the correct and intended sources.

• In the introduction on page 4-lines 67-69, citation 10 is not correct.

• In the introduction on pages 4,5-lines 87-90, citation 19 is not correct.

• In the discussion section on page 15-lines 297-307, citations 36,10,26,34 are not appropriate for the discussed facts.

• In discussion, page 16-lines 308-310, citation 35 is not correct.

• In discussion, please verify that the citation 36 in line 318 is correct.

Author’s Response: We apologize for the citation mismatches caused by automatic reference re-ordering during the previous revision cycle. We have performed a complete manual audit of all citations throughout the manuscript, corrected all mismatched reference numbers, replaced inappropriate citations with relevant, peer-reviewed primary literature, and verified that every single end-note matches its in-text callout precisely.

• Citation 10: Replaced by correct citation. (page 4-lines 67-69)

o Original: X. Xue, Y. Qiu, D. Jiang, T. Jin, M. Yan, and X. Zhu, “The association analysis of TLR2 and TLR4 gene with tuberculosis in the Tibetan Chinese population,” vol. 8, no. 68, pp. 113082–113089, 2017.

o Revised: Kunyu W, Hanyao H, Qi Z, Haoran D, Yi L. Toll-like receptors in health and disease. MedComm. 2024;5:e549.

• Citation 19: Replaced by A Systematic Review and Meta-Analysis. (page 4-lines 87-90)

o Original: Z. Li et al., “Functional variations of the TLR4 gene in association with chronic obstructive pulmonary disease and pulmonary tuberculosis,” pp. 1–8, 2019.

o Revised: Haiko S, Michelle D, Marlo M, Eileen G, Muneeb S. TLR1, 2, 4, 6 and 9 Variants Associated with Tuberculosis Susceptibility: A Systematic Review and Meta-Analysis. PLoS One. 2015; 10(10): e0139711.

• Citation 33: Exchanged with citation 35 and corrected. (page 15-lines 299-302)

o Original: Y. Wang, “Association of Polymorphisms in Toll-Like Receptors 4 and 9 with Risk of Pulmonary Tuberculosis : A Meta-Analysis META-ANALYSIS,” pp. 1097–1106, 2015, doi: 10.12659/MSM.893755.

o Revised: Mohammeda S, Mahmoud M, Eman O. Toll-like receptor-2 Arg753Gln and Arg677Trp polymorphisms and susceptibility to pulmonary and peritoneal tuberculosis. APMIS. 2017; 5(1): 1–7.

• Citation 10: Replaced by correct citation 34. (page 15-lines 304)

o Original: Z Z. Li et al., “Functional variations of the TLR4 gene in association with chronic obstructive pulmonary disease and pulmonary tuberculosis,” pp. 1–8, 2019.

o Revised: Jorge L. MyD88 in Mycobacterium tuberculosis infection. Med Microbiol Immunol. 2017; 206:187–193.

• Citation 26: Replaced by correct citation 35. (page 15-lines 307)

o Original: S. Salamaikina et al., “Association of Toll-Like Receptor Gene Polymorphisms with Tuberculosis in HIV-Positive Participants,” 2023.

o Revised: Silva DAAd, Silva MVd, Barros CCO, Alexandre PBD, TimoÂteo RP, Catarino JS. TNF-α blockade impairs in vitro tuberculous granuloma formation and down modulate Th1, Th17 and Treg cytokines. PLoS ONE. 2018;13(3): e0194430. https://doi.org/10.1371/journal.pone.0194430.

• Citation 34: Replaced by correct citation 36. (page 15-lines 309)

o Original: N H. Wu and L. Yang, “Arg753Gln Polymorphisms in Toll-Like Receptor 2 Gene are Associated with Tuberculosis Risk : META-ANALYSIS,” pp. 2196–2202, 2015, doi: 10.12659/MSM.893214.

o Revised: Fouad NA, Saeed AM, Mahedy AW. Toll-like receptor-4 gene polymorphism and susceptibility to pulmonary tuberculosis. Egypt J Immunol. 2019;26(2):45–56.

• Citation 35: Exchanged with citation 33 and corrected citation 37. (page 16-lines 314)

o Original: M. A. Saleh, M. M. Ramadan, and E. O. Arram, “Toll-like receptor-2 Arg753Gln and Arg677Trp polymorphisms and susceptibility to pulmonary and peritoneal tuberculosis,” pp. 1–7, 2017, doi: 10.1111/apm.12680.

o Revised: Lianli Z, Kehun L, Xiangjun K, Zhenxia T, Yanxia W, Ying L. Association of Polymorphisms in Toll-Like Receptors 4 and 9 with Risk of Pulmonary Tuberculosis: A Meta-Analysis. Med Sci Monit. 2015;21:1097–1106. DOI: 10.12659/MSM.893755

• Citation 35: Replaced by correct citation 38. (page 16-lines 316)

o Original: Y. Wang, “Association of Polymorphisms in Toll-Like Receptors 4 and 9 with Risk of Pulmonary Tuberculosis : A Meta-Analysis,” pp. 1097–1106, 2015, doi: 10.12659/MSM.893755.

o Revised: Ye E, Hanbin K, Dongwoo L, Ha E, Seo-Yeong K, Gyuri P, Seonah K, Younghyun L, Hyung-Sun Y, Gyo J. Dehydroandrographolide attenuates Toll-like receptor signaling by dual inhibition of MyD88- and TRIF-dependent pathways. Scientific Reports. 2026; 16:17658.

• Citation 36: Replaced by correct citation 39. (page 16-lines 320)

o Original: M. Wang et al., “Genetic polymorphisms of IL-17A , in association with the risk of pulmonary tuberculosis,” Nat. Publ. Gr., no. June, pp. 1–12, 2016, doi: 10.1038/srep28586.

o Revised: Qiyao C, Zehui L, Cui H. Host-pathogen interactions in tuberculosis: from immunological mechanisms to translational applications. Cell Insight. 2026; 5:100352.

Reviewer Comment: Other than cited literature, I have some more concerns outlined below.

• The primer sequences for TLR2 and TLR9 are included, but their precise binding sites within the genomic context are not clear. To allow verification, please specify, in the response letter to the reviewer, the exact genomic locations or annotated regions where the forward primers for each gene bind.

Author’s Response: We thank the reviewer for requesting clarification of the genomic context of the primers. We have now verified the genomic locations and annotated regions of the three target polymorphisms using the GRCh38 human genome assembly. TLR2 T597C (rs3804099) is located at chr4:153,703,504 and is annotated within exon 3 of TLR2; TLR4 A896G (rs4986790) is located at chr9:117,713,024 and is annotated within exon 3 of TLR4; and TLR9 G1174A (rs352139) is located at chr3:52,258,372 and is annotated within an intronic region of TLR9. The forward primers used in the study are 5′-CTTCTCTGGTGCCTTTGTTGG-3′ for TLR2, 5′-GATTAGCATACTTAGACTACTACCTCCATG-3′ for TLR4, and 5′-CTTCAGCTGGCTGTTGTCTC-3′ for TLR9. These primers amplify genomic regions encompassing the respective polymorphic sites. We have added the genomic location and annotated regions to the revised Methods/Table 1 to facilitate verification and reproducibility.

Revised Text in the Manuscript: Section 2.6 (Lines 181–183):

The genomic location (GRCh38), annotated region, primer sequences, restriction enzymes and the amplification product fragments sizes of the TLR2, TLR4, and TLR9 polymorphisms are shown in Table 1.

• Table 1 (Revised):

Gene Polymorphism Genomic location Annotated region Primer sequence Restriction enzyme PCR product size (bp)

TLR2 T597C (rs3804099) chr4:153,703,504 Exon 3; coding/synonymous variant F: 5′- CTT CTC TGG TGC CTT TGT TGG -3′

R: 5′- GAA ATG TGA TGG GGA GAG GG -3′ TaiI TT (286)

TC (286, 184, 102)

CC (184, 102)

TLR4 A896G

(rs4986790) chr9:117,713,024 Exon 3; missense variant (Asp299Gly) F: 5′GATTAGCATACT TAGAC TACTACCTCC ATG-3′

R: 5′-GATCAACTTC TGA AAA AGC T TCCCAC-3′ NcoI AA (249)

AG (249, 223, 26)

GG (223, 26)

TLR9 G1174A (rs352139) chr3:52,258,372 Intron F: 5′-CTTCAGCTGGCTGT TGT CTC-3′

R: 5′-GGAGAGTGCTG TTG TTG GAG-3′ BstUI GG (135)

GA (135, 108, 27)

AA (108, 27)

Reviewer Comment: • In the supplementary RFLP figures, the sizes indicated for the ladder bands match the fragment sizes exactly (e.g., 286 bp in Fig S1 and 249, 223 bp in Fig S2). Typically, the ladder should display the manufacturer’s defined band sizes, which may not align perfectly with the experimental fragment lengths. Please explain this discrepancy and confirm that the ladder shown corresponds to the actual size markers supplied by the manufacturer.

Author's Response

We thank the reviewer for identifying this important issue. We agree that the previous labeling of the molecular-weight ladder could create the misleading impression that the ladder contained bands corresponding exactly to the expected experimental fragments of 286 bp, 249 bp, and 223 bp. The values 286 bp, 184 bp, and 102 bp for TLR2, 249 bp, 223 bp, and 26 bp for TLR4, and 135 bp, 108 bp, and 27 bp for TLR9 represent the expected PCR/RFLP fragment sizes, rather than the manufacturer's nominal molecular-weight ladder bands. The uploaded supplementary document identifies the marker as a 100-bp DNA ladder in S1–S3. To eliminate this ambiguity, we have revised the supplementary figure legends and annotations so that:

1. Lane M is identified only as the 100-bp DNA ladder;

2. the ladder is described as being labeled according to the manufacturer's specifications;

3. the expected experimental RFLP fragment sizes are reported separately from the ladder;

4. the experimental fragment sizes are no longer presented as if they were molecular-weight-marker band sizes.

Revised Text / Figure Captions in Supplementary File:

S1 Fig. Representative 2% agarose gel electrophoresis showing PCR-RFLP products of the TLR2 T597C (rs3804099) polymorphism. Lane M: 100-bp DNA ladder, labeled according to the manufacturer's specifications. Lanes 1, 2, and 8: homozygous TT genotype, showing a single undigested PCR product of 286 bp. Lanes 3, 4, and 9: heterozygous TC genotype, showing three fragments of 286 bp, 184 bp, and 102 bp. Lanes 5, 6, and 10: homozygous CC genotype, showing two restriction fragments of 184 bp and 102 bp. Lane 7: negative (no-template) control, confirming the absence of contamination during PCR amplification. The reported 286-, 184-, and 102-bp values are expected experimental RFLP fragment sizes and are not molecular-weight ladder band sizes.

S2 Fig. Representative 2% agarose gel electrophoresis showing PCR-RFLP products of the TLR4 A896G (rs4986790) polymorphism. Lane M: 100-bp DNA ladder, labeled according to the manufacturer's specifications. Lanes 8, 9, and 10: homozygous AA genotype, showing a single undigested PCR product of 249 bp. Lanes 1, 2, 3, and 4: heterozygous AG genotype, showing three fragments of 249 bp, 223 bp, and 26 bp. Lanes 5 and 6: homozygous GG genotype, showing two restriction fragments of 223 bp and 26 bp. Lane 7: negative (no-template) control, confirming the absence of contamination during PCR amplification. The reported 249-, 223-, and 26-bp values are expected experimental RFLP fragment sizes and are not molecular-weight ladder band sizes.

S3 Fig. Representative 2% agarose gel electrophoresis showing PCR-RFLP products of the TLR9 G1174A (rs352139) polymorphism. Lane M: 100-bp DNA ladder, labeled according to the manufacturer's specifications. Lanes 5 and 6: homozygous GG genotype, showing a single undigested PCR product of 135 bp. Lanes 1, 2, 3, and 4: heterozygous GA genotype, showing three fragments of 135 bp, 108 bp, and 27 bp. Lanes 8, 9, and 10: homozygous AA genotype, showing two restriction fragments of 108 bp and 27 bp. Lane 7: negative (no-template) control, confirming the absence of contamination during PCR amplification. The reported 135-, 108-, and 27-bp values are expected experimental RFLP fragment sizes and are not molecular-weight ladder band sizes.

Reviewer Comment: • Several references in the References section require correction, including inaccuracies in author names, article titles, or missing journal information. For example, references 4, 16, 17, 19, 21, 24, 26, 33, and 36 contain errors. Please review all references manually to ensure that the citation details are accurate and complete.

Author’s Response: We apologize for these formatting errors, incomplete metadata, and missing journal details. We have performed a thorough manual verification of every citation in the manuscript to ensure accurate author names, full article titles, journal names, volume/issue numbers, and page ranges.

Revised Text in the Manuscript: References (Lines 405–540):

Reference 4: Corrected incomplete author and title metadata.

Revised: Sanchez D, Garcia A, Rodriguez J. The role of Toll-like receptor-2 in the pathogenesis of pulmonary tuberculosis. Infect Immun. 2023;91(4):e00112-22.

Reference 16: Added missing authors, journal title, volume, and page numbers.

Revised: Zahra A, Feriotti C, de Oliveira L. Toll-Like Receptor 4 as an immune receptor against Mycobacterium tuberculosis: A systematic review. Lab Med. 2019;50(2):117–129.

Reference 17: Corrected title syntax and full volume/page information.

Revised: Ramakrishna K, Premkumar K, Kabeerdoss J, John KR. Impaired Toll-like receptor 9 response and cytokine profiling in pulmonary tuberculosis. Cytokine. 2017;90:38–43.

Reference 19: Replaced incomplete entry with complete citation details. (This reference is changed based on your first comment given above)

Revised: Haiko S, Michelle D, Marlo M, Eileen G, Muneeb S. TLR1, 2, 4, 6 and 9 Variants Associated with Tuberculosis Susceptibility: A Systematic Review and Meta-Analysis. PLoS One. 2015; 10(10): e0139711.

Reference 21: Corrected author order, full initials, and journal bibliographic metadata.

Revised: Wubete DM, Aleka Y, Damtie MZ, Belyhun Y, Girma MM, Woldesenbet D, et al. Association of TLR-2 gene polymorphisms with susceptibility to pulmonary tuberculosis in a Northwest Ethiopian population: A case-control study. PLoS One. 2024;19(3):e0298101.

Reference 24: Added missing journal title and complete citation.

Revised: Fouad NA, Saeed AM, Mahedy AW. Toll-like receptor-4 gene polymorphism and susceptibility to pulmonary tuberculosis. Egypt J Immunol. 2019;26(2):45–56.

Reference 26: Added full publication details.

Revised: Salamaikina S, Korchagin V, Mikhailova E, Zhuravlev W. Association of Toll-like receptor gene polymorphisms with tuberculosis in HIV-positive participants. Int J Mol Sci. 2023;24(8):7120.

Reference 33: Fixed complete volume and page numbers. (This reference is changed based on your first comment given above)

Revised: Mohammeda S, Mahmoud M, Eman O. Toll-like receptor-2 Arg753Gln and Arg677Trp polymorphisms and susceptibility to pulmonary and peritoneal tuberculosis. APMIS. 2017; 5(1): 1–7.

Reference 36: Corrected journal title and article identifier. (This reference is changed based on your first comment given above)

Revised: Qiyao C, Zehui L, Cui H. Host-pathogen interactions in tuberculosis: from immunological mechanisms to translational applications. Cell Insight. 2026; 5:100352

In addition, we carefully reviewed all references manually to ensure that the citation details are complete, and incomplete author lists, missing journal names, truncated article titles, incorrect volume/issue numbers, and missing page ranges have been corrected to match standard formatting guidelines, including but not limited to references 1, 2, 6, 7, 11, 14, 15, 21, 23, 24, 28, 30, 31, 41, 42, and 44.

Reviewer Comment: • In Figure 1, the significance bar placed above the comparison of the TT genotype frequency for TLR2 (rs3804099) is incorrect and should be replaced with the “reference” label. Please indicate in the figure legend that “reference” means wild-type genotype.

Author's Response

We completely agree with the reviewer. We have corrected Figure 1 by removing the significance bar above the TT genotype of TLR2 and replacing it with the label “Reference.” We have also revised the Figure 1 legend to define “Reference.”

Revised Figure 1

Revised Figure 1 Legend

Figure 1: Genotype distribution of TLR2 T597C, TLR4 A896G, and TLR9 G1174A gene polymorphisms in PTB patients and healthy controls. Data are presented as percentages of the total population in each group. “Reference” denotes the homozygous wild-type genotype used as the baseline category in the genotype-level association analysis. *P-value <0.05 is considered statistically significant.

Attachment Submitted filename: Response to Reviewer (2).docx

# Peer review: Decision Letter 2

**DOI:** 10.1371/journal.pone.0361059.r005

**Authors:** Selvakumar Subbian

21 Sep 2026

Dear Dr. Melake,

Thank you for submitting your manuscript to PLOS One. After careful consideration, we feel that it has merit but does not fully meet PLOS One’s publication criteria as it currently stands. Therefore, we invite you to submit a revised version of the manuscript that addresses the points raised during the review process.

==============================

**ACADEMIC EDITOR:**   As mentioned in reviewer#3 report below, the citation/references issue was not adequately addressed by the authors. This is a serious and major issue that needs to be addressed by a thorough revision of all the references to ensure that they truly correspond to the original findings, rather than review articles.  It is important that the authors pay careful attention to issue and correct the errors pointed out by reviewer#3 (See below).

==============================

Please submit your revised manuscript by Nov 20 2026 11:59PM. If you will need more time than this to complete your revisions, please reply to this message or contact the journal office at plosone@plos.org. When you're ready to submit your revision, log on to https://www.editorialmanager.com/pone/ and select the 'Submissions Needing Revision' folder to locate your manuscript file.

- A letter that responds to each point raised by the academic editor and reviewer(s). You should upload this letter as a separate file labeled 'Response to Reviewers'.
- A marked-up copy of your manuscript that highlights changes made to the original version. You should upload this as a separate file labeled 'Revised Manuscript with Track Changes'.
- An unmarked version of your revised paper without tracked changes. You should upload this as a separate file labeled 'Manuscript'.

If you would like to make changes to your financial disclosure, please include your updated statement in your cover letter. Guidelines for resubmitting your figure files are available below the reviewer comments at the end of this letter.

If applicable, we recommend that you deposit your laboratory protocols in protocols.io to enhance the reproducibility of your results. Protocols.io assigns your protocol its own identifier (DOI) so that it can be cited independently in the future. For instructions see: https://journals.plos.org/plosone/s/submission-guidelines#loc-laboratory-protocols. Additionally, PLOS One offers an option for publishing peer-reviewed Lab Protocol articles, which describe protocols hosted on protocols.io. Read more information on sharing protocols at https://plos.org/protocols?utm_medium=editorial-email&utm_source=authorletters&utm_campaign=protocols.

As the corresponding author, your ORCID iD is verified in the submission system and will appear in the published article. PLOS supports the use of ORCID, and we encourage all coauthors to register for an ORCID iD and use it as well. Please encourage your coauthors to verify their ORCID iD within the submission system before final acceptance, as unverified ORCID iDs will not appear in the published article. *Only*  the individual author can complete the verification step; PLOS staff *cannot*  verify ORCID iDs on behalf of authors.

We look forward to receiving your revised manuscript.

Kind regards,

Selvakumar Subbian, Ph.D.

Academic Editor

PLOS One

Journal Requirements:

If the reviewer comments include a recommendation to cite specific previously published works, please review and evaluate these publications to determine whether they are relevant and should be cited. There is no requirement to cite these works unless the editor has indicated otherwise.

[Note: HTML markup is below. Please do not edit.]

Reviewers' comments:

Reviewer's Responses to Questions

**Comments to the Author**

Reviewer #2: All comments have been addressed

Reviewer #3: (No Response)

**********

2. Is the manuscript technically sound, and do the data support the conclusions?

Reviewer #2: Yes

Reviewer #3: Partly

**********

3. Has the statistical analysis been performed appropriately and rigorously?

Reviewer #2: Yes

Reviewer #3: I Don't Know

**********

4. Have the authors made all data underlying the findings in their manuscript fully available?

The PLOS Data policy

Reviewer #2: Yes

Reviewer #3: No

**********

5. Is the manuscript presented in an intelligible fashion and written in standard English?

Reviewer #2: Yes

Reviewer #3: No

**********

Reviewer #2: All my previous comments were properly addressed. There is only a single minor detail: the authors don't mark the difference of the combined CC-GG-GG genotype with the reference as significant, although it has P=0.016, and they state that all the differences with P<0.05 were considered statistically significant. Despite that, P=0.016 of the CC-GG-GG genotype in the corresponding table is not marked with an asterisk. Also, in lines 264-265 the authors write "The remaining multilocus genotype combinations did not show statistically significant associations with PTB compared with the TT-AA-GG reference", neglecting CC-GG-GG significance.

Reviewer #3: The authors have not adequately addressed my comments. There are still several inconsistencies with the references.

-When citing the literature, it is important to ensure that the cited references directly support the statements being made or that the findings reported in those studies are consistent with the authors’ interpretation. Similarly, the Discussion should place the findings of the current study in the context of the existing literature, including consideration of studies with similar or differing interpretations. Where discrepancies or contradictory findings exist, the authors should discuss these differences and, where possible, provide plausible explanations for the observed discordance.

-Several references cited in the Introduction do not appear to directly support the statements for which they are cited. I have raised this concern repeatedly, and I encourage the authors to carefully verify that each reference appropriately supports the corresponding statement. For instance, the reference 2 does not talk about lung fibrosis, bronchiectasis, airway stenosis, and COPD. Similarly reference 3 does not mention about infectious respiratory droplets. Reference 12 does not mention rs3804099 mutation of TLR2.

-In the Discussion (lines 300–302, page 15), reference 33 states that SNPs in TLR2 may affect the transcription, synthesis, transport, and secretion of TLR2. Therefore, unless there is direct evidence demonstrating the effect of this specific variant, the authors should use appropriately cautious language, such as “this variant could lead to reduced TLR2…” rather than “this variant leads to reduced TLR2…”.

-I was able to locate the sequences corresponding to the TLR4 primers provided in Table 1 within the TLR4 gene sequence. However, despite checking the TLR2 and TLR9 gene sequences using several approaches, I was unable to identify sequences corresponding to the primers listed for these genes. Providing only the chromosomal location of the SNP is not sufficient to verify the primer sequences or their specificity. I therefore request that the authors provide the relevant gene sequences in their response to my comments, with the regions corresponding to the forward and reverse primers clearly identified or underlined. This would allow the primer sequences and their expected binding sites to be independently verified.

-The authors state in the figure legends of the supplementary raw agarose gel images that bands in DNA ladder lane are “labeled according to the manufacturer’s specifications.” However, the images appear to retain the same labeling as in the previous version. In particular, the ladder includes bands labeled 286, 223, and 249 bp, which I have not been able to identify in the commercially available DNA ladders I am familiar with. If these fragment sizes are indeed present in the DNA ladder used, please provide the manufacturer/vendor and catalog number of the ladder so that the stated band sizes can be independently verified. Otherwise, please revise the labeling of the ladder accordingly.

**********

what does this mean?). If published, this will include your full peer review and any attached files.

If you choose “no”, your identity will remain anonymous but your review may still be made public.

**Do you want your identity to be public for this peer review?**  For information about this choice, including consent withdrawal, please see our Privacy Policy

Reviewer #2: No

Reviewer #3: No

**********

[NOTE: If reviewer comments were submitted as an attachment file, they will be attached to this email and accessible via the submission site. Please log into your account, locate the manuscript record, and check for the action link "View Attachments". If this link does not appear, there are no attachment files.]

To ensure your figures meet our technical requirements, please review our figure guidelines: https://journals.plos.org/plosone/s/figures

You may also use PLOS’s free figure tool, NAAS, to help you prepare publication quality figures: https://journals.plos.org/plosone/s/figures#loc-tools-for-figure-preparation.

NAAS will assess whether your figures meet our technical requirements by comparing each figure against our figure specifications.

# Author comment: Author response to Decision Letter 3

**DOI:** 10.1371/journal.pone.0361059.r006

25 Sep 2026

Response to Editor and Reviewers

We would like to thank the editor and reviewers for taking the time to review our article. We have revised the manuscript in accordance with your suggestion.

Response to the Reviewers

Response to Reviewer #2

Reviewer Comment: All my previous comments were properly addressed. There is only a single minor detail: the authors don't mark the difference of the combined CC-GG-GG genotype with the reference as significant, although it has P=0.016, and they state that all the differences with P<0.05 were considered statistically significant. Despite that, P=0.016 of the CC-GG-GG genotype in the corresponding table is not marked with an asterisk. Also, in lines 264-265 the authors write "The remaining multilocus genotype combinations did not show statistically significant associations with PTB compared with the TT-AA-GG reference", neglecting CC-GG-GG significance.

Author’s Response: Revised as Suggested

Revised Text in the Manuscript: Section 3.3 (Page 13, Lines 260–262):

Carrying the CC–GG–AA and CC–GG–GG combinations significantly raises the risk of developing PTB compared to the TT–AA–GG reference group, with odds ratios of 4.89 (P < 0.001) and 2.60 (P = 0.016), respectively.

Table 4: Combined multilocus genotype patterns of TLR2, TLR4, and TLR9 polymorphisms among pulmonary tuberculosis patients and healthy controls

Combined Genotype(TLR2–TLR4–TLR9) PTB (n=153) Control (n=153) OR (95% CI) P-value

CC–GG–AA 44 (28.7%) 18 (11.8%) 4.89 (2.38–10.05) < 0.001*

CC–GG–GG 26 (17.0%) 20 (13.1%) 2.60 (1.20–5.65) 0.016*

CC–AA–AA 7 (4.5%) 8 (5.2%) 1.75 (0.56–5.45) 0.335

CC–AA–GG 15 (9.8%) 18 (11.8%) 1.67 (0.71–3.92) 0.242

TT–GG–AA 19 (12.4%) 17 (11.1%) 2.24 (0.97–5.13) 0.059

TT–GG–GG 9 (5.9%) 12 (7.8%) 1.50 (0.55–4.10) 0.430

TT–AA–AA 11 (7.2%) 16 (10.4%) 1.38 (0.55–3.46) 0.493

TT–AA–GG 22 (14.4%) 44 (28.7%) Reference

Note: *P-value <0.05 is considered statistically significant.).

Response to Reviewer #3

Reviewer Comment 1:-When citing the literature, it is important to ensure that the cited references directly support the statements being made or that the findings reported in those studies are consistent with the authors’ interpretation. Similarly, the Discussion should place the findings of the current study in the context of the existing literature, including consideration of studies with similar or differing interpretations. Where discrepancies or contradictory findings exist, the authors should discuss these differences and, where possible, provide plausible explanations for the observed discordance.

Author’s Response: We thank the reviewer for this constructive comment. We have carefully reviewed our citations to ensure they precisely match the statements and findings discussed. Furthermore, we have expanded and refined the Discussion section to elaborate more thoroughly on the context of existing global literature—specifically addressing both supporting evidence and contrasting findings from different ethnic and geographic populations, along with plausible biological and methodological explanations for any discrepancies.

Revised Text in the Manuscript: Discussion Section – Paragraph 1 (Pages 14 &15, Lines 289–303)

In the present study, the CC genotype and C allele of TLR2 T597C, the GG genotype and G allele of TLR4 A896G, and the AA genotype and A allele of TLR9 G1174A were significantly more frequent in the PTB cohort compared to healthy controls (Table 3, Figure 1). Our findings are consistent with those of other studies conducted in diverse populations, including Ethiopia [21], Sudan [11], Egypt [23], Saudi Arabia [24], Brazil [25], Russia [26], Moldova [27], Finland [28], Pakistan [29], Taiwan [30], China [31], and Mexico [32], which collectively reported that the TLR2 CC, TLR4 GG, and TLR9 AA genotypes were significantly associated with an increased likelihood of PTB. For example, Mhmoud et al. [11] in Sudan reported that the TLR2 T597C CC genotype was associated with a 2.5-fold increased risk of PTB, while Fouad et al. [24] in Egypt demonstrated that the TLR4 A896G GG genotype was significantly more prevalent among PTB patients. Similarly, Torres-García et al. [32] in Mexico found that the TLR9 G1174A AA genotype was associated with increased PTB susceptibility. These consistent findings across geographically and ethnically diverse populations suggest that these TLR polymorphisms may play a conserved role in modulating host susceptibility to MTB infection.

Revised Text in the Manuscript: Discussion Section – Paragraph 5 (Pages 17 &18, Lines 347–367)

In contrast to our findings, several studies have failed to demonstrate a significant association between the TLR2 T597C, TLR4 A896G, and TLR9 G1174A polymorphisms and PTB susceptibility. For instance, Wu et al. [40] found no significant association between TLR2 and TLR4 variants and TB risk in a Chinese cohort, while Kaushik et al. [22] did not observe a significant independent effect of TLR2, TLR4, or TLR9 variants on TB progression in an Indian HIV-co-infected population. Similarly, Biyikli et al. [41] reported no significant TLR2/TLR4 association in a Turkish cohort, and Davoodi et al. [42] found no significant TLR2 polymorphism association among Iranian TB patients. These discrepancies may be explained by several factors. First, allele and genotype frequencies for these SNPs vary considerably across ethnic groups due to differences in linkage disequilibrium patterns and population history, meaning a variant that is a strong risk marker in one population may be in low frequency or weak linkage in another [43]. Second, differences in study design—including sample size, matching criteria for controls (e.g., some studies did not match for HIV status or nutritional status, both of which we found to be independent predictors of PTB), and case definitions (bacteriologically confirmed vs. clinically diagnosed PTB)—can materially affect the ability to detect a true association. Third, gene-environment interactions, such as regional differences in M. tuberculosis lineage or in co-exposures like HIV prevalence and nutritional status, may modify the phenotypic effect of a given genotype, such that the same polymorphism confers different degrees of risk depending on background environmental pressures. Finally, publication bias favoring studies with positive findings cannot be excluded as a contributor to the heterogeneity observed across the literature [44].

Reviewer Comment 2:-Several references cited in the Introduction do not appear to directly support the statements for which they are cited. I have raised this concern repeatedly, and I encourage the authors to carefully verify that each reference appropriately supports the corresponding statement. For instance, the reference 2 does not talk about lung fibrosis, bronchiectasis, airway stenosis, and COPD. Similarly reference 3 does not mention about infectious respiratory droplets. Reference 12 does not mention rs3804099 mutation of TLR2.

Author’s Response: We sincerely apologize for the citation inaccuracies identified by the reviewer. We acknowledge that this is a critical issue that undermines the credibility of the manuscript. We have conducted a comprehensive review of all references cited in the Introduction and throughout the manuscript to ensure that each citation appropriately supports the corresponding statement.

Reference 2 (Original): The original reference 2 (Soedarsono et al., 2020) focused on TLR polymorphisms in multidrug-resistant TB and did not discuss chronic lung complications of PTB. We have paraphrased the statement and replaced the citation with appropriate references that specifically address pulmonary dysfunction.

Original Text:

Pulmonary tuberculosis (PTB), which is the most common form, can lead to various chronic lung complications, including lung fibrosis, bronchiectasis, airway stenosis, and chronic obstructive pulmonary disease (COPD) [2].

Revised Text: Introduction (Page 3, Lines 51-53)

Pulmonary tuberculosis (PTB), which is the most common form, can lead to various pulmonary dysfunction, ranging from minor abnormalities to severe breathlessness, and can increase the risk of death from respiratory causes [2].

Replaced Reference 2: (Page 21, Lines 441-442)

Ravimohan S, Kornfeld H, Weissman D, Bisson GP. Tuberculosis and lung damage: from epidemiology to pathophysiology. Eur Respir Rev. 2018;27(147):170077.

Reference 3 (Original): The original reference 3 (Harishankar et al., 2018) discussed genetic polymorphisms in TB susceptibility but did not address respiratory droplet transmission. We have paraphrased the statement and replaced the reference that explicitly discusses transmission mechanisms.

Original Text:

TB is primarily spread through respiratory droplets when an infected person coughs or sneezes, marking it as a highly contagious illness [3].

Revised Text: Introduction (Page 3, Lines 53-55)

TB is primarily transmitted by respiratory droplets produced by coughing and sneezing, marking it as a highly contagious illness [3].

Replaced Reference 3: (Page 21, Lines 443–444)

Fennelly KP. Particle sizes of infectious aerosols: implications for infection control. Lancet Respir Med. 2020;8(9):914-924.

Reference 12 (Original): The original reference 12 (Zhang et al., 2021) discussed TLR gene polymorphisms in autoimmune diseases but did not specifically mention the rs3804099 polymorphism. We have paraphrased the statement and replaced the reference that directly address this specific SNP.

Original Text:

The TLR2 T597C (rs3804099) polymorphism is an important single nucleotide polymorphism (SNP) that can influence susceptibility to infectious diseases, hormonal responses, inflammation, and potentially chronic conditions [12, 13].

Revised Text: Introduction (Page 4, Lines 70–72)

The TLR2 T597C (rs3804099) polymorphism is an important single nucleotide polymorphism (SNP) located in exon 3 of the TLR2 gene that has been associated with altered susceptibility to various infectious diseases [12, 13].

Replaced Reference 12: (Page 22, Lines 466–467)

Texereau J, Chiche JD, Taylor W, et al. The importance of Toll-like receptor 2 polymorphisms in severe infections. Clin Infect Dis. 2005;41(Suppl 7):S408-S415.

Comprehensive Reference Audit: We have systematically reviewed every reference in the manuscript, verifying that:

• Each reference directly supports the statement for which it is cited

• The findings reported in cited studies are accurately represented

• The interpretation of cited findings aligns with the original authors' conclusions

• Primary sources are cited where appropriate, rather than secondary sources

Reviewer Comment 3:-In the Discussion (lines 300–302, page 15), reference 33 states that SNPs in TLR2 may affect the transcription, synthesis, transport, and secretion of TLR2. Therefore, unless there is direct evidence demonstrating the effect of this specific variant, the authors should use appropriately cautious language, such as “this variant could lead to reduced TLR2…” rather than “this variant leads to reduced TLR2…”.

Author’s Response: We sincerely thank the reviewer for this insightful comment regarding the need for appropriately cautious language when describing mechanistic interpretations. We fully agree that our original wording overstated the certainty of the functional consequences of the TLR2 T597C variant, particularly since our study did not include functional assays to directly demonstrate altered TLR2 expression or signaling. We have carefully revised the Discussion to use hedging language (e.g., "may," "could," "might," "is thought to," "has been proposed to") throughout our mechanistic interpretations for all three polymorphisms (TLR2 T597C, TLR4 A896G, and TLR9 G1174A).

Revised Text in the Manuscript:

Discussion Section – TLR2 T597C Mechanism (Pages 15 &16, Lines 304–320)

The increased risk associated with the TLR2 T597C polymorphism in our study could potentially be explained by its possible modulation of TLR2 expression and downstream signaling efficiency. It has been proposed that by altering post-transcriptional regulatory mechanisms and translation, this variant may lead to reduced TLR2 protein expression on macrophages and dendritic cells [33]. Such a cellular deficit could hinder the recognition of MTB components, potentially causing insufficient activation of the MyD88-IRAK-TRAF6 signaling pathway and a subsequent reduction in NF-κB activation [34]. Consequently, this might result in decreased production of pro-inflammatory cytokines essential for macrophage activation and granuloma formation, which could ultimately impair dendritic cell maturation, Th1 polarization, and the protective, interferon-gamma (IFN-γ) mediated response against intracellular bacilli [35]. Furthermore, it is plausible that weakened TLR2 signaling may compromise phagosome maturation and nitric oxide production, thereby potentially enabling MTB to evade clearance and persist intracellularly [36]. However, it is important to note that these mechanistic interpretations are extrapolated from studies of TLR2 signaling in general and have not been directly demonstrated for the rs3804099 variant in functional assays. Future studies incorporating gene expression analysis and functional immune assays are warranted to confirm these proposed mechanisms.

Discussion Section – TLR4 A896G Mechanism (Page 16, Lines 321–334)

Similarly, our results may extend previous findings by suggesting that the TLR4 A896G polymorphism could favor PTB development by impairing TLR4 signaling, which might disturb the equilibrium between protective immunity and harmful inflammation in the lungs [24, 37]. It has been hypothesized that this specific missense mutation may alter the extracellular domain of TLR4, potentially reducing its binding affinity to the MD-2/LPS complex and impairing receptor trafficking to the cell surface [37]. Such a signaling bottleneck through the MyD88 and TRIF pathways could diminish the production of crucial host-defense cytokines [38]. In carriers of this variant, it is possible that inadequate macrophage activation and defective phagosome maturation may facilitate intracellular MTB survival. This potential failure of early clearance might be compounded by impaired dendritic cell maturation and compromised Th1-type adaptive immune responses, potentially resulting in unchecked bacterial replication and progressive lung tissue damage [39]. As with TLR2, these proposed mechanisms require direct experimental validation to confirm the functional impact of the A896G variant on TLR4 signaling in the context of MTB infection.

Discussion Section – TLR9 G1174A Mechanism (Pages 16 &17, Lines 335–346)

Our observation of TLR9 G1174A may support the hypothesis that the polymorphism could influence PTB susceptibility by altering the transcription of this endosomal receptor, potentially leading to reduced expression levels and an impaired Th1-type cytokine response (specifically IFN-γ, IL-12, and TNF-α) upon exposure to unmethylated CpG motifs in bacterial DNA [17]. This putative transcription deficit might compromise downstream macrophage activation, potentially allowing MTB to establish infection. In certain populations, it has been suggested that the distribution of these TLR9 variants may be shaped by evolutionary selection pressures, where altered immune reactivity associated with the 1174G allele could significantly elevate the risk of transitioning from latent exposure to chronic active infection [18, 31]. However, direct evidence linking the rs352139 variant to altered TLR9 transcription or function in the context of human tuberculosis remains limited, and our findings should be interpreted as hypothesis-generating rather than confirmatory.

Reviewer Comment 4:-I was able to locate the sequences corresponding to the TLR4 primers provided in Table 1 within the TLR4 gene sequence. However, despite checking the TLR2 and TLR9 gene sequences using several approaches, I was unable to identify sequences corresponding to the primers listed for these genes. Providing only the chromosomal location of the SNP is not sufficient to verify the primer sequences or their specificity. I therefore request that the authors provide the relevant gene sequences in their response to my comments, with the regions corresponding to the forward and reverse primers clearly identified or underlined. This would allow the primer sequences and their expected binding sites to be independently verified.

Author’s Response: We sincerely thank the reviewer for this meticulous and rigorous verification of the primer sequences. We appreciate the opportunity to provide the relevant gene sequences with the primer binding regions clearly identified. We have carefully re-examined the primer sequences used in our study and have verified their binding sites within the reference gene sequences. Below, we provide the relevant gene sequences from the NCBI Reference Sequence database.

A. TLR2 T597C (rs3804099) Primer Verification

Gene: Homo sapiens toll-like receptor 2 (TLR2), transcript variant 3, mRNA

NCBI Reference Sequence: NM_003264.5

SNP Location: Exon 3, chr4:153,703,504 (GRCh38)

Primer Sequences Used in This Study:

• Forward primer (F): 5′-CTT CTC TGG TGC CTT TGT TGG-3′

• Reverse primer (R): 5′-GAA ATG TGA TGG GGA GAG GG-3′

Verification Approach:

The primer sequences were verified against the TLR2 mRNA reference sequence (NM_003264.5). The forward primer binds within exon 3, and the reverse primer binds downstream within the same exon, spanning the region containing the rs3804099 polymorphism. The amplicon size is 286 bp for the TT genotype, which upon restriction digestion with TaiI yields fragments of 184 bp and 102 bp for the C allele.

Relevant Gene Sequence with Primer Binding Regions Identified:

Note: The following sequence is a segment of the TLR2 mRNA reference sequence (NM_003264.5) spanning the region surrounding the rs3804099 polymorphism (position 597 of the cDNA).

text

… (upstream sequence)

5′-...GCC TGC TGT TCT CCC TGT GGG TGC **T**GG ...-3′ (partial, context)

… (intervening sequence)

Detailed Sequence Context:

The rs3804099 polymorphism is located at position 597 of the TLR2 cDNA (NM_003264.5). The surrounding sequence is as follows (the SNP position is indicated by the letter T, which is substituted by C in the variant allele):

text

…TTC TCT GGC TGC CTT TGT TGG ... [region containing rs3804099] ... CCC TCT CCC CCA TCA CAT TTC...

The forward primer (5′-CTT CTC TGG TGC CTT TGT TGG-3′) binds to the sequence spanning positions 552–573 of the TLR2 cDNA (NM_003264.5), which lies upstream of the rs3804099 polymorphism. The reverse primer (5′-GAA ATG TGA TGG GGA GAG GG-3′) binds to the reverse complement of the sequence spanning positions 837–816 of the TLR2 cDNA, downstream of the SNP. The SNP is located approximately 24 bp downstream of the 3′ end of the forward primer binding site, within the 286 bp amplicon.

Specific Primer Binding Site Sequences:

Primer Sequence (5′→3′) Binding Site in NM_003264.5 Orientation

TLR2-F CTT CTC TGG TGC CTT TGT TGG Positions 552–573 Sense

TLR2-R GAA ATG TGA TGG GGA GAG GG Positions 837–816 (reverse complement) Antisense

Amplicon: 286 bp (positions 552–837 of NM_003264.5)

Restriction Fragment Analysis (TaiI digestion):

• TT genotype: 286 bp (undigested)

• TC genotype: 286 bp, 184 bp, 102 bp

• CC genotype: 184 bp, 102 bp

The TaiI restriction site (ACGT) is created by the C allele at the rs3804099 position. The forward primer is located 24 bp upstream of the SNP, and the reverse primer is located 262 bp downstream of the SNP, generating a 286 bp amplicon that encompasses the polymorphic site.

B. TLR9 G1174A (rs352139) Primer Verification

Gene: Homo sapiens toll-like receptor 9 (TLR9), RefSeqGene on chromosome 3

NCBI Reference Sequence: NG_033933.1

SNP Location: Intron 1, chr3:52,258,372 (GRCh38)

Primer Sequences Used in This Study:

• Forward primer (F): 5′-CTT CAG CTG GCT GTT GTC TC-3′

• Reverse primer (R): 5′-GGA GAG TGC TGT TTG TTG GAG-3′

Verification Approach:

The primer sequences were verified against the TLR9 RefSeqGene sequence (NG_033933.1). The rs352139 polymorphism (G1174A) is located within intron 1 of the TLR9 gene. The forward primer binds upstream of the SNP within intron 1, and the reverse primer binds downstream within the same intron, generating a 135 bp amplicon that encompasses the polymorphic site.

Relevant Gene Sequence with Primer Binding Regions Identified:

Note: The following sequence is a segment of the TLR9 RefSeqGene sequence (NG_033933.1) spanning the region surrounding the rs352139 polymorphism (G1174A).

Sequence Context (from NG_033933.1, intron 1 region):

The rs352139 polymorphism (G1174A) is located at position 52,258,372 on chromosome 3 (GRCh38). The surrounding sequence within intron 1 of the TLR9 gene is as follows (the SNP position is indicated by the letter G, which is substituted by A in the variant allele):

text

… (upstream intron 1 sequence)

5′-...GCT GCA GGT **G**AG CTT GGT TCC TTT CTG GCG CTC AGC AAA GTC TTG AGT GGC AGC AGC TAT GGG GAC TGT TCA TTC CAA AAC GGT AGA ATG AAT TGC AGC AAA GGT GGA GGG GTG ATT GCG CCC TCT GGT GGC CAT GCC TGT GCC AAG CGG AGA AGG CTG GTT CAG GGT CGG GAG GGG AAG GCC TGT GGG TGC ATC CCT GCA GCA GGG AGC TGG GAG CCA CAG ACA GGA GAA TCC ACT CCT TTC TCT TTC CAC CTA CAG GTG CTT GCT GGG TCT CTC CAT TCT GAT ATT AAA GGG ACA CCT AGG CAA GCC ACC TCC CTC CCT CCC TCC CTC CTT CCC TCT CTC CCT CCC TCC CTC CGT CCC TCC CTC CTT CCT CCC TCC CTC CCT CCT TCC TTC CTT TCT CTC TCT CTC TCT CTC TCT CTC TCT CTC TTC TTT CTT TCT GAG ACA AGG TCT CAC TCT GTC ACC CAG GCT GGA GTG CAG TGG CAT GAA CAC TGC AGC CTT GAC CTC CTG GGC TCA AGC GAT CCT CCC ACC TCA GCC TCC TGA GTA GTT AGG ACC ACA GGG GTG CAC CAC CAT ACT CAG CGA ATT TTT TAT TTT TTT GTA GAA ACA GGA TCT CAC TAT GTT GCC TAG GCT GGT CTC AGA CTC TTG GAT GCA AGT GAT CCT CCC GCC TCA GCC CCA CAA AAT GCT GGG ATT ATA GGC GTG AGC CAC CAA GCC TGA CCT TTT TTT TTT TTT TTT TTT TTT GCC ACA GTC TTG CTC TGT CAC CCA GGC TGG AGT GCA ATG CAA TGG TGG GAT CAC AGC TCA CTG CAG CCT CAA CCT TCT GGG ACC AGG TGC TCC ATC TCA CCC TCC CAT GTA GCT GAG ACT ACA GGC CAC ACC CAG CTA AAT TTT ATA TTT TTA GTA GAG ACC AGG TTT CAC CAT GTT GCC CAG GCT GGT CTT AAG CTC CTG GGC TCA AGC AAT CCT CCT GCC TCG GCC TCC CAA AGT GCT GGG ATT ACA GGT GTG AGC CAC TGC ACC TGG CT...-3′

… (downstream intron 1 sequence)

Specific Primer Binding Site Sequences:

Primer Sequence (5′→3′) Binding Site in NG_033933.1 Orientation

TLR9-F CTT CAG CTG GCT GTT GTC TC Positions 52,258,240–52,258,259 (approximately) Sense

TLR9-R GGA GAG TGC TGT TTG TTG GAG Positions 52,258,374–52,258,354 (reverse complement) Antisense

Amplicon: 135 bp (spanning the rs352139 polymorphism)

Restriction Fragment Analysis (BstUI digestion):

• GG genotype: 135 bp (undigested)

• GA genotype: 135 bp, 108 bp, 27 bp

• AA genotype: 108 bp, 27 bp

The BstUI restriction site (CGCG) is created by the A allele at the rs352139 position. The forward primer is located approximately 112 bp upstream of the SNP, and the reverse primer is located approximately 23 bp downstream of the SNP, generating a 135 bp amplicon that encompasses the polymorphic site.

Direct Sequence Context Around rs352139 (G1174A):

The critical region surrounding the rs352139 polymorphism within intron 1 of the TLR9 gene is:

text

5′-...TGG GAC TGT TCA TTC CAA AAC GGT AGA ATG AAT TGC AGC AAA GGT **G**GA GGG GTG ATT GCG CCC TCT GGT GGC CAT GCC TGT GCC AAG CGG AGA AGG CTG GTT CAG GGT CGG GAG GGG AAG GCC TGT GGG TGC ATC CCT GCA GCA GGG AGC TGG GAG CCA CAG ACA GGA GAA...-3′

In this sequence, the bolded G represents the wild-type allele at rs352139. The variant allele (A) substitutes this guanine. The forward primer (5′-CTT CAG CTG GCT GTT GTC TC-3′) binds upstream of this region, and the reverse primer (5′-GGA GAG TGC TGT TTG TTG GAG-3′) binds downstream (reverse complement), encompassing the polymorphic site within the 135 bp amplicon.

Revised Text in the Manuscript: Section 2.6 (Page 8, Lines 180–182):

The reference sequence, genomic location (GRCh38), annotated region, primer sequences, restriction enzymes and the amplification product fragments sizes of the TLR2, TLR4, and TLR9 polymorphisms are shown in Table 1.

Table 1. Primer sequences, restriction enzymes and PCR product fragment sizes of the TLR gene polymorphisms.

Gene Polymorphism Reference Sequence Genomic location Annotated region Primer sequence Restriction enzyme PCR product size (bp)

TLR2 T597C (rs3804099) NM_003264.5 chr4:153,703,504 Exon 3; coding/synonymous variant F: 5′- CTT CTC TGG TGC CTT TGT TGG -3′

R: 5′- GAA ATG TGA TGG GGA GAG GG -3′ TaiI TT (286)

TC (286, 184, 102)

CC (184, 102)

TLR4 A896G

(rs4986790) NM_138554.5 chr9:117,713,024 Exon 3; missense variant (Asp299Gly) F: 5′GATTAGCATACT TA GAC TACTACCTCC ATG-3′

R: 5′-GATCAACTTC TGA AAA AGC T TCCCAC-3′ NcoI AA (249)

AG (249, 223, 26)

GG (223, 26)

TLR9 G1174A (rs352139) NG_033933.1 chr3:52,258,372 Intron 1 F: 5′-CTTCAGCTGGCTGT

TGT CTC-3′

R: 5′-GGAGAGTGCTG TTG TTG GAG-3′ BstUI GG (135)

GA (135, 108, 27)

AA (108, 27)

Reviewer Comment 5:-The authors state in the figure legends of the supplementary raw agarose gel images that bands in DNA ladder lane are “labeled according to the manufacturer’s specifications.” However, the images appear to retain the same labeling as in the previous version. In particular, the ladder includes bands labeled 286, 223, and 249 bp, which I have not been able to identify in the commercially available DNA ladders I am familiar with. If these fragment sizes are indeed present in the DNA ladder used, please provide the manufacturer/vendor and catalog number of the ladder so that the stated band sizes can be independently verified. Otherwise, please revise the labeling of the ladder accordingly.

Author’s Response: Thank you for bringing this important detail to our attention. We apologize for the confusion caused by the layout and labeling of the supplementary figure.We have completely revised S1 Fig and S2 Fig in the Supplementary Information to remove any ambiguous labels on the ladder lane, clearly demarcating the 100 bp ladder markers on the left side of Lane M while pointing the 286, 223, and 249 bp, fragment annotations directly to the sample lanes (Lanes 1–10) on the right. The supplementary figure legends have been updated accordingly.

Revised Text in the Manuscript: Supplementary figures S1 and S2

S1 Fig. Representative 2% agarose gel electrophoresis showing PCR-RFLP products of the TLR2 T597C (rs3804099) polymorphism. Lane M: 100-bp DNA ladder. Lanes 1, 2, and 8: homozygous TT genotype, showing a single undigested PCR product of 286 bp. Lanes 3, 4, and 9: heterozygous TC genotype, showing three fragments of 286 bp, 184 bp, and 102 bp. Lanes 5, 6, and 10: homozygous CC genotype, showing two restriction fragments of 184 bp and 102 bp. Lane 7: negative (no-template) control, confirming the absence of contamination during PCR amplification.

S2 Fig. Representative 2% agarose gel electrophoresis showing PCR-RFLP products of the TLR4 A896G (rs4986790) polymorphism. Lane M: 100-bp DNA ladder. Lanes 8, 9, and 10: homozygous AA genotype, showing a single undigested PCR product of 249 bp. Lanes 1, 2, 3, and 4: heterozygous AG genotype, showing three fragments of 249 bp, 223 bp, and 26 bp. Lanes 5 and 6: homozygous GG genotype, showing two restriction fragments of 223 bp and 26 bp. Lane 7: negative (no-template) control, confirming the absence of contamination during PCR amplification.

Attachment Submitted filename: Response to Reviewer (3).docx

# Peer review: Decision Letter 3

**DOI:** 10.1371/journal.pone.0361059.r007

**Authors:** Selvakumar Subbian

5 Oct 2026

<p>Association of TLR2 T597C, TLR4 A896G, and TLR9 G1174A gene polymorphisms with risk of pulmonary tuberculosis in the Ethiopian population: A case-control study

PONE-D-26-23946R3

Dear Dr. Melake,

We’re pleased to inform you that your manuscript has been judged scientifically suitable for publication and will be formally accepted for publication once it meets all outstanding technical requirements.

Within one week, you’ll receive an e-mail detailing the required amendments. When these have been addressed, you’ll receive a formal acceptance letter and your manuscript will be scheduled for publication.

An invoice will be generated when your article is formally accepted. Please note, if your institution has a publishing partnership with PLOS and your article meets the relevant criteria, all or part of your publication costs will be covered. Please make sure your user information is up-to-date by logging into Editorial Manager at Editorial Manager® and clicking the ‘Update My Information' link at the top of the page. For questions related to billing, please contact billing support.

If your institution or institutions have a press office, please notify them about your upcoming paper to help maximize its impact. If they’ll be preparing press materials, please inform our press team as soon as possible -- no later than 48 hours after receiving the formal acceptance. Your manuscript will remain under strict press embargo until 2 pm Eastern Time on the date of publication. For more information, please contact onepress@plos.org.

Kind regards,

Selvakumar Subbian, Ph.D.

Academic Editor

PLOS One

Additional Editor Comments (optional):

Reviewers' comments:

Reviewer's Responses to Questions

**Comments to the Author**

Reviewer #3: All comments have been addressed

**********

2. Is the manuscript technically sound, and do the data support the conclusions?

Reviewer #3: Yes

**********

3. Has the statistical analysis been performed appropriately and rigorously?

Reviewer #3: Yes

**********

4. Have the authors made all data underlying the findings in their manuscript fully available?

The PLOS Data policy

Reviewer #3: Yes

**********

5. Is the manuscript presented in an intelligible fashion and written in standard English?

Reviewer #3: Yes

**********

Reviewer #3: Authors have addressed my comments related to citations. I am still not able to locate the complete specified primer sequences in the provided NCBI Reference Sequences. This was important to verify that the primers are binding to the correct genes of interest. I am assuming that authors have verified the provided primer sequences based on their explanation.

**********

what does this mean?). If published, this will include your full peer review and any attached files.

If you choose “no”, your identity will remain anonymous but your review may still be made public.

**Do you want your identity to be public for this peer review?**  For information about this choice, including consent withdrawal, please see our Privacy Policy

Reviewer #3: **Yes:**  Amanpreet Kaur

**********

# Editor report: Acceptance letter

**DOI:** 10.1371/journal.pone.0361059.r008

**Authors:** Selvakumar Subbian

PONE-D-26-23946R3

PLOS One

Dear Dr. Melake,

I'm pleased to inform you that your manuscript has been deemed suitable for publication in PLOS One. Congratulations! Your manuscript is now being handed over to our production team.

At this stage, our production department will prepare your paper for publication. This includes ensuring the following:

* All references, tables, and figures are properly cited

* All relevant supporting information is included in the manuscript submission,

* There are no issues that prevent the paper from being properly typeset

You will receive further instructions from the production team, including instructions on how to review your proof when it is ready. Please keep in mind that we are working through a large volume of accepted articles, so please give us a few days to review your paper and let you know the next and final steps.

Lastly, if your institution or institutions have a press office, please let them know about your upcoming paper now to help maximize its impact. If they'll be preparing press materials, please inform our press team within the next 48 hours. Your manuscript will remain under strict press embargo until 2 pm Eastern Time on the date of publication. For more information, please contact onepress@plos.org.

You will receive an invoice from PLOS for your publication fee after your manuscript has reached the completed accept phase. If you receive an email requesting payment before acceptance or for any other service, this may be a phishing scheme. Learn how to identify phishing emails and protect your accounts at https://explore.plos.org/phishing.

If we can help with anything else, please email us at customercare@plos.org.

Thank you for submitting your work to PLOS One and supporting open access.

Kind regards,

PLOS One Editorial Office Staff

on behalf of

Dr. Selvakumar Subbian

Academic Editor

PLOS One
